# Synthesis of Novel Hydrazide–Hydrazone Compounds and *In Vitro* and *In Silico* Investigation of Their Biological Activities against AChE, BChE, and hCA I and II

**DOI:** 10.1021/acsomega.3c10182

**Published:** 2024-04-26

**Authors:** Reşit Çakmak, Eyüp Başaran, Kader Sahin, Murat Şentürk, Serdar Durdağı

**Affiliations:** † Medical Laboratory Techniques Program, Vocational School of Health Services, Batman University, 72000 Batman, Türkiye; ‡ Department of Chemistry and Chemical Processing Technologies, Vocational School of Technical Sciences, Batman University, 72000 Batman, Türkiye; § Department of Analytical Chemistry, School of Pharmacy, Bahcesehir University, 34353 Istanbul, Türkiye; ∥ Department of Biochemistry, Pharmacy Faculty, Ağrı Ibrahim Çecen University, 04100 Ağrı, Türkiye; ⊥ Computational Biology and Molecular Simulations Laboratory, Department of Biophysics, School of Medicine, Bahçeşehir University, 34353 İstanbul, Türkiye; # Lab for Innovative Drugs (Lab4IND), Computational Drug Design Center (HITMER), Bahçeşehir University, 34353 İstanbul, Türkiye; ∇ Molecular Therapy Lab, Department of Pharmaceutical Chemistry, School of Pharmacy, Bahçeşehir University, 34353 Istanbul, Türkiye

## Abstract

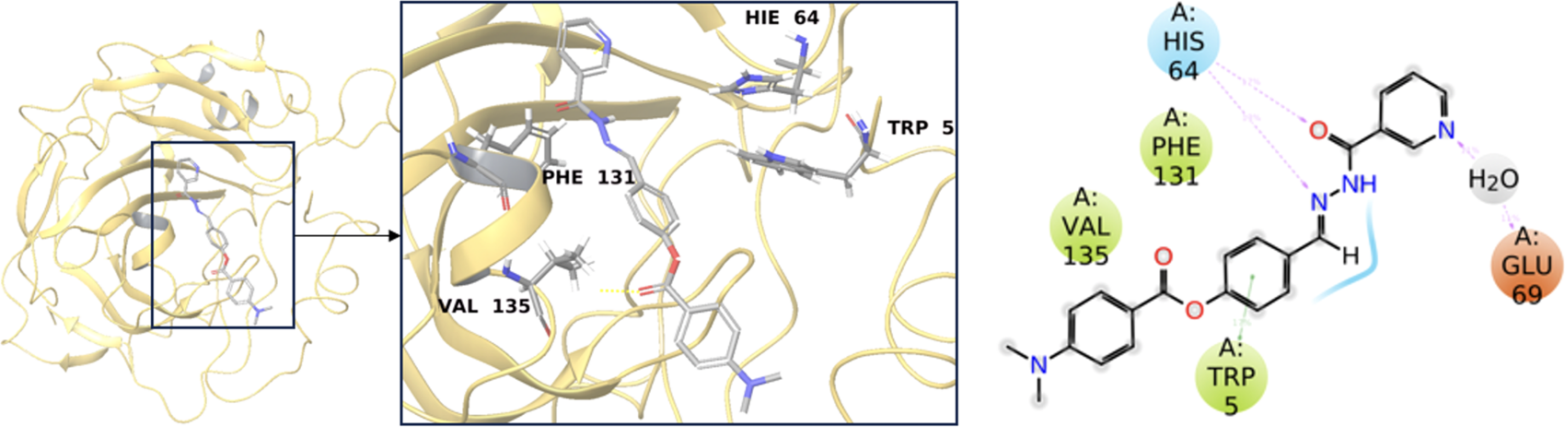

The abnormal levels of the human carbonic anhydrase isoenzymes I and II (hCA I and II) and cholinesterase enzymes, namely, acetylcholinesterase (AChE) and butyrylcholinesterase (BChE), are linked with various disorders including Alzheimer’s disease. In this study, six new nicotinic hydrazide derivatives (**7**–**12**) were designed and synthesized for the first time, and their inhibitory profiles against hCA I, hCA II, AChE, and BChE were investigated by *in vitro* assays and *in silico* studies. The structures of novel molecules were elucidated by using spectroscopic techniques and elemental analysis. These molecules showed inhibitory activities against hCA I and II with IC_50_ values ranging from 7.12 to 45.12 nM. Compared to reference drug acetazolamide (AZA), compound **8** was the most active inhibitor against hCA I and II. On the other hand, it was determined that IC_50_ values of the tested molecules ranged between 21.45 and 61.37 nM for AChE and between 18.42 and 54.74 nM for BChE. Among them, compound **12** was the most potent inhibitor of AChE and BChE, with IC_50_ values of 21.45 and 18.42 nM, respectively. In order to better understand the mode of action of these new compounds, state-of-the-art molecular modeling techniques were also conducted.

## 1 Introduction

Carbonic anhydrases (CAs, 4.2.1.1) are metalloenzymes found in prokaryotes and higher organisms, catalyzing the conversion between carbon dioxide (CO_2_) and bicarbonate (HCO_3_^–^) and containing Zn^2+^ in its structure.^1−3^ CAs are effective in maintaining many vital activities. These enzymes are involved in many significant physiological reactions such as electrolyte and fluid secretion, pH regulation, biosynthetic reactions, and many other physiological or pathological processes.^4,5^ So far, 16 different CA isoforms of physiological importance have been isolated and identified.^6,7^ Although the human carbonic anhydrase isoenzyme I (hCA I) is more abundant in erythrocytes than human carbonic anhydrase isoenzyme II (hCA II), its catalytic activity is low, and hCA II is distributed in various tissues. hCA II is the only cytosolic isoenzyme that shows catalytic activity at maximum rate compared to other isoenzymes for CO_2_ hydration reaction.^8−12^ Because of this property, hCA II is one of the most studied isoforms of CAs. This enzyme is abundantly found in different parts of the human body.^12^ It also plays a role in transporting sodium ions to the eye tissue and regulating intraocular pressure.^13^ hCA isoenzymes are therapeutic targets that are prone to be inhibited in the therapy of various disorders including glaucoma. They are also used for the therapy of various diseases such as cancer, obesity, epilepsy, arthritis, neuropathic pain, Alzheimer’s disease (AD), and osteoporosis.^5,9^

AD is a progressive and fatal neurodegenerative disease characterized by impairment in the person’s ability to perform daily activities and cognitive dysfunction in the later stages of the disease.^2,14−18^ AD is the most common cause of dementia seen in the elderly, accounting for an estimated 60–70% of cases worldwide. With aging of our world population, the prevalence of dementia would increase globally. The World Health Organization (WHO) reported that the number of people with dementia could exceed 80 and 150 million by 2030 and 2050, respectively.^19^

**Figure 1:**
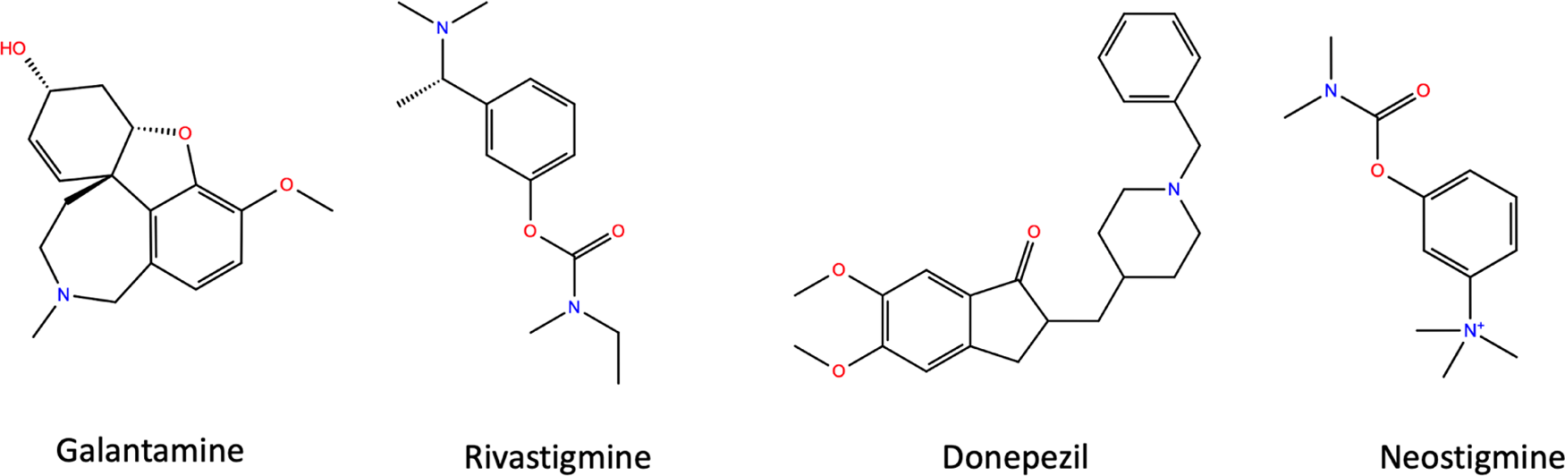
Two-dimensional (2D) chemical structures of the four well-known ChEIs galantamine, rivastigmine, neostigmine, and donepezil.

Today, several U.S. Food and Drug Administration (FDA)-approved drugs such as memantine act as NMDA receptor antagonists, which increase the therapeutic effect when used together with cholinesterase inhibitors (ChEIs) such as galantamine, rivastigmine, and donepezil, which are used in the treatment of AD^20,21^ (Figure 1). Neostigmine, another clinical ChEI, is primarily prescribed for managing myasthenia gravis, a neuromuscular disorder marked by muscle weakness and fatigue. By inhibiting the activity of acetylcholinesterase (AChE), neostigmine boosts acetylcholine levels at synapses, thereby extending and amplifying cholinergic neurotransmission (Figure 1). While these drugs provided a therapeutic effect of less than 50% in reducing symptoms and delaying progression in Alzheimer’s patients in the early stages, they had almost no success in patients in advanced stages. Moreover, these drugs on the market may cause serious side effects owing to their limited therapeutic effects, low bioavailability, off-target specificity, and high hepatotoxicity values.^22,23^ Therefore, studies on design and synthesis of new therapeutics are needed to treat AD.^24^

Together with the well-known roles of AChE and butyrylcholinesterase (BChE) enzymes at the AD, it has also been shown that CA inhibitors reduce amyloid β pathology and improve cognition by ameliorating cerebrovascular health and glial fitness.^25^ Studies have demonstrated that CA inhibitors such as acetazolamide (AZA) and methazolamide (MTZ) mitigate mitochondrial dysfunction and apoptosis triggered by amyloid β in both vascular and neural cells. This effect is achieved by diminishing the production of reactive oxygen species (ROS) within the mitochondria and preventing the decline of mitochondrial membrane potential.^26^

Thus, in the current study, we aimed to target four enzymes (AChE, BChE, and hCA I and II) with newly synthesized compounds. The rationale behind targeting both CAs and cholinesterases lies in their involvement in overlapping pathways implicated in various diseases including neurological disorders. While dual inhibition may initially appear counterintuitive, it can offer several potential advantages. First, certain diseases, such as AD, involve multifaceted pathological mechanisms where targeting multiple enzymes concurrently may lead to enhanced therapeutic efficacy. Additionally, there may be synergistic effects between the inhibition of different enzyme classes, leading to improved outcomes compared to those of single-target inhibition.

Introducing the hydrazone moiety alongside aryl esters may enhance the binding affinity of the compound for the targeted enzymes. The hydrazone functionality can form additional hydrogen bonds or π–π stacking interactions with key residues, leading to stronger enzyme–inhibitor interactions. The presence of both aryl ester and hydrazone functionalities in a single compound provides versatility in exploring a diverse chemical space. This allows for the systematic optimization of the inhibitor’s structure to maximize its inhibitory activity against the target enzymes while minimizing undesirable interactions with unrelated biological targets. Combining aryl ester and hydrazone moieties in a single compound enables the potential for multimodal inhibition. This means that the inhibitor may target multiple sites or mechanisms within the enzyme, leading to synergistic effects and enhanced overall efficacy in inhibiting enzyme activity. Hydrazone compounds often exhibit improved stability and pharmacokinetic properties compared with their corresponding aryl esters. The presence of the hydrazone moiety can enhance the compound’s metabolic stability, bioavailability, and duration of action, resulting in more sustained inhibition of enzyme activity.

Hydrazone compounds constitute an important class of organic compounds for the design of novel active drugs owing to their significant biological and pharmacological properties. These compounds are also used as organic intermediates in the development of new bioactive molecules due to the azomethine moiety in their structure.^27,28^ Hydrazone fragments bound to heterocyclic systems exhibit increased activity due to their ability to form hydrogen-bonding interactions with molecular targets. This discovery has been particularly important in the field of medicinal chemistry.^29,30^ These compounds have attracted considerable attention owing to their antimicrobial, anticancer, antiviral, antitubercular, anti-inflammatory, antioxidants, antiproliferative, and α-glucosidase activities.^25−32^ Moreover, many researchers have reported that they are potent inhibitors against some metabolic enzymes including hCA I, hCA II, AChE, and BChE.^9,25,32,33^

In light of the above-mentioned findings and as a continuation of our studies, herein we aimed to evaluate the inhibition effects of newly synthesized hydrazone derivatives (**7**–**12**) toward four metabolic enzymes (hCA I, hCA II, AChE, BChE), linked to various diseases, including AD. The molecular hybridization method, a novel drug design and discovery method based on the combination of pharmacophoric moieties of many biologically active molecules to synthesize new hybrid molecules with better affinity and activity compared to parent drugs, was used to discover new potential inhibitor candidates. In this study, we designed and synthesized novel hybrid molecules containing three biologically active key structural motifs (a dimethylamine moiety, such as rivastigmine used in the treatment of AD, a pyridine ring, and hydrazone moieties), and then their enzyme inhibitory activities were investigated by *in vitro* assays. The chemical structures of all synthesized molecules were characterized by elemental analysis and some spectral techniques including Fourier transform infrared (FT-IR), ^1^H NMR, and ^13^C NMR. In addition, in order to support the results obtained as a result of the enzyme inhibition assay studies, molecular modeling approaches were also carried out.

## 2 Materials and Methods

### 2.1 General

All chemicals and solvents used in this study to discover novel inhibitors of AChE, BChE, and hCA I and II enzymes were of analytical grade and high purity and were purchased from Sigma, Aldrich, Merck, and Alfa Aesar. Silica gel 60 F_254_ from Merck was used to monitor the reaction progress. Barnstead IA9100 Electrothermal and Stuart SMP30 Digital Melting Points Apparatus was employed to determine the melting points. All tested molecules were characterized by elemental analysis (Thermo Scientific Flash 2000 CHNS elemental analyzer) and three spectroscopic techniques, including FT-IR (Cary 630 FT-IR spectrometer), ^1^H NMR, and ^13^C NMR (Bruker Avance III 400 MHz spectrometer).

### 2.2 General Procedures for the Synthesis of Aryl Esters

A solution of a suitable phenolic aldehyde (2.0 mmol) in pyridine (5 mL) was slowly dropwise added to a solution of 4-(dimethylamino)benzoyl chloride (2.0 mmol) in pyridine (5 mL).^2^ The reaction mixture was stirred continuously at 115 °C for 2 h, and then, the mixture was poured into ice water. The precipitate formed was collected by vacuum filtration and washed with distilled water. Finally, the residue was crystallized from ethanol to afford the product.

#### 2.2.1 2-Formylphenyl 4-(Dimethylamino)benzoate (**1**)^34^

White solid, yield: 79%, mp 142 °C. FT-IR (cm^–1^, υ_max_): 3072 (C–H_arom._), 2904 (C–H_aliph._), 2846, 2754 (C–H_aldehyde_), 1707 (C=O_ester_), 1685 (C=O_aldehyde_). ^1^H NMR (CHCl_3_-*d*, 400 MHz, ppm): δ 10.27 (s, 1H, −CHO), 8.08 (d, *J* = 8.8 Hz, 2H, ArH), 7.95 (dd, *J* = 7.8, 1.8 Hz, 1H, ArH), 7.68–7.62 (m, 1H, ArH), 7.40–7.35 (m, 1H, ArH), 7.34–7.31 (m, 1H, ArH), 6.71 (d, *J* = 9.0 Hz, 2H, ArH), 3.09 (s, 6H, CH_3_ × 2). ^13^C NMR (CHCl_3_-*d*, 100 MHz, ppm): 188.67 (HC=O), 165.16 (C=O), 154.03, 153.44, 135.23, 132.23, 128.92, 128.57, 125.92, 123.77, 114.62, 110.90 (ArC), 40.05 (CH_3_ × 2). Anal. Calcd for C_16_H_15_NO_3_: C, 71.36; H, 5.61; N, 5.20%. Found: C, 71.41; H, 5.63; N, 5.29%.

#### 2.2.2 4-Formylphenyl 4-(Dimethylamino)benzoate (**2**)^35^

White solid, yield: 75%, mp 167–169 °C. FT-IR (cm^–1^, υ_max_): 3099 (C–H_arom._), 2903 (C–H_aliph._), 2827, 2744 (C–H_aldehyde_), 1715 (C=O_ester_), 1687 (C=O_aldehyde_). ^1^H NMR (CHCl_3_-*d*, 400 MHz, ppm): δ 10.00 (s, 1H, −CHO), 8.04 (d, *J* = 9.2 Hz, 2H, ArH), 7.94 (d, *J* = 8.7 Hz, 2H, ArH), 7.39 (d, *J* = 8.4 Hz, 2H, ArH), 6.69 (d, *J* = 9.0 Hz, 2H, ArH), 3.08 (s, 6H, CH_3_ × 2). ^13^C NMR (CHCl_3_-*d*, 100 MHz, ppm): 191.11 (HC=O), 164.74 (C=O), 156.36, 153.94, 133.59, 132.15, 131.16, 122.70, 115.00, 110.80 (ArC), 40.04 (CH_3_ × 2). Anal. Calcd for C_16_H_15_NO_3_: C, 71.36; H, 5.61; N, 5.20%. Found: C, 71.32; H, 5.61; N, 5.34%.

#### 2.2.3 2-Formyl-6-methoxyphenyl 4-(Dimethylamino)benzoate (**3**)

White solid, yield: 73%, mp 170–171 °C. FT-IR (cm^–1^, υ_max_): 3093 (C–H_arom._), 2929 (C–H_aliph._), 2834, 2761 (C–H_aldehyde_), 1715 (C=O_ester_), 1691 (C=O_aldehyde_). ^1^H NMR (CHCl_3_-*d*, 400 MHz, ppm): δ 10.24 (s, 1H, −CHO), 8.09 (d, *J* = 9.1 Hz, 2H, ArH), 7.52 (dd, *J* = 7.8, 1.5 Hz, 1H, ArH), 7.32 (t, *J* = 8.0 Hz, 1H, ArH), 7.28–7.19 (m, 1H, ArH), 6.72 (d, *J* = 9.1 Hz, 2H, ArH), 3.84 (s, 3H, OCH_3_), 3.09 (s, 6H, CH_3_ × 2). ^13^C NMR (CHCl_3_-*d*, 100 MHz, ppm): 188.90 (HC=O), 164.73 (C=O), 153.97, 152.21, 143.37, 132.33, 129.82, 126.39, 119.60, 117.81, 114.61, 110.86 (ArC), 56.34 (OCH_3_), 40.06 (CH_3_ × 2). Anal. Calcd for C_17_H_17_NO_4_: C, 68.22; H, 5.72; N, 4.68%. Found: C, 68.28; H, 5.74; N, 4.60%.

#### 2.2.4 5-Formyl-2-methoxyphenyl 4-(Dimethylamino)benzoate (**4**)

Light yellow solid, yield: 76%, mp 141–142 °C. FT-IR (cm^–1^, υ_max_): 3054 (C–H_arom._), 3010 (C–H_aliph._), 2825, 2755 (C–H_aldehyde_), 1711 (C=O_ester_), 1678 (C=O_aldehyde_). ^1^H NMR (CHCl_3_-*d*, 400 MHz, ppm): δ 9.88 (s, 1H, −CHO), 8.06 (d, *J* = 9.0 Hz, 2H, ArH), 7.77 (dd, *J* = 8.4, 2.0 Hz, 1H, ArH), 7.69 (d, *J* = 2.0 Hz, 1H, ArH), 7.09 (d, *J* = 8.5 Hz, 1H, ArH), 6.70 (d, *J* = 9.1 Hz, 2H, ArH), 3.88 (s, 3H, OCH_3_), 3.07 (s, 6H, CH_3_ × 2). ^13^C NMR (CHCl_3_-*d*, 100 MHz, ppm): 190.25 (HC=O), 164.75 (C=O), 156.95, 153.83, 140.89, 132.18, 129.96, 129.63, 124.13, 115.14, 112.00, 110.78 (ArC), 56.23 (OCH_3_), 40.05 (CH_3_ × 2). Anal. Calcd for C_17_H_17_NO_4_: C, 68.22; H, 5.72; N, 4.68%. Found: C, 68.19; H, 5.71; N, 4.73%.

#### 2.2.5 4-Formyl-2-methoxyphenyl 4-(Dimethylamino)benzoate (**5**)

White solid, yield: 72%, mp 142–143 °C. FT-IR (cm^–1^, υ_max_): 3077 (C–H_arom._), 2915 (C–H_aliph._), 2826, 2734 (C–H_aldehyde_), 1715 (C=O_ester_), 1692 (C=O_aldehyde_). ^1^H NMR (CHCl_3_-*d*, 400 MHz, ppm): δ 9.95 (s, 1H, −CHO), 8.06 (d, *J* = 8.9 Hz, 2H, ArH), 7.53–7.48 (m, 2H, ArH), 7.33 (d, *J* = 7.8 Hz, 1H, ArH), 6.69 (d, *J* = 8.9 Hz, 2H, ArH), 3.87 (s, 3H, OCH_3_), 3.07 (s, 6H, CH_3_ × 2). ^13^C NMR (CHCl_3_-*d*, 100 MHz, ppm): 191.24 (HC=O), 164.47 (C=O), 153.86, 152.48, 145.91, 134.86, 132.24, 124.85, 123.83, 115.05, 110.78 (ArC), 56.10 (OCH_3_), 40.05 (CH_3_ × 2). Anal. Calcd for C_17_H_17_NO_4_: C, 68.22; H, 5.72; N, 4.68%. Found: C, 68.19; H, 5.71; N, 4.73%.

#### 2.2.6 1-Formylnaphthalen-2-yl 4-(Dimethylamino)benzoate (**6**)

Yellow solid, yield: 77%, mp 167 °C. FT-IR (cm^–1^, υ_max_): 3057 (C–H_arom._), 2993 (C–H_aliph._), 2805, 2784 (C–H_aldehyde_), 1719 (C=O_ester_), 1676 (C=O_aldehyde_). ^1^H NMR (CHCl_3_-*d*, 400 MHz, ppm): δ 10.77 (s, 1H, −CHO), 9.27 (d, *J* = 8.6 Hz, 1H, ArH), 8.12–8.09 (m, 3H, ArH), 7.88 (d, *J* = 8.1 Hz, 1H, ArH), 7.70–7.66 (m, 1H, ArH), 7.57–7.53 (m, 1H, ArH), 7.40 (d, *J* = 8.9 Hz, 1H, ArH), 6.72 (d, *J* = 9.1 Hz, 2H, ArH), 3.09 (s, 6H, CH_3_ × 2). ^13^C NMR (CHCl_3_-*d*, 100 MHz, ppm): 190.90 (HC=O), 165.17 (C=O), 156.28, 154.11, 136.33, 132.32, 131.55, 130.99, 129.51, 128.36, 126.41, 125.50, 122.21, 121.79, 114.37, 110.91 (ArC), 40.05 (CH_3_ × 2). Anal. Calcd for C_20_H_17_NO_3_: C, 75.22; H, 5.37; N, 4.39%. Found: C, 75.25; H, 5.41; N, 4.44%.

### 2.3 General Procedures for the Synthesis of Hydrazone Derivatives

An aryl ester (2.0 mmol) dissolved in ethanol (5 mL) was dropwise added to a solution of nicotinic hydrazide (2.0 mmol) dissolved in ethanol (5 mL).^27,31^ The reaction mixture was continuously stirred under reflux conditions for 4 h, and then it was cooled to room temperature. The separated crude product was collected by filtration. It was then washed with water and cold alcohol and crystallized from ethanol to give the product.

#### 2.3.1 2-((2-Nicotinoylhydrazono)methyl)phenyl 4-(Dimethylamino)benzoate (**7**)

White solid, yield: 73%, mp 226–227 °C. FT-IR (cm^–1^, υ_max_): 3277 (N–H), 3070 (C–H_arom._), 2903 (C–H_aliph._), 1676 (C=O_ester_), 1665 (C=O_hydrazone_), 1594 (C=N). ^1^H NMR (dimethyl sulfoxide (DMSO)-*d*_6_, 400 MHz, ppm): δ 12.06 (s, 1H, −NH), 9.00 (s, 1H, Py-H), 8.72 (d, *J* = 4.7 Hz, 1H, Py-H), 8.53 (s, 1H, −CH=N), 8.19 (d, *J* = 8.0 Hz, 1H, Py-H), 8.05 (d, *J* = 7.7 Hz, 1H, ArH), 7.97 (d, *J* = 8.9 Hz, 2H, ArH), 7.53–7.49 (m, 2H, ArH and Py-H), 7.38 (t, *J* = 7.5 Hz, 1H, ArH), 7.27 (d, *J* = 8.0 Hz, 1H, ArH), 6.80 (d, *J* = 9.1 Hz, 2H, ArH), 3.03 (s, 6H, CH_3_ × 2). ^13^C NMR (DMSO-*d*_6_, 100 MHz, ppm): 165.09 (C=O), 162.22 (C=O_hydrazone_), 149.00 (C=N), 154.43, 152.72, 150.42, 142.91, 135.95, 132.28, 131.67, 129.56, 127.45, 126.64, 126.24, 124.04, 124.01, 114.28, 111.49 (ArC and Py-C), 40.10 (CH_3_ × 2). Anal. Calcd for C_22_H_20_N_4_O_3_: C, 68.03; H, 5.19; N, 14.42%. Found: C, 68.01; H, 5.24; N, 14.35%.

#### 2.3.2 4-((2-Nicotinoylhydrazono)methyl)phenyl 4-(Dimethylamino)benzoate (**8**)

White solid, yield: 73%, mp 251–252 °C. FT-IR (cm^–1^, υ_max_): 3294 (N–H), 3060 (C–H_arom._), 2904 (C–H_aliph._), 1706 (C=O_ester_), 1661 (C=O_hydrazone_), 1608 (C=N). ^1^H NMR (DMSO-*d*_6_, 400 MHz, ppm): δ 12.03 (s, 1H, −NH), 9.07 (s, 1H, Py-H), 8.75 (d, *J* = 3.6 Hz, 1H, Py-H), 8.47 (s, 1H, −CH=N), 8.25 (d, *J* = 7.9 Hz, 1H, Py-H), 7.92 (d, *J* = 8.9 Hz, 2H, ArH), 7.81 (d, *J* = 8.5 Hz, 2H, ArH), 7.57–7.54 (m, 1H, Py-H), 7.32 (d, *J* = 8.5 Hz, 2H, ArH), 6.77 (d, *J* = 9.0 Hz, 2H, ArH), 3.02 (s, 6H, CH_3_ × 2). ^13^C NMR (DMSO-*d*_6_, 100 MHz, ppm): 164.89 (C=O), 162.14 (C=O_hydrazone_), 149.05 (C=N), 154.23, 152.91, 152.74, 148.08, 135.90, 132.05, 131.92, 129.63, 128.77, 124.05, 123.08, 114.63, 111.39 (ArC and Py-C), 40.04 (CH_3_ × 2). Anal. Calcd for C_22_H_20_N_4_O_3_: C, 68.03; H, 5.19; N, 14.42%. Found: C, 68.06; H, 5.17; N, 14.49%.

#### 2.3.3 2-Methoxy-6-((2-nicotinoylhydrazono)methyl)phenyl 4-(Dimethylamino)benzoate (**9**)

White solid, yield: 73%, mp 249–250 °C. FT-IR (cm^–1^, υ_max_): 3200 (N–H), 3009 (C–H_arom._), 2912 (C–H_aliph._), 1707 (C=O_ester_), 1685 (C=O_hydrazone_), 1596 (C=N). ^1^H NMR (DMSO-*d*_6_, 400 MHz, ppm): δ 12.04 (s, 1H, −NH), 9.00 (s, 1H, Py-H), 8.71 (d, *J* = 4.8 Hz, 1H, Py-H), 8.52 (s, 1H, −CH=N), 8.21–8.18 (m, 1H, Py-H), 7.95 (d, *J* = 9.0 Hz, 2H, ArH), 7.60 (d, *J* = 7.8 Hz, 1H, ArH), 7.52–7.59 (m, 1H, Py-H), 7.32 (t, *J* = 8.1 Hz, 1H, ArH), 7.21 (d, *J* = 7.5 Hz, 1H, ArH), 6.80 (d, *J* = 9.1 Hz, 2H, ArH), 3.75 (s, 3H, OCH_3_), 3.03 (s, 6H, CH_3_ × 2). ^13^C NMR (DMSO-*d*_6_, 100 MHz, ppm): 164.47 (C=O), 162.16 (C=O_hydrazone_), 149.01 (C=N), 154.40, 152.74, 152.10, 142.98, 139.78, 135.91, 132.29, 129.51, 128.51, 126.98, 123.99, 117.33, 114.39, 114.22, 111.49 (ArC and Py-C), 56.46 (OCH_3_) 40.10 (CH_3_ × 2). Anal. Calcd for C_23_H_22_N_4_O_4_: C, 66.02; H, 5.30; N, 13.39%. Found: C, 66.00; H, 5.33; N, 13.32%.

#### 2.3.4 2-Methoxy-5-((2-nicotinoylhydrazono)methyl)phenyl 4-(Dimethylamino)benzoate (**10**)

White solid, yield: 71%, mp 216 °C. FT-IR (cm^–1^, υ_max_): 3249 (N–H), 3038 (C–H_arom._), 2910 (C–H_aliph._), 1688 (C=O_ester_), 1660 (C=O_hydrazone_), 1601 (C=N). ^1^H NMR (DMSO-*d*_6_, 400 MHz, ppm): δ 11.97 (s, 1H, −NH), 9.05 (s, 1H, Py-H), 8.74 (d, *J* = 3.2 Hz, 1H, Py-H), 8.38 (s, 1H, −CH=N), 8.24–8.22 (m, 1H, Py-H), 7.91 (d, *J* = 9.0 Hz, 2H, ArH), 7.63 (dd, *J* = 8.6, 2.0 Hz, 1H, ArH), 7.57–7.52 (m, 2H, ArH and Py-H), 7.23 (d, *J* = 8.6 Hz, 1H, ArH), 6.77 (d, *J* = 9.1 Hz, 2H, ArH), 3.80 (s, 3H, OCH_3_), 3.02 (s, 6H, CH_3_ × 2). ^13^C NMR (DMSO-*d*_6_, 100 MHz, ppm): 164.53 (C=O), 162.01 (C=O_hydrazone_), 149.02 (C=N), 154.40, 154.20, 153.46, 152.67, 147.92, 140.50, 135.88, 132.06, 129.68, 127.45, 126.71, 124.03, 121.94, 114.62, 113.36, 111.40 (ArC and Py-C), 56.46 (OCH_3_) 40.06 (CH_3_ × 2). Anal. Calcd for C_23_H_22_N_4_O_4_: C, 66.02; H, 5.30; N, 13.39%. Found: C, 66.05; H, 5.27; N, 13.46%.

#### 2.3.5 2-Methoxy-4-((2-nicotinoylhydrazono)methyl)phenyl 4-(Dimethylamino)benzoate (**11**)

White solid, yield: 79%, mp 230–231 °C. FT-IR (cm^–1^, υ_max_): 3186 (N–H), 3007 (C–H_arom._), 2911 (C–H_aliph._), 1704 (C=O_ester_), 1636 (C=O_hydrazone_), 1599 (C=N). ^1^H NMR (DMSO-*d*_6_, 400 MHz, ppm): δ 12.06 (s, 1H, −NH), 9.08 (s, 1H, Py-H), 8.76 (d, *J* = 3.4 Hz, 1H, Py-H), 8.46 (s, 1H, −CH=N), 8.25 (d, *J* = 8.0 Hz, 1H, Py-H), 7.90 (d, *J* = 8.9 Hz, 2H, ArH), 7.57–7.52 (m, 1H, Py-H), 7.49 (s, 1H, ArH), 7.33 (d, *J* = 6.9 Hz, 1H), 7.26 (d, *J* = 8.1 Hz, 1H, ArH), 6.76 (d, *J* = 9.0 Hz, 2H, ArH), 3.81 (s, 3H, OCH_3_), 3.01 (s, 6H, CH_3_ × 2). ^13^C NMR (DMSO-*d*_6_, 100 MHz, ppm): 164.45 (C=O), 162.19 (C=O_hydrazone_), 149.05 (C=N), 154.20, 152.76, 152.08, 148.38, 142.02, 135.92, 133.20, 132.07, 129.66, 124.18, 124.07, 121.17, 114.57, 111.40, 110.29 (ArC and Py-C), 56.32 (OCH_3_) 40.06 (CH_3_ × 2). Anal. Calcd for C_23_H_22_N_4_O_4_: C, 66.02; H, 5.30; N, 13.39%. Found: C, 65.99; H, 5.31; N, 13.35%.

#### 2.3.6 1-((2-Nicotinoylhydrazono)methyl)naphthalen-2-yl 4-(Dimethylamino)benzoate (**12**)

Light yellow solid, yield: 74%, mp 239–240 °C. FT-IR (cm^–1^, υ_max_): 3203 (N–H), 3052 (C–H_arom._), 2904 (C–H_aliph._), 1706 (C=O_ester_), 1649 (C=O_hydrazone_), 1594 (C=N). ^1^H NMR (DMSO-*d*_6_, 400 MHz, ppm): δ 12.17 (s, 1H, −NH), 9.48 (d, *J* = 8.6 Hz, 1H, ArH), 9.05 (s, 1H, Py-H), 8.93 (s, 1H, −CH=N), 8.74 (d, *J* = 5.0 Hz, 1H, Py-H), 8.26–8.21 (m, 1H, ArH), 8.08 (d, *J* = 8.9 Hz, 1H, Py-H), 8.02 (dd, *J* = 8.5, 4.8 Hz, 3H, ArH), 7.69 (t, *J* = 7.3 Hz, 1H, ArH), 7.60 (t, *J* = 7.2 Hz, 1H, ArH), 7.55–7.52 (m, 1H, Py-H), 7.42 (d, *J* = 8.9 Hz, 1H, ArH), 6.81 (d, *J* = 9.1 Hz, 2H, ArH), 3.03 (s, 6H, CH_3_ × 2). ^13^C NMR (DMSO-*d*_6_, 100 MHz, ppm): 165.25 (C=O), 162.36 (C=O_hydrazone_), 149.11 (C=N), 154.47, 152.76, 150.73, 144.82, 136.00, 132.50, 132.43, 132.12, 130.79, 129.60, 129.11, 128.62, 127.06, 126.52, 124.03, 122.84, 121.00, 114.40, 111.49 (ArC and Py-C), 40.11 (CH_3_ × 2). Anal. Calcd for C_26_H_22_N_4_O_3_: C, 71.22; H, 5.06; N, 12.78%. Found: C, 71.26; H, 5.07; N, 12.85%.

### 2.4 Biological Assay

In this study, hCA I and II were obtained from fresh human erythrocytes using affinity chromatography as previously described in the literature.^36,37^ AChE from *Electrophorus electricus* (electric eel) (C3389: Sigma-Aldrich), BChE from equine serum (C4290: Sigma-Aldrich), and the other chemicals (4-nitrophenyl acetate (N8130: Sigma-Aldrich), 5,5′-dithiobis(2-nitrobenzoic acid) (D218200: Sigma-Aldrich), acetylthiocholine iodide (A5771: Sigma-Aldrich), butyrylthiocholine iodide (B3253: Sigma-Aldrich)) were purchased from local representatives of well-known commercial companies. On the other hand, the absorbance rates of each compound were determined by a microplate spectrophotometer (Multiskan Go, Thermo Scientific).

#### 2.4.1 hCA I and II Activity Assays

In this study, we conducted hCA I and II activity assays using the Verpoorte method, as outlined in previous literature.^38^ The inhibitory activities of all of the newly synthesized compounds were thoroughly tested. The inhibitory activity of each molecule was examined in triplicate at each concentration. Different concentrations were employed for all inhibitors tested in this study, respectively. The baseline activity in the control cuvette, without any inhibitory agent, was established as 100%. AZA was used as a positive control compound. Notably, the study’s findings were represented through individual graphs delineating the activity percentages in relation to inhibitor concentrations, as elucidated by prior research sources.^2,18,39,40^

#### 2.4.2 AChE and BChE Activity Assays

The evaluation of the inhibitory characteristics of all synthesized compounds against AChE and BChE was conducted using the widely recognized Ellman method,^41^ as described in pertinent literature sources.^2,14,18^ Throughout this study, neostigmine and rivastigmine served as the reference compounds for the AChE and BChE assays.

### 2.5 Molecular Modeling Studies

To gain deeper insights into the molecular mechanisms of the synthesized compounds at the target enzymes under investigation, molecular modeling studies were utilized. These studies were employed to enhance our comprehension of the structural and dynamic characteristics of small molecules interacting with target enzymes. Throughout these simulations, we utilized a diverse array of techniques including molecular docking, molecular dynamics (MD) simulations, and binding free energy calculations. This comprehensive approach allowed us to thoroughly explore and analyze the structural and dynamic properties of the compounds. By employing these methodologies, we gained valuable insights into the atomic-level interactions between the selected compounds and the target proteins. Additionally, we successfully predicted both the binding affinity and binding mode of these compounds, thereby enhancing our comprehension of their pharmacological potential.^42,43^

#### 2.5.1 Preparation of Systems

The protonation states of studied compounds were created at neutral pH using Epik.^44^ The OPLS3e force field was used with default parameters to perform structural optimization for each compound.^45^ The crystal structure of *E. electricus* acetylcholinesterase (PDB ID: 1C2B), hCA I (PDB ID: 4WR7), and hCA II (PDB ID: 5AML) were used from Protein Data Bank (PDB). For the equine butyrylcholinesterase, the *fasta* sequence of EqBChE (UniProtKB entry P81908) was submitted to the Swiss Model server for construction of the model. Then, using Schrodinger’s Maestro molecular modeling package, the structures of the template (hBChE, 4BDS) and model proteins were aligned, and the tacrine coordinates were copied from hBChE to EqBChE. The protein preparation module of the Maestro molecular modeling package was used for the preparation of target protein structures before the docking processes. Hydrogen atoms were added to the target enyzmes using PROPKA at physiological pH (i.e., 7.4) to define correct ionization states of amino acid residues.

#### 2.5.2 Molecular Docking

Three different molecular docking algorithms were utilized including standard precision (SP), quantum-polarized ligand docking (QPLD),^46^ and induced-fit docking (IFD).^47^ In QPLD, initially, Glide/SP was applied, and top-docking poses were used in quantum mechanics (QM) charge calculations, which use the 6-31G* basis set, B3LYP density functional, and “ultrafine” SCF accuracy level. In IFD protocol, initially, Glide/SP was performed, and 5.0 Å around the low energy docking poses was used in geometry optimization. The refined binding pocket with energy minimization was then used in the re-docking of compounds with the Glide/XP protocol.

#### 2.5.3 MD Simulations and Binding Free Energy Calculations

MD simulations were initiated by the IFD poses. The MD simulations utilized the top-docking positions as input coordinates. For solvation, TIP3P water models were employed, extending 10.0 Å from the protein edges to determine the solvation box. 200 ns MD simulations were carried out using the Desmond program, following a methodology consistent with our previously reported studies.^48^

The Prime module was used for molecular mechanics/generalized Born surface area (MM/GBSA) binding free energy calculations of the selected ligand–protein complexes. Since docking poses were not significantly changing throughout MD simulations initiated by IFD poses, whole trajectories were used in MM/GBSA calculations.^49^ The OPLS3e force field and VSGB 2.0 solvation model were used in order to predict the free binding energies of complexes.^50^

## 3 Results and Discussion

### 3.1 Synthesis and Characterization

**Scheme 1:**
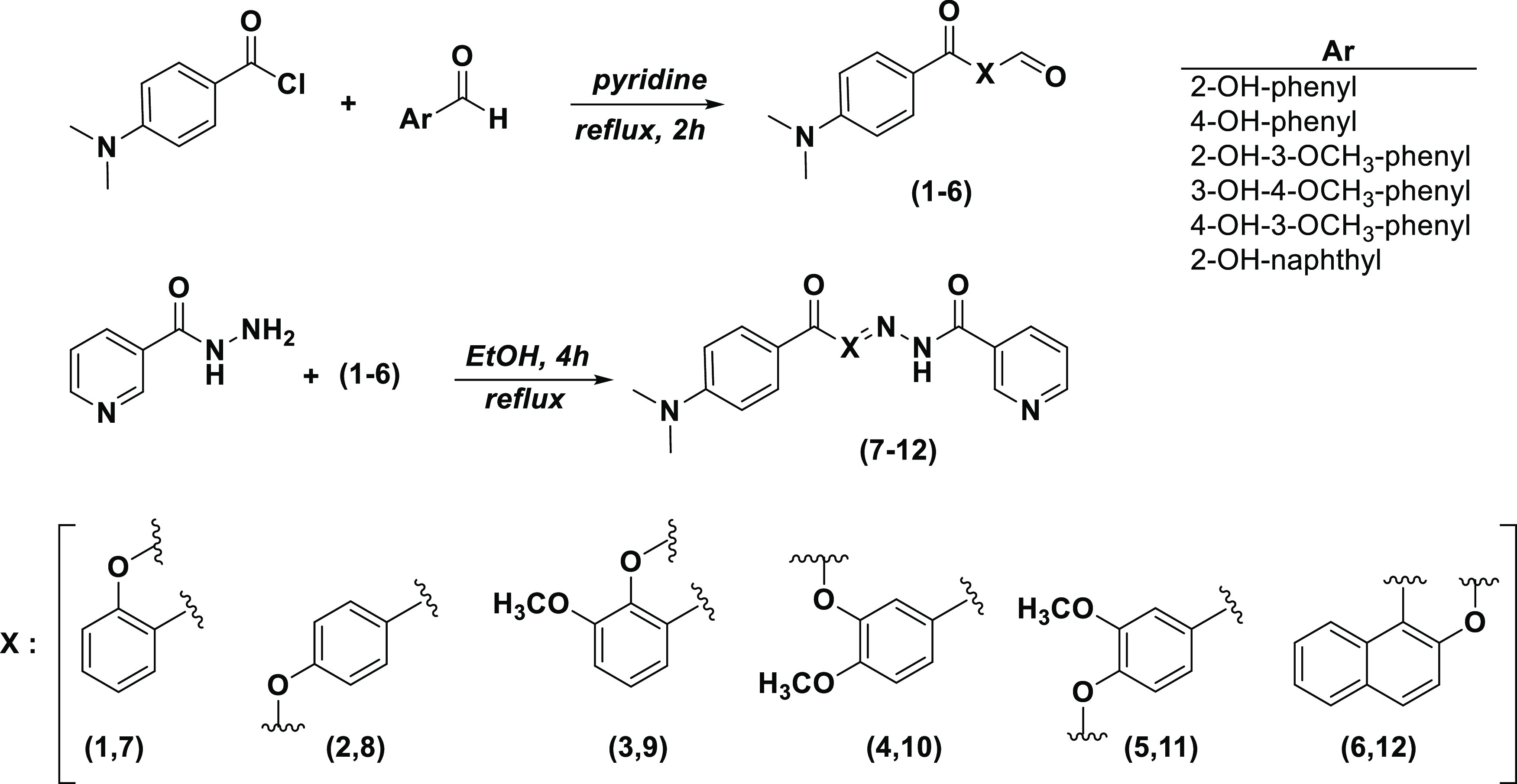
General Synthesis Procedure for Target Compounds

Small organic compounds serve as bioactive scaffolds, which are an important component of drug design. *N*-Acyl hydrazones are a significant member of the class of organic compounds used in drug design studies. In this study, new hydrazone compounds (**7**–**12**) as potential inhibitors of AChE, BChE, hCA I, and hCA II were prepared *via* the reaction of nicotinic hydrazide with aryl esters (**1**–**6**) obtained from 4-(dimethylamino)benzoyl chloride and aromatic aldehydes bearing substituted hydroxy and methoxy groups. The products were obtained in good yields (71–79%). The synthesized hydrazones were characterized by physical parameters (color and melting points). The increase in melting point of the products is the initial parameter to authenticate their formation. Further authentication was achieved with elemental analysis, FT-IR, ^1^H NMR, and ^13^C NMR techniques, and all spectra for synthesized compounds are provided in the Supporting Information. In this study, the synthetic strategy for the target molecules (**7**–**12**) is schematically outlined in Scheme 1. Synthesis of new hydrazone derivatives was carried out in two steps in good yields.^2,27,31^ First, aryl esters (**1**–**6**) were obtained by the reaction of six phenolic aldehydes (2-hydroxybenzaldehyde, 4-hydroxybenzaldehyde, 2-hydroxy-3-methoxybenzaldehyde, 3-hydroxy-4-methoxybenzaldehyde, 4-hydroxy-3-methoxybenzaldehyde, and 2-hydroxy-1-naphthaldehyde) with 4-(dimethylamino)benzoyl chloride at reflux temperature of pyridine for 2 h. Next, six new hydrazones (**7**–**12**) were acquired by the reaction of six aryl esters with nicotinic hydrazide under reflux with constant magnetic stirring in an ethanol medium for 4 h.

In the FT-IR spectra of the target compounds (**7**–**12**), the position of the NH stretching band within their molecular structure may undergo displacement, contingent upon the strength of both intra- and intermolecular hydrogen-bonding interactions. From this standpoint, it was observed that the NH stretching bands resided within the spectral region of 3186–3294 cm^–1^. The oscillations related to the stretching of CH bonds in the aromatic groups of the compounds in question were detected in the spectral region of 3007–3070 cm^–1^. The most salient feature exhibited by the hydrazone derivatives is the C=O stretching band of compounds **7**–**12**. The compounds exhibited highly distinct peaks in the range 1636–1685 cm^–1^, corresponding to the C=O stretching bands. The vibrational frequency of the C = N stretching band of the azomethine moiety within the structure was observed in the spectral range 1594–1608 cm^–1^.

In the ^1^H NMR spectrum of hydrazone compounds, the most prominent proton showing the formation of these structures was the CH=N proton of azomethine. The azomethine protons exhibited a distinctive singlet resonance signal in the range of 8.38–8.93 ppm. NH protons were observed as singlet within the range of 11.97–12.17 ppm. The protons with aromatic groups exhibited resonance in the chemical shift range of 8.77–8.88 ppm. In all of the hydrazones, the protons of the *N*-dimethyl group in the skeletal structure were found to resonate at approximately 3 ppm. In addition, the aliphatic protons in compounds **9**–**11**, which have a methoxy group, exhibited resonance as a singlet at 3.75–3.81 ppm.

Upon closer inspection of the ^13^C NMR spectra of the above-mentioned structures, signals belonging to the carbonyl carbon were determined in the range of 162.01–162.36 ppm. At the same time, the most characteristic peak in the elucidation of these structures is the peak of the azomethine carbon. The resonance peak of the CH=N carbons is detected at 149.00–149.11 ppm. Resonance signals within the range of 111.40–154.47 ppm were observed from the aromatic carbons. The methyl carbon of the *N*-dimethyl groups was observed at approximately 40 ppm. In compounds **9**–**11** containing a methoxy group, a carbon signal of approximately 56 ppm was observed. As a result, we determined that the characterization data were compatible with the structures of the targeted molecules.

### 3.2 Biological Activity Studies

Nowadays, there are many studies targeting AChE and BChE inhibitors in the therapy of some cognitive disorders, including AD. It is known that hCA inhibitors provide potential use in the treatment of glaucoma and many other diseases. Studies conducted in recent years have suggested that metabolic enzyme inhibitors have the potential to be used in the treatment of different diseases beyond their primary use in clinical applications. For example, CA inhibitors, such as AZA and MTZ, were first developed as diuretics and have recently begun to be investigated as potential therapeutics for AD. AZA and MTZ are both already FDA approved not only for hypertension but also for treatment of glaucoma, by reducing intraocular pressure and for high-altitude sickness, *via* reduction in pulmonary vasoconstriction, as well as through their ability to increase cerebral blood flow and reduce cerebral edema. It is asserted in recent studies on these inhibitors that CA inhibitors have the potential to be promising drugs to also treat neurovascular pathology associated with cerebral amyloid angiopathy and AD through their ability to prevent amyloid β-mediated mitochondrial dysfunction and cell death.^25,26^ As a result, based on the vast array of diseases that CA inhibitors are beneficial through multiple molecular mechanisms, which still remain to be elucidated.^51−53^ Therefore, investigating the potential of newly synthesized derivatives to inhibit both cholinesterase enzymes and CA isoenzymes is of great importance in terms of discovering multifunctional inhibitor candidates.

Today, many researchers are constantly searching for new and powerful inhibitors of these enzymes due to the inadequacy and unwanted side effects of existing drugs. In this study, 12 compounds were synthesized as potential inhibitors of these enzymes. Six of these were esters (**1**–**6**) and six were hydrazone derivatives (**7**–**12**) of these esters. The inhibition results of all molecules and reference drugs tested on these four metabolic enzymes are given in Table 1. These results demonstrated that all tested molecules were highly effective on these metabolic enzymes at nanomolar concentrations.

**Table 1:** IC_50_ Values of Tested Compounds against hCA I, hCA II, AChE, and BChE

	IC_50_ (nM)^a^			
compound	hCA I	hCA II	AChE	BChE
**1**	27.52 ± 0.51	13.74 ± 0.27	51.07 ± 0.88	43.54 ± 0.75
**2**	25.63 ± 0.47	12.15 ± 0.21	48.53 ± 0.81	41.47 ± 0.68
**3**	39.58 ± 0.72	16.13 ± 0.33	59.46 ± 0.96	51.27 ± 0.89
**4**	38.15 ± 0.65	15.42 ± 0.32	60.23 ± 1.02	53.14 ± 0.91
**5**	39.15 ± 0.71	20.36 ± 0.35	61.37 ± 1.12	54.74 ± 0.91
**6**	45.12 ± 0.82	24.74 ± 0.43	28.16 ± 0.52	25.17 ± 0.42
**7**	29.11 ± 0.53	8.03 ± 0.14	48.63 ± 0.79	45.27 ± 0.82
**8**	21.35 ± 0.39	7.12 ± 0.12	46.27 ± 0.75	43.38 ± 0.83
**9**	35.74 ± 0.74	12.46 ± 0.21	43.16 ± 0.81	39.56 ± 0.71
**10**	32.46 ± 0.73	11.84 ± 0.22	29.63 ± 0.53	29.72 ± 0.54
**11**	32.17 ± 0.72	11.56 ± 0.22	28.56 ± 0.52	28.56 ± 0.50
**12**	42.75 ± 0.81	23.45 ± 0.41	21.45 ± 0.38	18.42 ± 0.36
AZA	286.66 ± 2.42	26.63 ± 0.38		
neostigmine			135.90 ± 1.86	84.0 ± 1.07
rivastigmine			60.00 ± 0.73	14.10 ± 0.35

a Mean from at least three determinations.

All tested molecules in hCA I assay showed significant activity in the range of 21.35–45.12 nM. These molecules inhibited hCA I more potently than AZA, as seen in Table 1. Compared to AZA, ester (**1**–**6**) and their hydrazone derivatives (**7**–**12**) showed similar activity. Except for compound **7**, hydrazone derivatives (**8**–**12**) inhibited this enzyme slightly better than their ester derivatives. Among these compounds, compound **8** (21.35 nM), hydrazone derivative based on 4-hydroxybenzaldehyde, was the most active inhibitor and inhibited hCA I enzyme 13-fold more than AZA (286.66 nM). This compound (7.12 nM) was also the most active inhibitor against hCA II compared to AZA (26.63 nM). In hCA II assay, all molecules tested exhibited inhibitory activity against hCA II in different nanomolar ranges (7.12–24.74 nM). In addition to compound **8** in the series, all other compounds tested inhibited hCA II better compared to AZA.

Moreover, the inhibition activities of these molecules on AChE and BChE in this study were determined according to the Ellman method. As can be seen in Table 1, these molecules (**1**–**12**) were determined to have a higher potential to inhibit AChE and BChE compared to that of neostigmine. Compound **12**, the bulkiest molecule in the series, was the most active inhibitor against these enzymes. All screened molecules in the AChE assay exhibited activity with IC_50_ values ranging from 21.45 to 61.37 nM against AChE. In the AChE assay, the esters and their hydrazone derivatives demonstrated similar activity against this enzyme. Among them, compound 1**2** (21.45 nM), a 2-hydroxy-1-naphthaldehyde-based hydrazone compound, inhibited AChE approximately 3-fold compared with rivastigmine (60.0 nM) and more than 6-fold compared to neostigmine. In addition, this compound (18.42 nM) was the most active inhibitor against BChE, inhibiting approximately 5-fold more than neostigmine (84.0 nM) and showing activity close to rivastigmine (14.10 nM). All molecules tested in this assay showed inhibitory activities against BChE at nanomolar concentrations ranging from 18.42 to 54.74 nM. Apart from these, compound **5**, an ester derivative based on 4-hydroxy-3-methoxybenzaldehyde, also displayed weaker activity against AChE compared to neostigmine and rivastigmine. On the other hand, the same compound inhibited BChE better than neostigmine.

### 3.3 *In Silico* Studies

In *in silico* approach to evaluate 12 chemicals as inhibitors of the AChE, BChE, hCA I, and hCA II target proteins, 12 compounds were converted into three-dimensional (3D) low-energy structures, including the reference compounds neostigmine (for AChE and BChE) and AZA (for hCA I and hCA II). Glide/SP, IFD, and QPLD were employed in docking to determine the various ligand binding poses of the compounds. For additional structural and dynamic investigations, top-docking poses of compounds at the binding pockets were investigated. Based on biological activity results, hit compound **12** was identified as promising for AChE and BChE and **8** was the most active molecule for hCA I and hCA II. Thus, these promising hit compounds were used in MD simulations for better understanding of their biological actions at the targeted structures. For this purpose, 200 ns MD simulations of their top-IFD docking poses at the AChE, BChE, hCA I, and hCA II targets were performed. The change in binding free energies (*i.e.*, MM/GBSA) over time was evaluated for the selected compounds as well as the reference drugs neostigmine and AZA. The results of the molecular docking and MD simulations are summarized in Tables 2–5. Table 2 shows docking scores of the studied 12 compounds and reference ligand at the AChE and BChE targets.

**Table 2:** Docking Scores in Glide/SP, IFD, and QPLD of Studied and Reference Compounds against AChE and BChE

	AChE (kcal/mol)			BChE (kcal/mol)		
compounds	SP	IFD	QPLD	SP	IFD	QPLD
**1**	–9.06	–10.17	–8.85	–8.15	–9.47	–6.19
**2**	–8.00	–10.35	–7.42	–7.37	–8.76	–6.86
**3**	–9.51	–11.98	–8.69	–8.36	–10.10	–6.32
**4**	–9.25	–10.85	–7.89	–8.35	–7.85	–7.29
**5**	–9.57	–10.29	–7.09	–4.91	–7.39	–6.29
**6**	–10.55	–10.71	–8.33	–7.39	–9.08	–7.03
**7**	–10.18	–12.18	–10.39	–9.19	–10.28	–8.43
**8**	–9.51	–13.58	–7.51	–6.40	–10.81	–6.26
**9**	–10.15	–11.72	–4.81	–9.17	–10.80	–8.25
**10**	–9.64	–12.81	–8.22	–9.39	–10.40	–7.36
**11**	–9.87	–11.32	–7.86	–6.39	–9.48	–6.54
**12**	–11.33	–13.75	–9.01	–10.05	–10.39	–9.89
neostigmine	–7.36	–11.42	–10.26	–5.67	–6.65	–6.17

MD simulations constitute a crucial *in silico* approach for investigating ligand-induced conformational changes and temporal fluctuations in protein structures. By introducing atomic-level perturbations, MD simulations facilitate a detailed exploration of the dynamic behavior of proteins and their interactions with ligands. This method provides valuable insights into the structural dynamics, flexibility, and conformational transitions of proteins, offering a deeper understanding of how ligands impact protein structure and function. MD simulations are particularly advantageous, because they consider the inherent flexibility and dynamic nature of macromolecules. This characteristic makes MD simulations more comparable to the biologically relevant systems in cellular physiological conditions, in contrast to the molecular docking approach. MD simulations contribute significantly to unraveling the intricate details of protein–ligand interactions, providing a more realistic depiction of their behavior over time.

To explore the behavior of the identified promising hit compounds within the active site of AChE, BChE, hCA I, and hCA II, extensive all-atom MD simulations were conducted for a duration of 200 ns in an explicit solvent environment, taking into account the surrounding water molecules for a more accurate representation of the physiological conditions. By performing these simulations, we aimed to gain insights into the dynamics, stability, and interactions of the compound AChE, BChE, hCA I, and hCA II complexes over an extended time period.

In our investigation, we utilized the MM/GBSA approach. This computational methodology enabled us to predict the binding free energy of the protein–ligand complex under scrutiny. By integrating molecular mechanics, which account for atomic interactions within the complex, with the generalized Born (GB) implicit solvent model and solvent-accessible surface area (SA) calculations, we estimated the contributions of solvation and intermolecular interactions to the predicted binding affinity. The MM/GBSA approach provided valuable insights into the energetics of the protein–ligand interaction, allowing us to evaluate the stability and potential binding strength of the complex. This comprehensive analysis sheds light on the underlying factors influencing the binding free energy, enhancing our understanding of the thermodynamics governing the protein–ligand complex in our study. Table 3 shows the average MM/GBSA score of compound **12** and reference ligand neostigmine at the binding pockets of AChE and BChE.

**Table 3:** Average MM/GBSA Scores of Identified Hit Compound **12** and Reference Compound Neostigmine against AChE and BChE

compound	average MM/GBSA (kcal/mol) AChE	average MM/GBSA (kcal/mol) BChE
**12**	–80.56 ± 8.46	–76.89 ± 12.79
neostigmine	–40.33 ± 4.71	–49.63 ± 5.54

Table 4 represents the docking scores of the studied 12 compounds and reference compound AZA at the hCA I and hCA II targets.

**Table 4:** Docking Scores in Glide/SP, IFD, and QPLD of Studied and Reference Compounds against hCA I and hCA II

	hCA I (kcal/mol)			hCA II (kcal/mol)		
compounds	SP	IFD	QPLD	SP	IFD	QPLD
**1**	–6.48	–7.05	–5.03	–5.82	–6.19	–4.61
**2**	–6.15	–6.21	–4.91	–4.77	–6.06	–5.44
**3**	–6.66	–7.84	–4.18	–6.13	–8.54	–4.36
**4**	–6.18	–7.38	–4.77	–6.21	–8.01	–4.88
**5**	–7.42	–6.50	–4.79	–5.87	–8.38	–5.25
**6**	–6.75	–8.72	–4.59	–6.51	–10.59	–6.27
**7**	–6.52	–7.86	–4.81	–5.47	–9.08	–4.80
**8**	–4.11	–6.75	–4.84	–4.33	–6.03	–4.14
**9**	–6.46	–8.96	–5.82	–5.26	–8.39	–4.73
**10**	–5.96	–6.80	–5.06	–6.40	–7.26	–3.49
**11**	–5.48	–5.49	–4.86	–6.05	–9.95	–4.73
**12**	–6.51	–8.57	–4.73	–6.61	–9.02	–5.24
AZA	–4.59	–3.34	–2.86	–5.92	–4.58	–4.72

Compound **8** is selected for the MD simulations, and its results were compared with the AZA. Table 5 represents the average binding free energy results of **8** and AZA.

**Table 5:** Average MM/GBSA Scores of Identified Hit Compound **8** and Reference against hCA I and hCA II

compound	average MM/GBSA (kcal/mol) hCA I	average MM/GBSA (kcal/mol) hCA II
**8**	–34.55 ± 4.71	–37.65 ± 8.73
AZA	–10.83 ± 6.66	–16.82 ± 4.14

As depicted in the tables, compound **12** exhibited strong predicted potency against AChE and BChE, as indicated by their negative binding free energy values (Δ*G*_bind_). The calculated Δ*G*_bind_ values of **12** for AChE and BChE were −80.56 and −76.89 kcal/mol, respectively, suggesting favorable and energetically favorable interactions between the compounds and the target proteins. The average binding free energies of compound **8** at hCA I and hCA II were calculated as −34.55 and −37.65 kcal/mol, respectively. These average binding free energy values are better than the calculated binding free energies of the corresponding reference compound (AZA) against hCA I and hCA II, which have −10.83 and −16.82 kcal/mol, respectively.

By elucidating the three-dimensional structure of protein–ligand complexes, valuable insights can be obtained regarding the precise interactions between the ligand and the protein. This knowledge can be leveraged to design novel ligands with enhanced binding affinities. Ultimately, the 3D poses of protein–ligand complexes provide a structural foundation for comprehending the molecular interactions underlying ligand–receptor binding, enabling the development of ligands with superior binding affinities.

**Figure 2:**
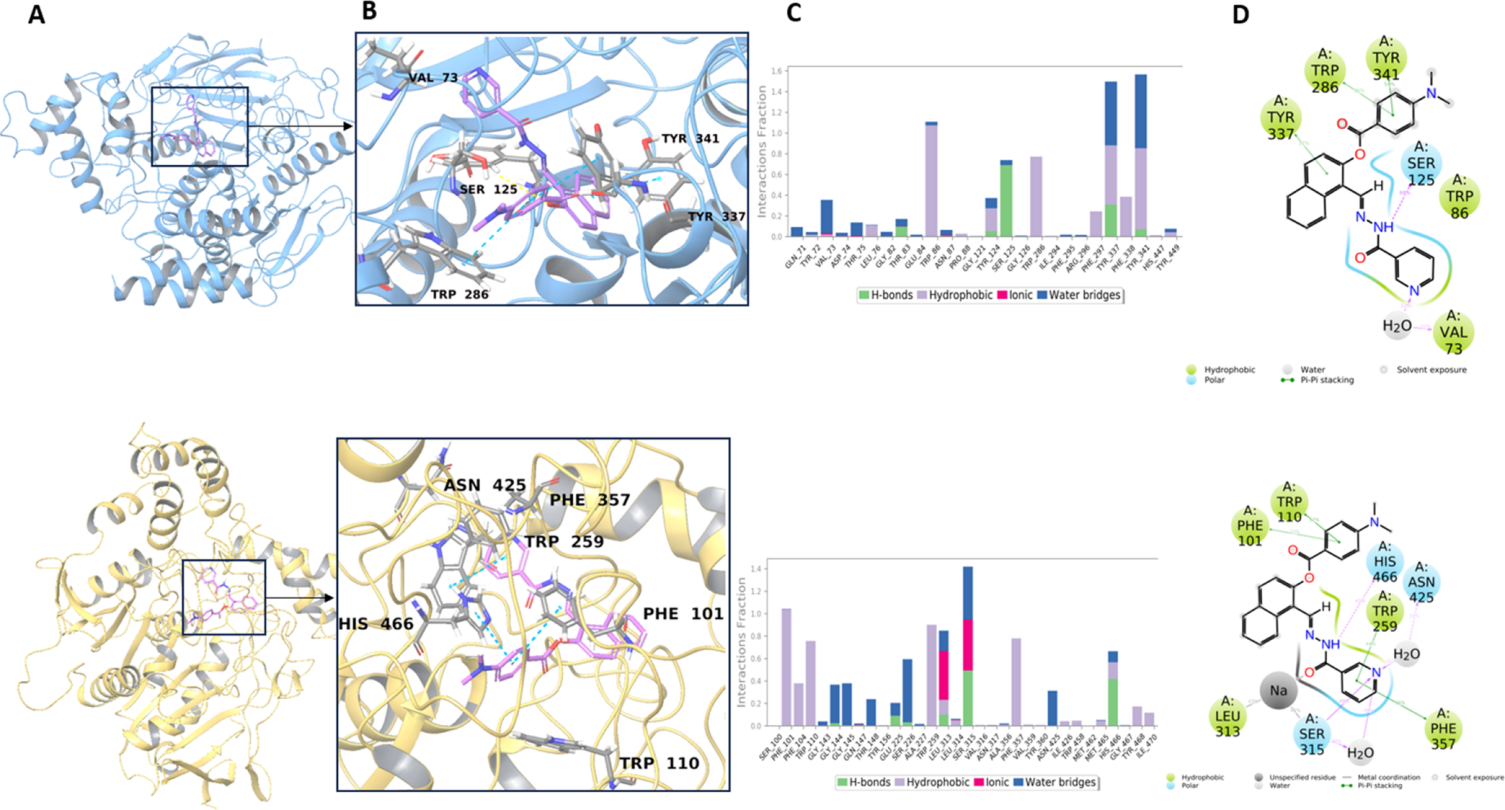
(A) Representative structures of compound **12** (top, AChE; bottom, BChE) complexes at the binding site. The protein structures are displayed in ribbon representation. (B) Zoomed views, ligand molecules, and interacting residues are shown in stick representation. The representative structures were extracted from the concatenated MD simulation trajectories by selecting the conformations with the smallest root-mean-square deviation (RMSD). (C) Interaction fractions (top, AChE; bottom, BChE) with binding pocket residues for the MD simulations initiated by the IFD docking poses. Bar charts show hydrogen bonds (green), hydrophobic interactions (purple), ionic interactions (red), and water bridges (blue). (D) Detailed 2D ligand atom interactions of the studied hit ligands (top, AChE; bottom, BChE) with the protein residues. Interactions that are maintained for more than 15.0% of the simulation time are represented.

**Figure 3:**
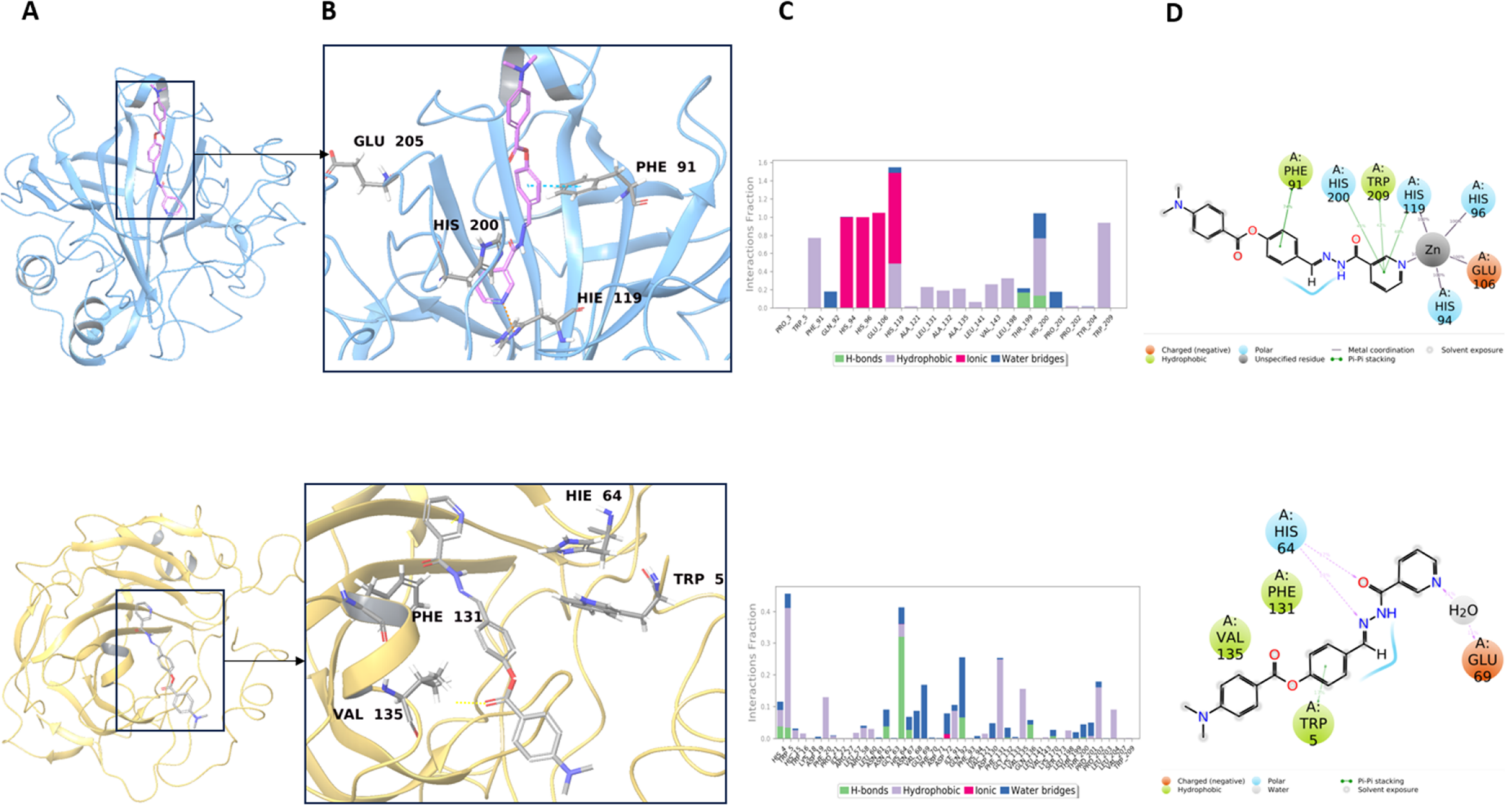
(A) Representative structures of compound **8** (top, hCA I; bottom, hCA II) complexes at the binding site. The protein structures are displayed in ribbon representation. (B) Zoomed views, ligand molecules, and interacting residues are shown in stick representation. The representative structures were extracted from the concatenated MD simulation trajectories by selecting the conformations with the smallest RMSD. (C) Interaction fractions (top, hCA I; bottom, hCA II) with binding pocket residues for the MD simulations initiated by the IFD docking poses. Bar charts show hydrogen bonds (green), hydrophobic (purple), ionic interactions (red), and water bridges (blue). (D) Detailed 2D ligand atom interactions of the studied hit ligands (top, hCA I; bottom, hCA II) with the protein residues. Interactions that are maintained more than 15.0% of the simulation time are represented.

Figures 2 and 3 represent information regarding the 3D binding modes and 2D ligand interaction graphs of compound **12** at the AChE and BChE and of compound **8** at hCA I and hCA II, respectively. Compound **12** displayed critical hydrogen-bonding interactions with Val73, Ser125, and Tyr337 residues of AChE, which remained largely conserved throughout the MD simulations. These findings strongly indicate the significance of these hydrogen-bond interactions for the binding affinity of compound **12** to AChE. Hydrogen bonds are of crucial significance in protein–ligand binding as they aid in ligand orientation within the binding pocket and significantly contribute to the overall stability of the complex. Nonpolar interactions play a significant role in stabilizing protein–ligand complexes within hydrophobic environments. Compound **12** establishes hydrophobic interactions with key amino acid residues, including Trp286 and Tyr341, enhancing the ligand’s overall stability within the binding site of AChE. Trp286, Tyr341, and Tyr337 form π–π stacking interactions with the aromatic rings of compound **12**. Compound **12** mainly forms hydrophobic and hydrogen-bonding interactions with BChE. While Phe101, Trp110, Trp259, and Phe357 form π–π stacking interactions with the ligand from terminal aromatic rings, His466 constructs a hydrogen-bonding interaction. Ser315 and Asn425 form water-bridged hydrogen-bonding interactions with compound **12** (Figure 2).

Crucial interactions from the binding pockets of hCA I with compound **8** are observed with Phe91, Trp209, His119, and His200. Corresponding interactions were formed by Val135, Trp5, Phe131, Hi64, and Glu69 residues at the hCA II (Figure 3).

We also utilized computational methods to predict the bioavailability of the synthesized compounds. Specifically, we employed *in silico* tools (*i.e.*, SwissADME, http://www.swissadme.ch/) to assess parameters that evaluate key physicochemical and ADME properties associated with oral bioavailability. By integrating these computational predictions into our study, we gained valuable insights into the potential bioavailability and pharmacokinetic profiles of the synthesized compounds (Figures S1–S12).

Additionally, we used top-active compounds in cholinesterase (compound **12**) and carbonic anhydrase (compound **8**) and explored the synthesized analogues of these compounds which may be available at the small-molecule libraries. For this aim, we used SwissSimilarity server (http://www.swisssimilarity.ch/), checked the analogues of compounds **8** and **12**, and used structurally similar compounds (*i.e.*, Tanimato Coefficient >0.5) in molecular docking (IFD). The comprehensive search yielded a total of around 300 structurally similar compounds of compounds **8** and **12** characterized by a Tanimoto coefficient exceeding 0.5. Tables S1 and S3 show top-docking-scored analogues of **12** at the binding pocket of AChE and BChE, respectively. Top-scored analogues of **12** were used in 200 ns MD simulations and average MM/GBSA scores were compared (Tables S2 and S4). Similarly, analogues of **8** were docked in hCA I and II binding pockets (Tables S5 and S7). Top-scored analogues of **8** were used in 200 ns MD simulations, and average binding free energy analyses were compared. Binding free energy prediction results of analogues of **8** and **12** also represented promising results.

## 4 Conclusions

In summary, this study represents a pioneering investigation into the inhibition and computational analysis of four key metabolic enzymes using a series of aromatic esters (**1**–**6**) and their respective hydrazone derivatives (**7**–**12**). The elucidation of the chemical structures for the newly synthesized compounds was conducted with a comprehensive approach employing three spectroscopic techniques alongside elemental analysis.

The study revealed that hydrazone derivatives of these esters containing a dimethylamine moiety, such as rivastigmine used in the treatment of AD, exhibited remarkable inhibitory activity against targeted enzymes even at nanomolar concentrations.

The comprehensive analysis of the tested molecules (**1**–**12**) showed their superior inhibitory effects on hCA I, hCA II, AChE, and BChE compared with standard drugs. Notably, compounds **7** and **8** demonstrated remarkable inhibition on hCA I and II when compared to the reference drug AZA. Among these, compound **8** stood out for its exceptional activity against hCA II, surpassing the effectiveness of AZA. Furthermore, compound **8** exhibited significant inhibitory effects against hCA I.

Compound **12** emerged as a standout inhibitor, showcasing the highest potency against both AChE and BChE, outperforming neostigmine. The inhibitory effect on AChE by compound **12** was more than 6-fold higher than the reference drug, highlighting its potential therapeutic significance. Similarly, the inhibition of BChE by compound **12** was approximately 4 times greater than that of neostigmine. On the other hand, it was determined that all hydrazone derivatives inhibited AChE better than the standard molecule rivastigmine.

These findings provide valuable insights into the potential of the synthesized compounds, suggesting their promise as candidates for the design and synthesis of novel and potent inhibitors targeting human CAs. The study underscores the significance of these compounds in the realm of drug discovery for neurodegenerative disorders like AD.

## Supporting Information Available

Molecular characterization spectra (FT-IR, ^1^H NMR, and ^13^C NMR) of synthesized compounds **1**–**12**; IC_50_ plots of studied compounds and reference compounds against hCA I, hCA II, AChE, and BChE; 2D structures of top-docking scored compounds of analogues of **12** at the binding pocket of AChE and BChE; average MM/GBSA score of **12** and its top-scored analogues at the binding site of AChE and BChE; 2D structures of top-docking scored compounds of analogues of **8** at the binding pocket of hCA I and hCA II; average MM/GBSA scores of **8** and its top-scored analogues at the binding site of hCA I and hCA II; and ADME and physicochemical properties of the studied compounds **1**–**12** (PDF)
