# Supplementary material for: Synthesis of Novel Hydrazide–Hydrazone Compounds and In Vitro and In Silico Investigation of Their Biological Activities against AChE, BChE, and hCA I and II
Source: ACS Omega. 2024 Apr 26;9(18):20030–41. doi: 10.1021/acsomega.3c10182 (PMC11079868; doi:10.1021/acsomega.3c10182)
Supplement: Supplementary file 1 — ao3c10182_si_001.pdf [file ao3c10182_si_001.pdf]

Supporting Information

Synthesis of novel hydrazide-hydrazone compounds and *in vitro* and *in silico* investigation of their biological activities against AChE, BChE, hCA I and II

Reşit Çakmak [a,*], Eyüp Başaran[b], Kader Sahin[c], Murat Şentürk[d], Serdar Durdağı,[e,f,g,*]

[a]Medical Laboratory Techniques Program, Vocational School of Health Services, Batman University, Batman, Türkiye

[b]Department of Chemistry and Chemical Processing Technologies, Vocational School of Technical Sciences, Batman University, Batman, Türkiye

[c]Department of Analytical Chemistry, School of Pharmacy, Bahcesehir University, Istanbul, Türkiye.

[d]Department of Biochemistry, Pharmacy Faculty, Ağrı Ibrahim Çecen University, Ağrı, Türkiye

[e]Computational Biology and Molecular Simulations Laboratory, Department of Biophysics, School of Medicine, Bahçeşehir University, İstanbul, Türkiye

[f]Lab for Innovative Drugs (Lab4IND), Computational Drug Design Center (HITMER), Bahçeşehir University, İstanbul, Türkiye

[g]Molecular Therapy Lab, Department of Pharmaceutical Chemistry, School of Pharmacy, Bahçeşehir University, Istanbul, Türkiye

E-mails: resit.cakmak@batman.edu.tr (RÇ), and serdar.durdagi@bau.edu.tr (SD)

FT-IR spectrum of compound **1**

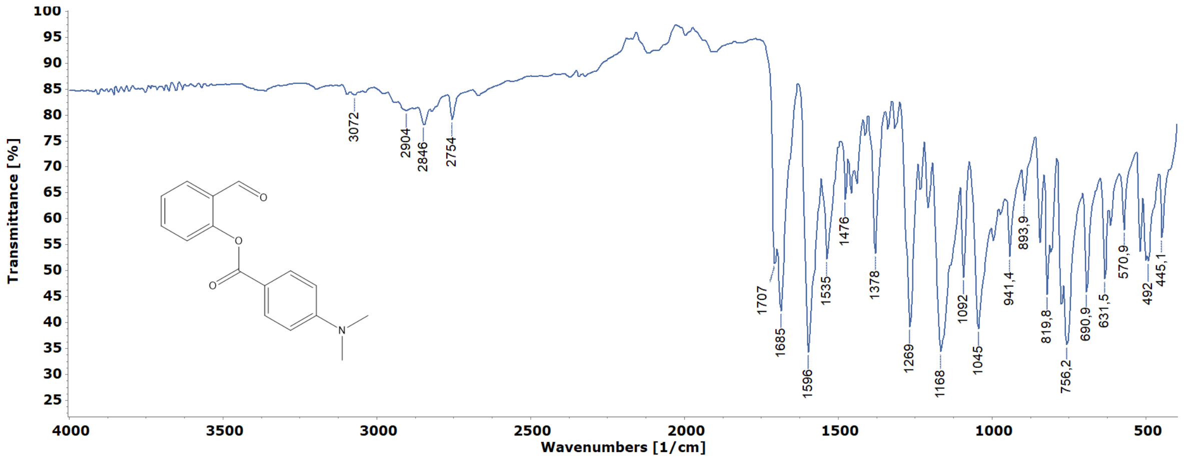

^{1}H NMR spectrum of compound **1**

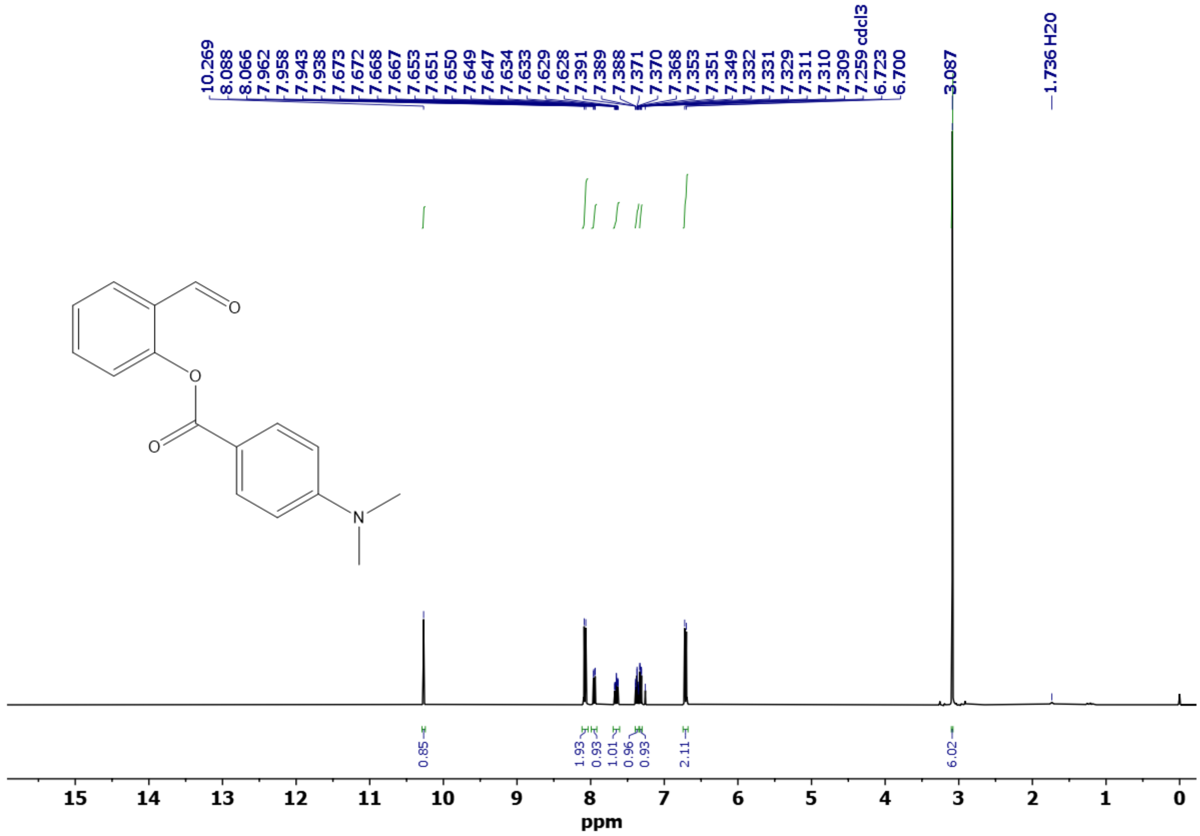

^{13}C NMR spectrum of compound **1**

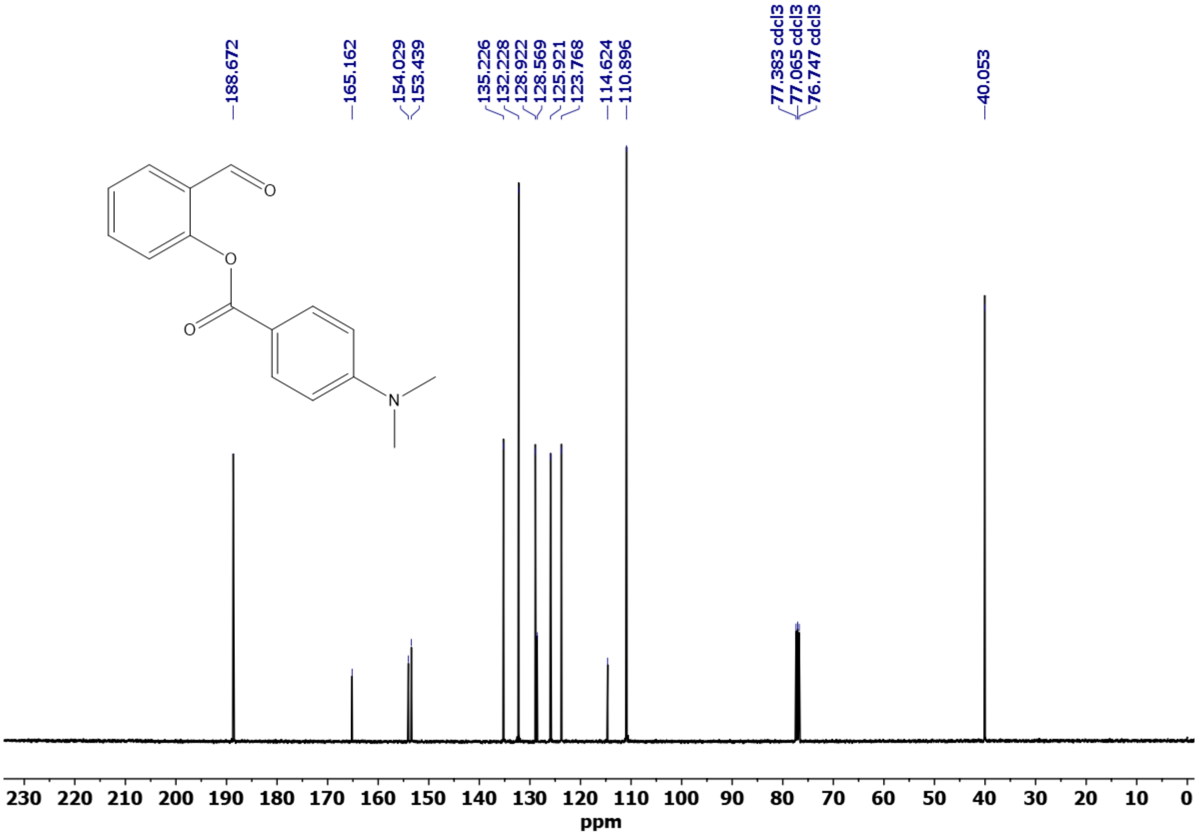

FT-IR spectrum of compound **2**

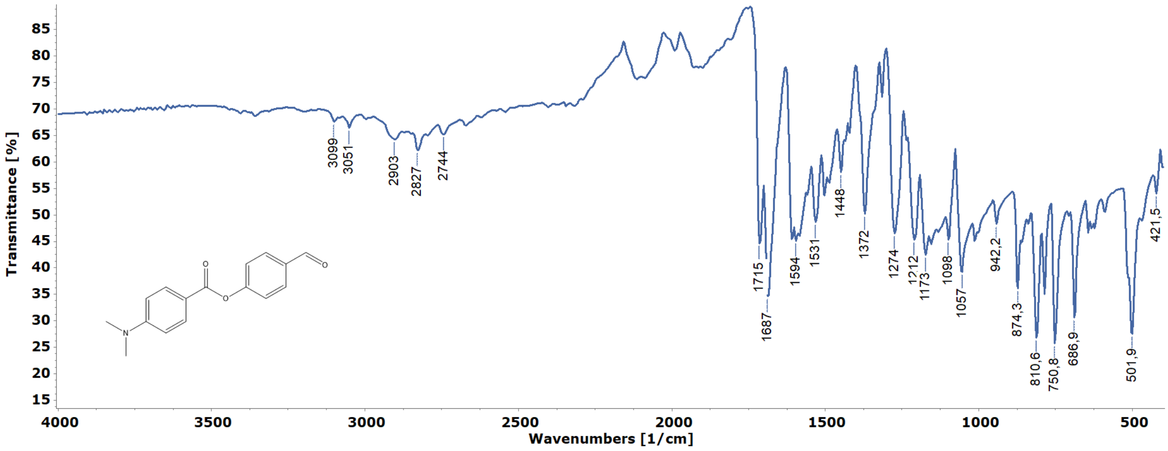

^{1}H NMR spectrum of compound **2**

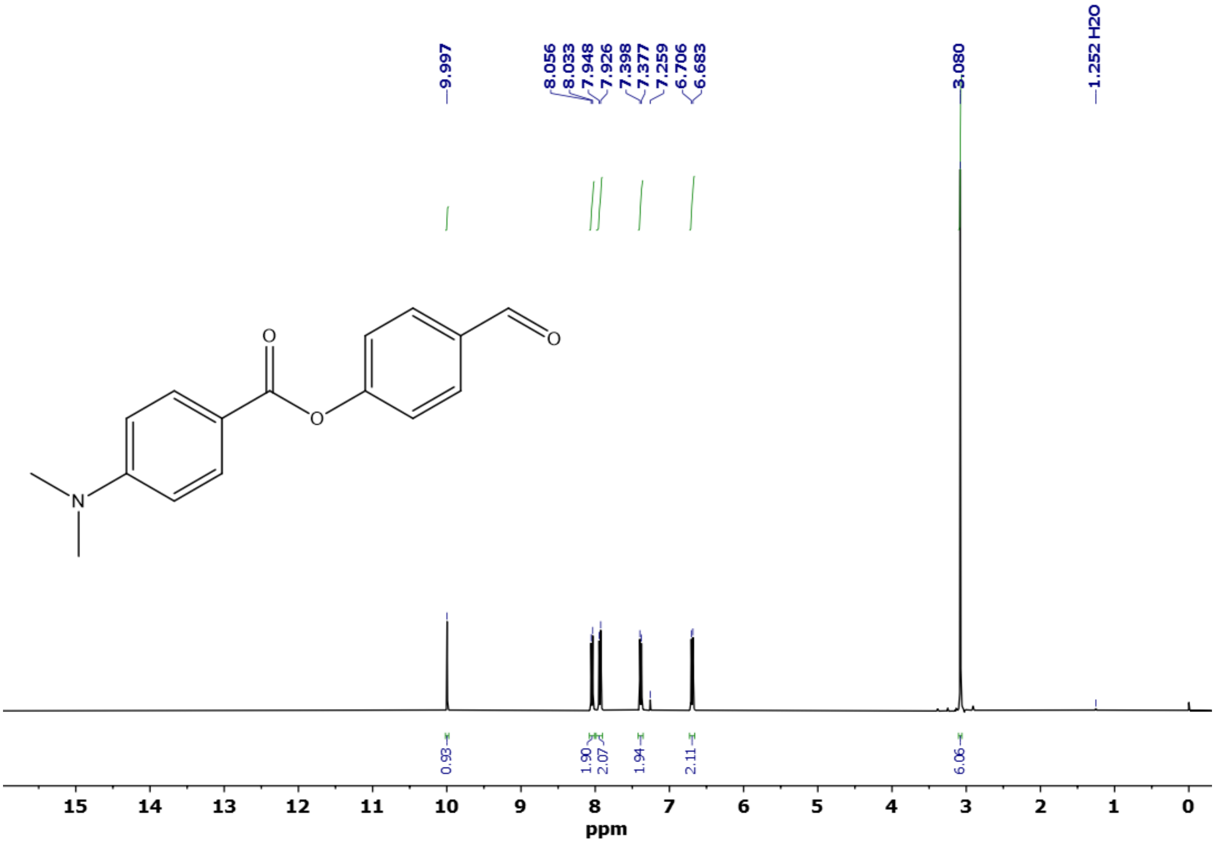

^{13}C NMR spectrum of compound **2**

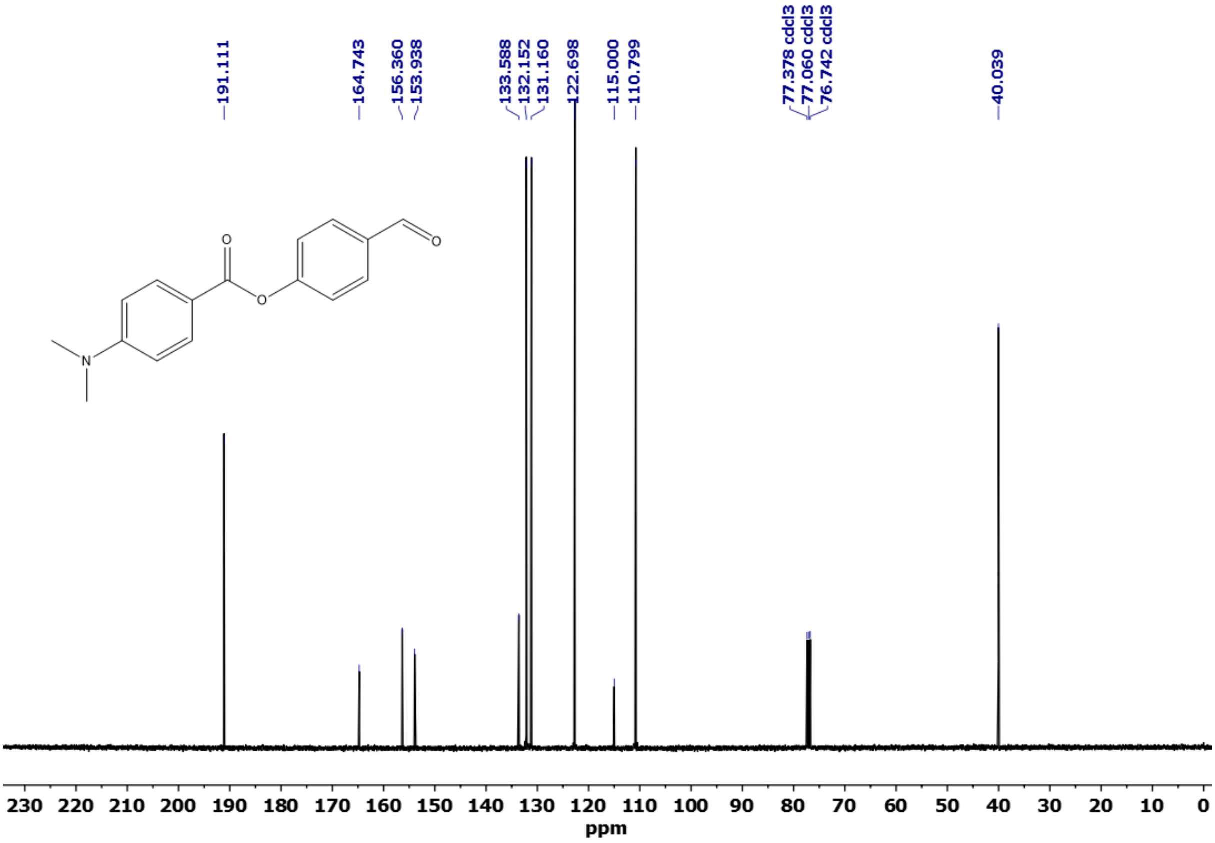

FT-IR spectrum of compound **3**

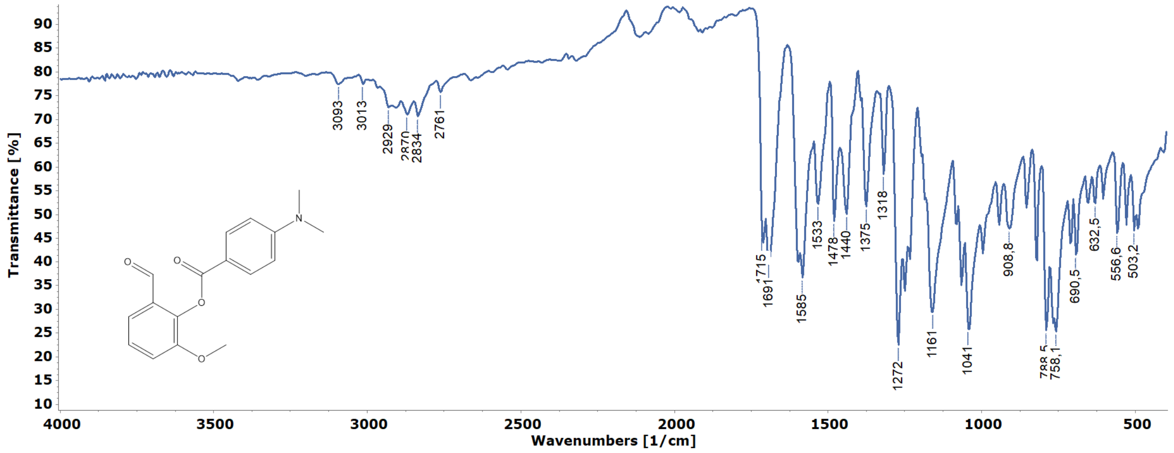

^{1}H NMR spectrum of compound **3**

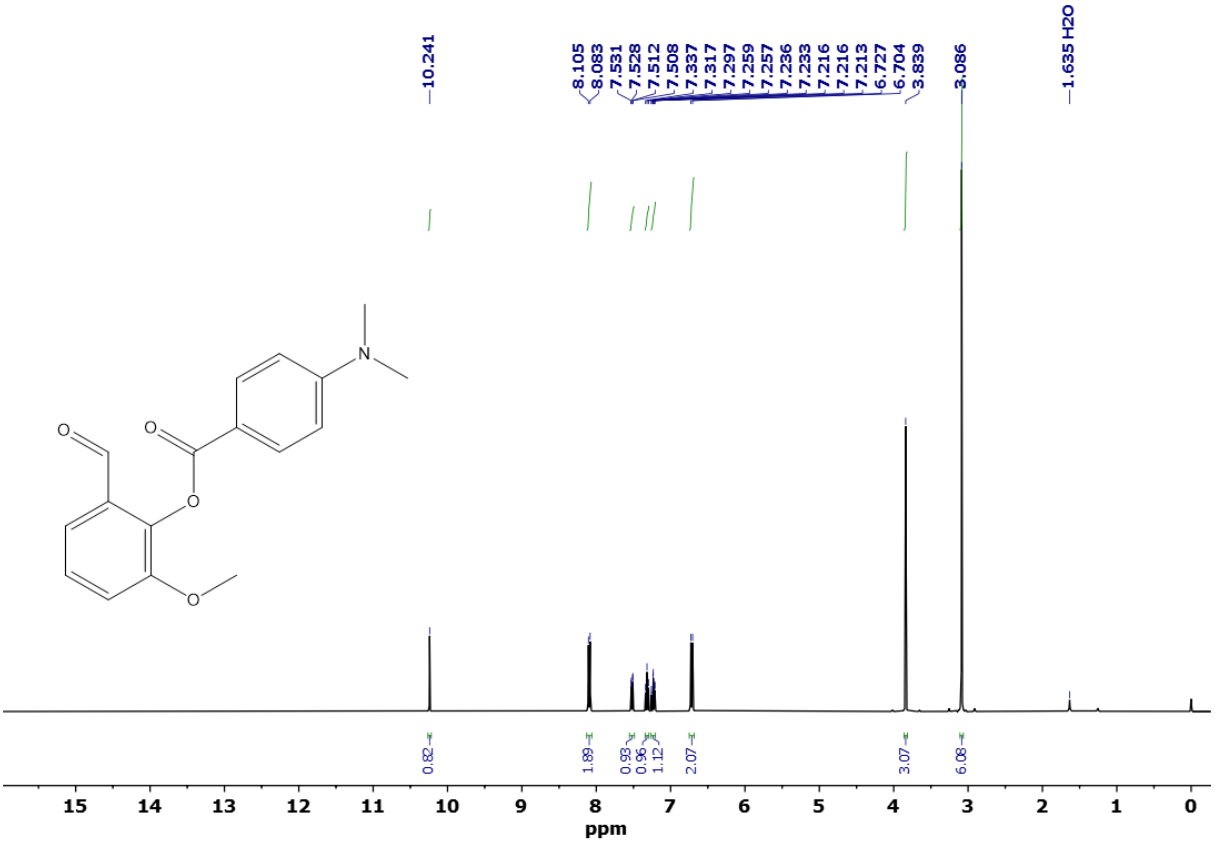

^{13}C NMR spectrum of compound **3**

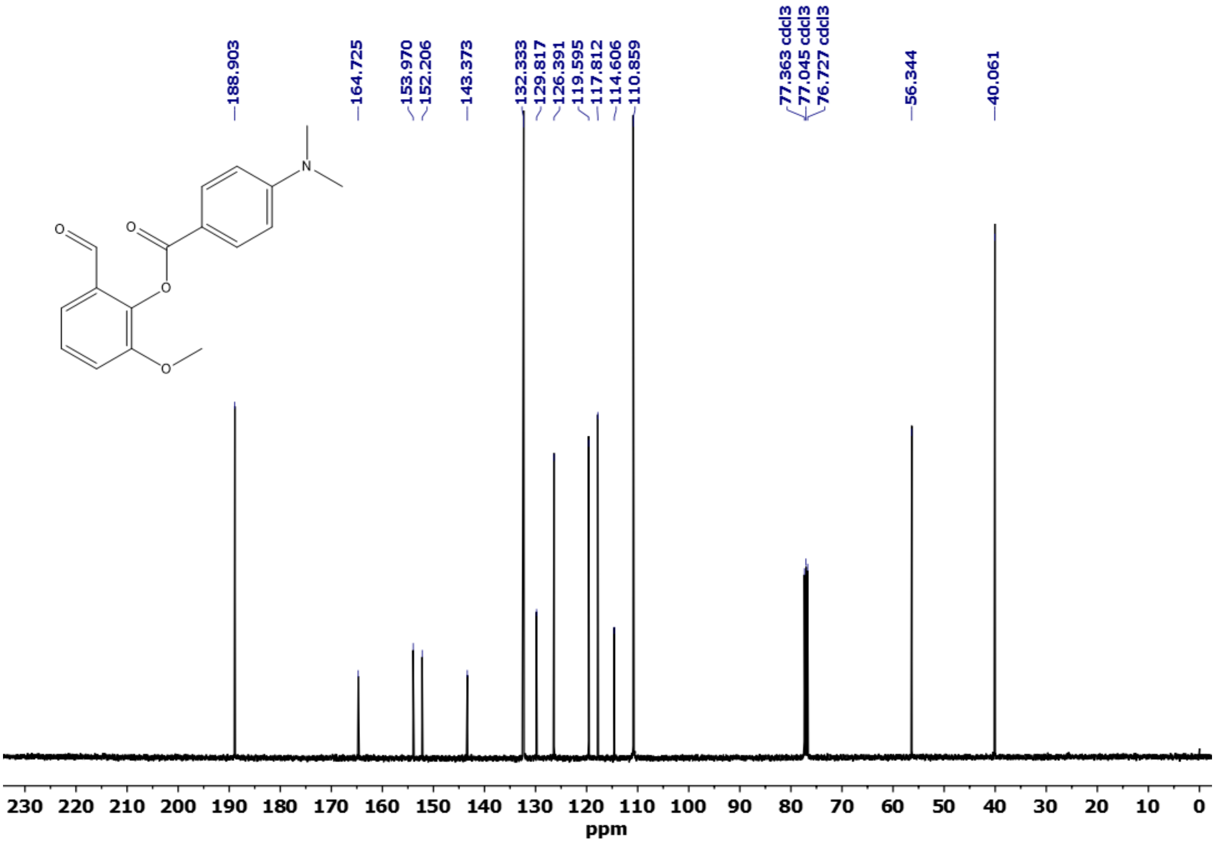

FT-IR spectrum of compound **4**

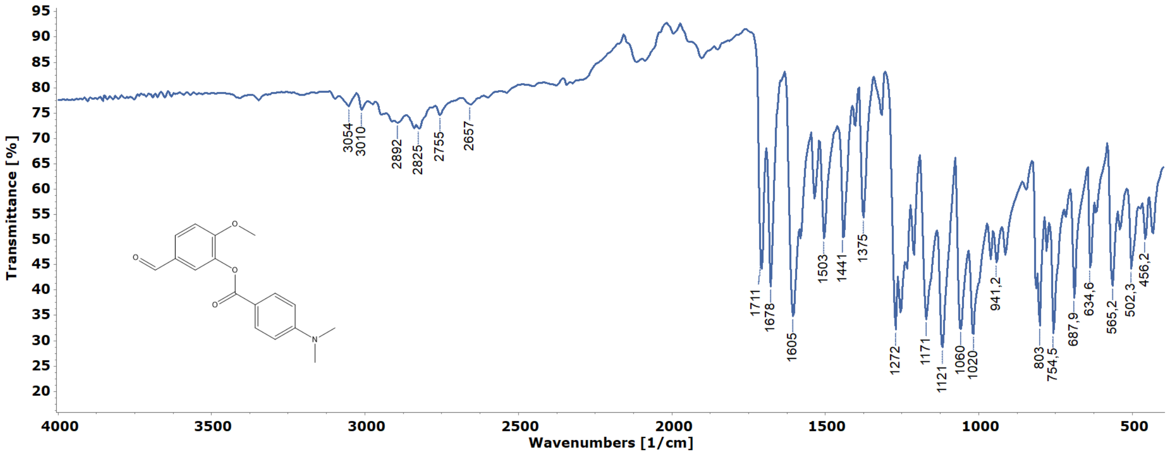

^{1}H NMR spectrum of compound **4**

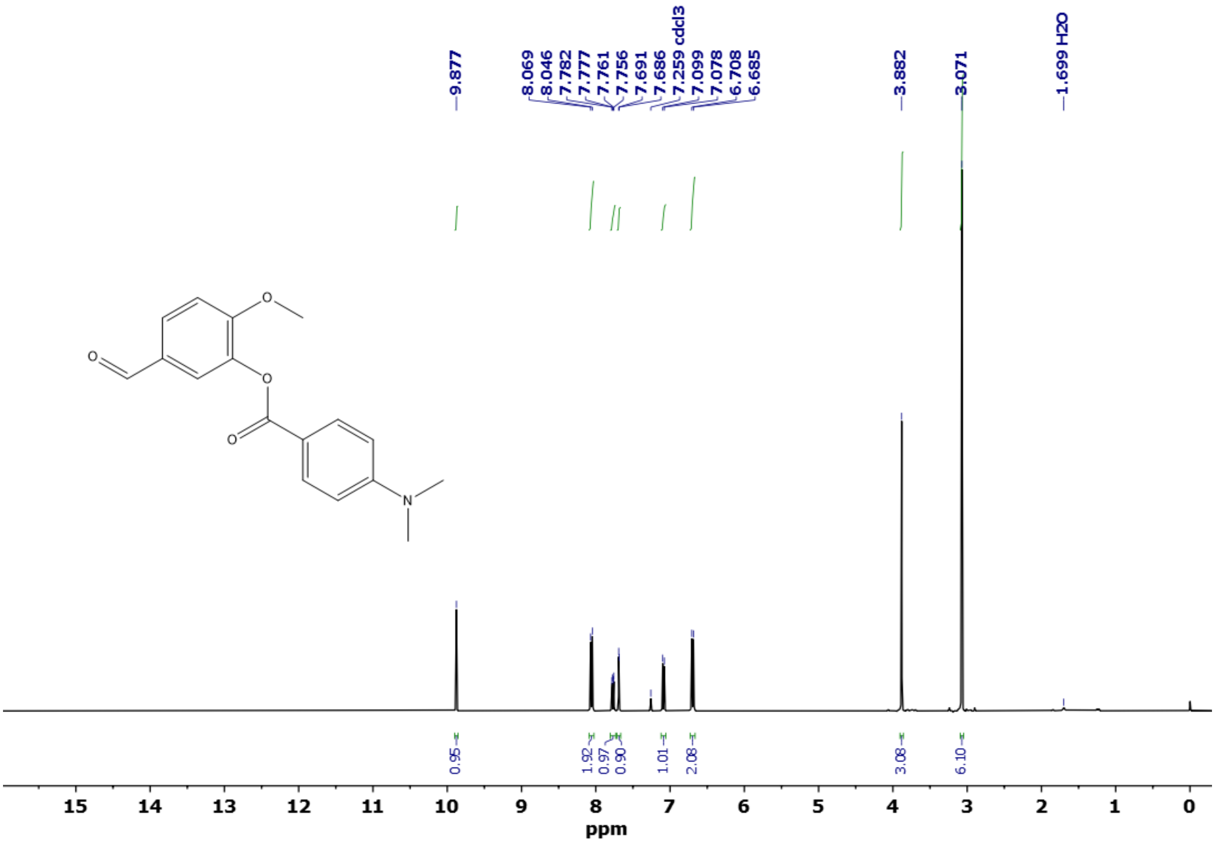

^{13}C NMR spectrum of compound **4**

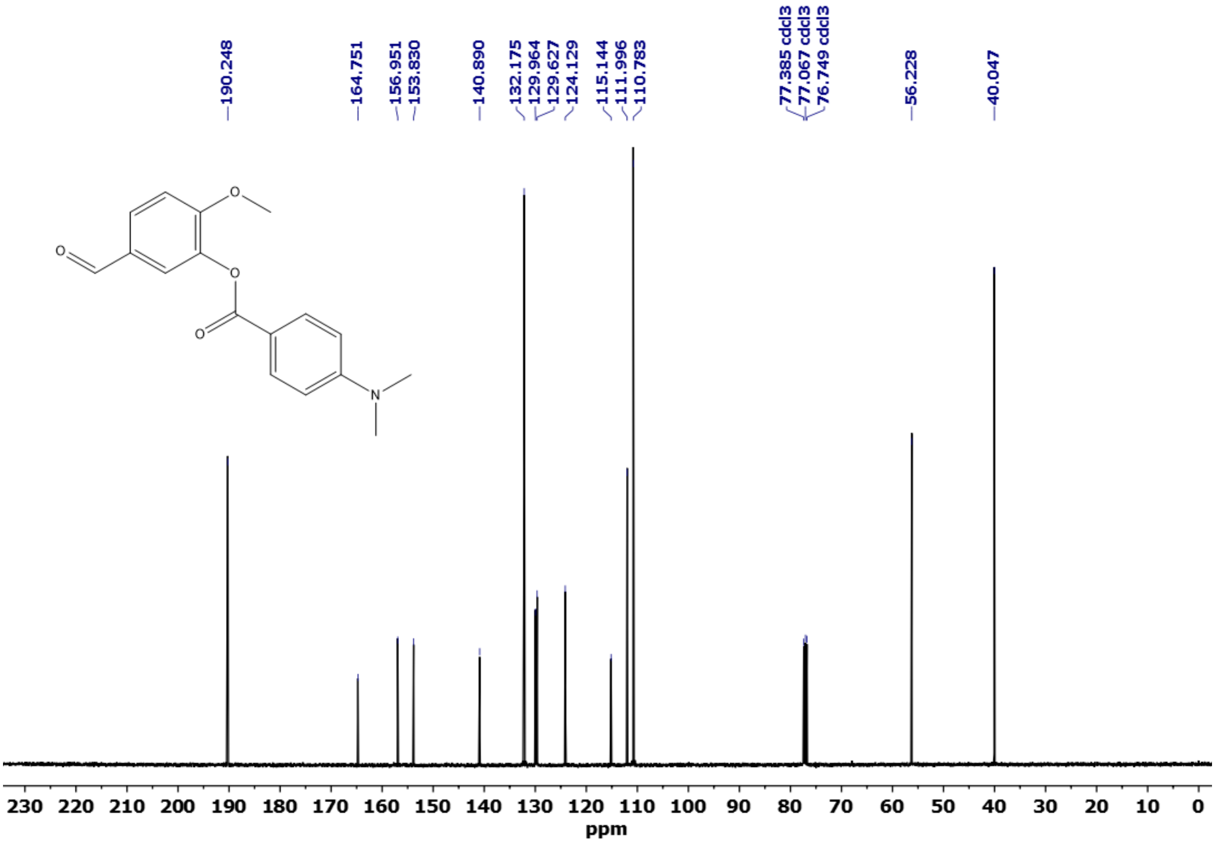

FT-IR spectrum of compound **5**

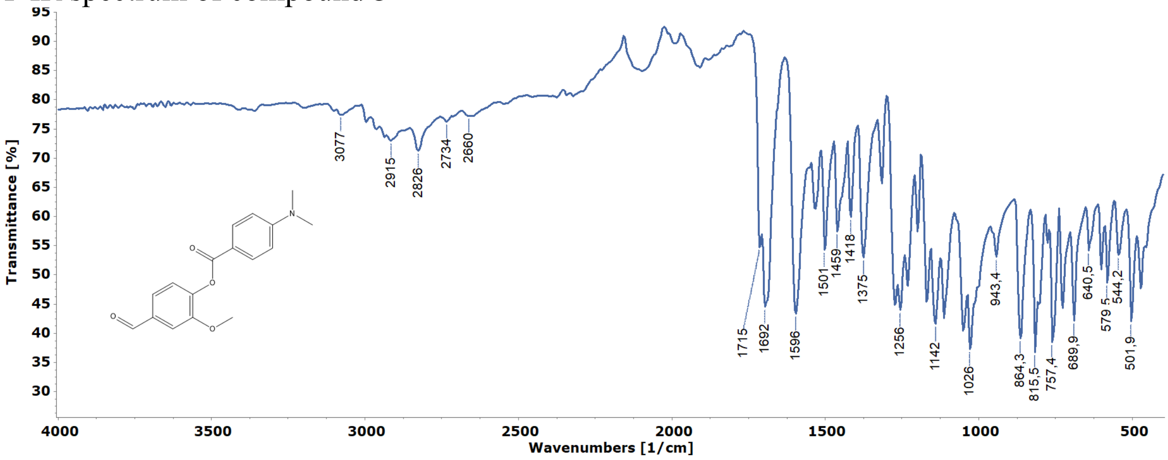

^{1}H NMR spectrum of compound **5**

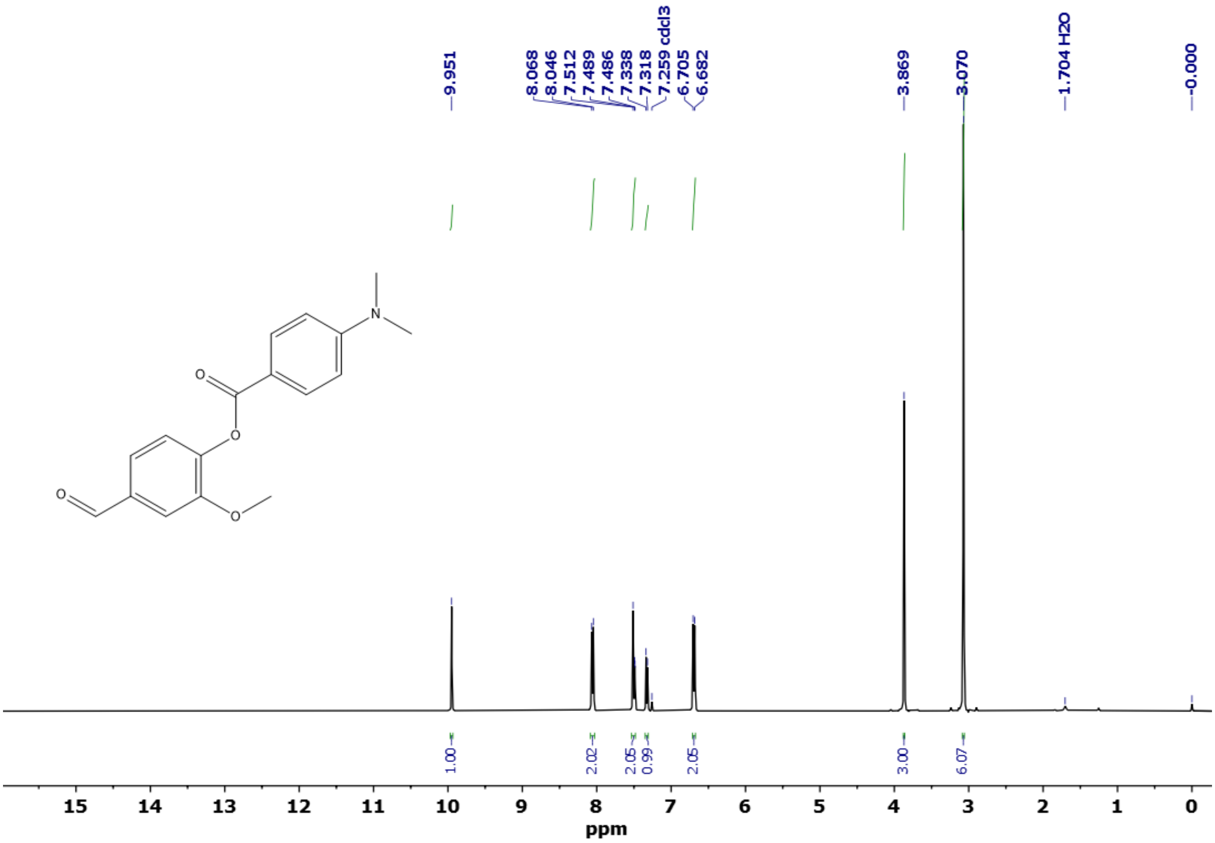

^{13}C NMR spectrum of compound **5**

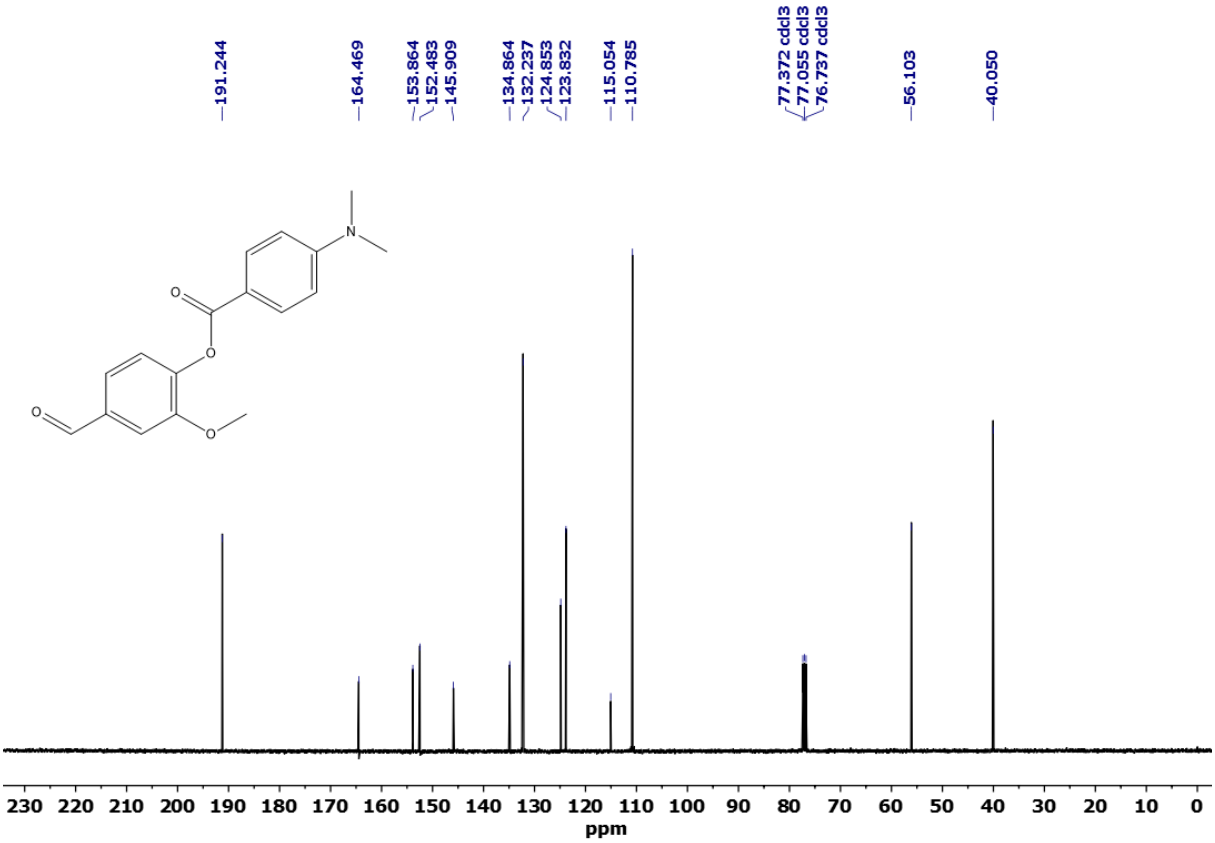

FT-IR spectrum of compound **6**

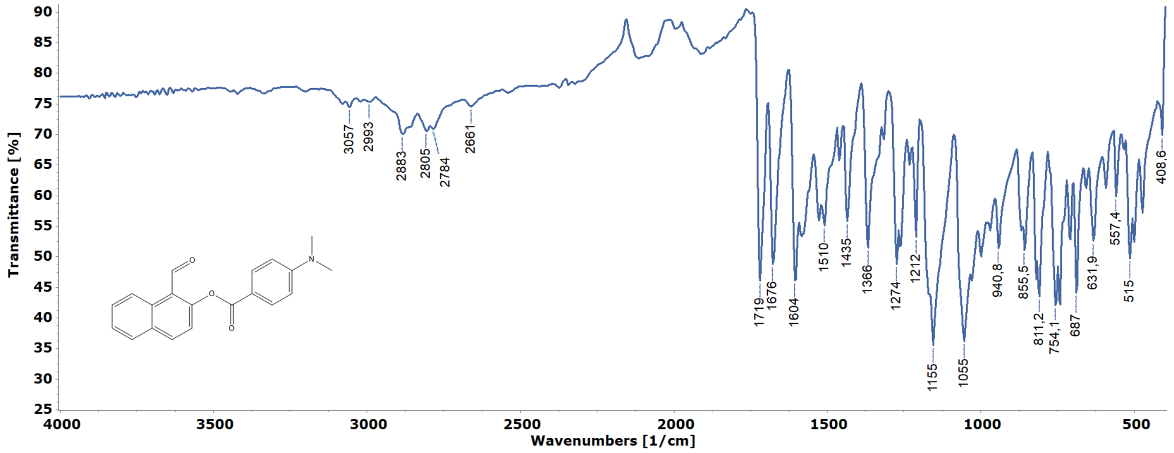

^{1}H NMR spectrum of compound **6**

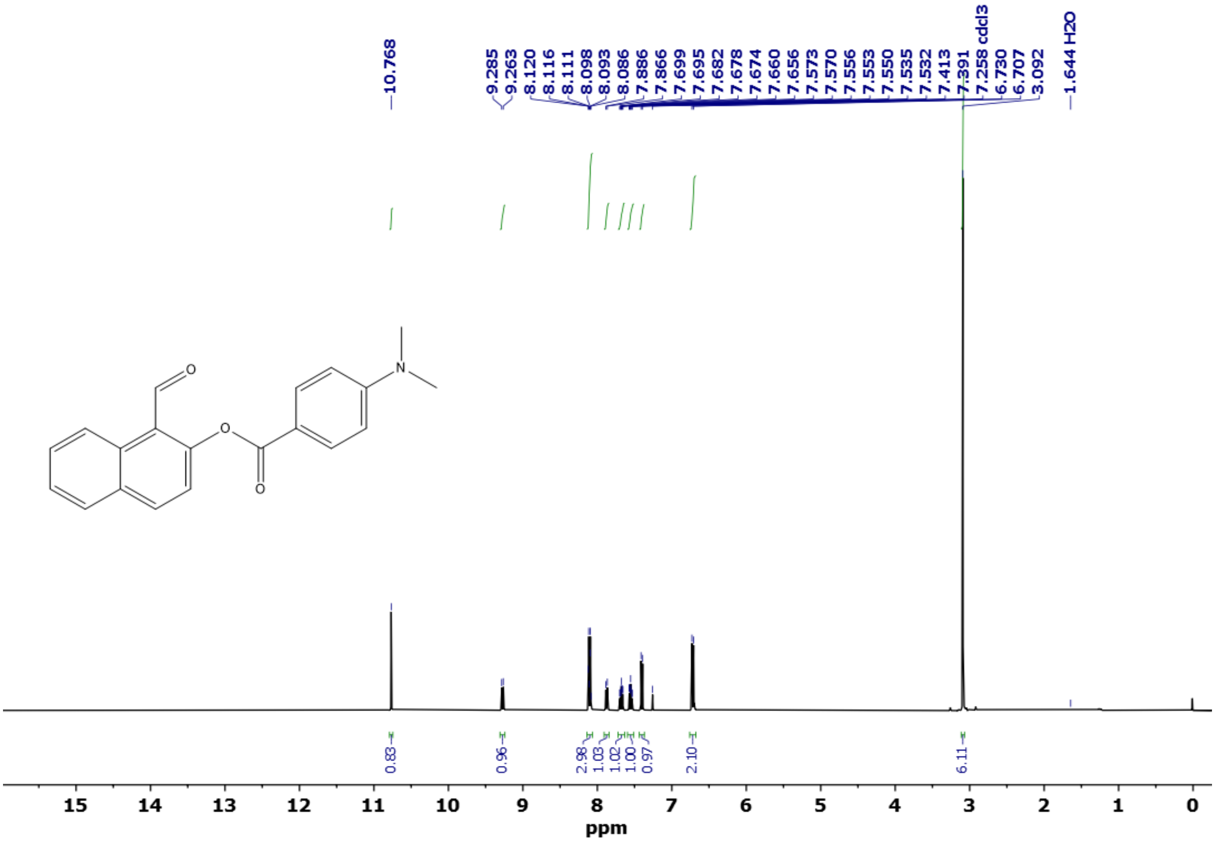

^{13}C NMR spectrum of compound **6**

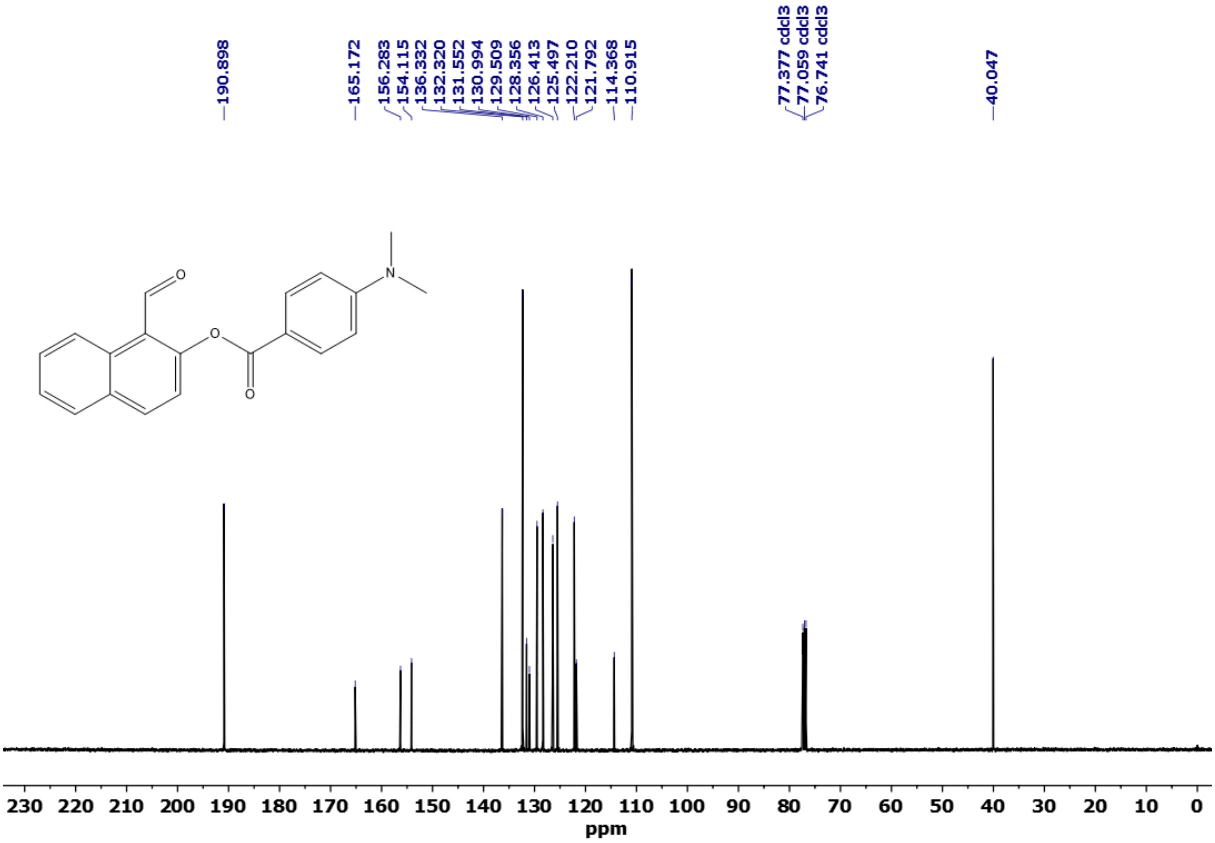

FT-IR spectrum of compound **7**

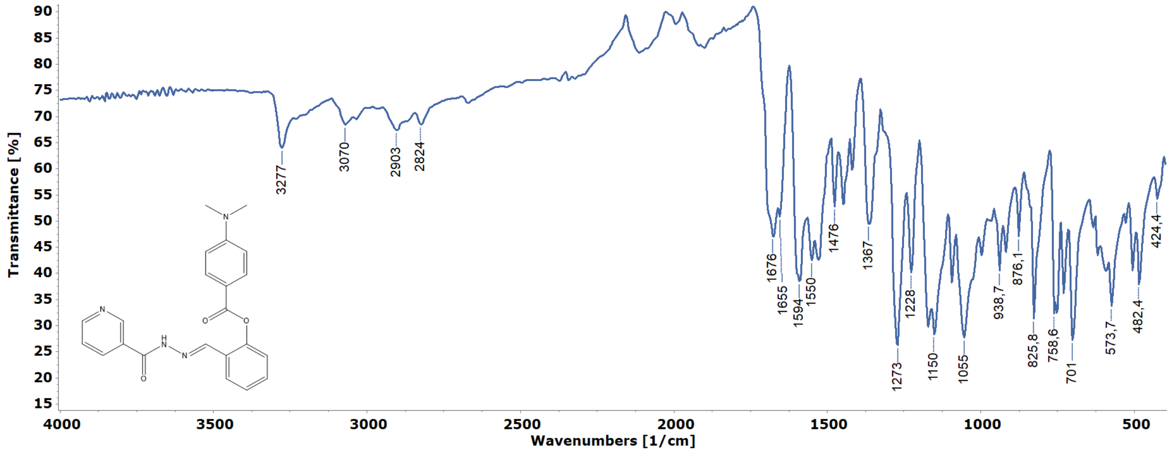

^{1}H NMR spectrum of compound **7**

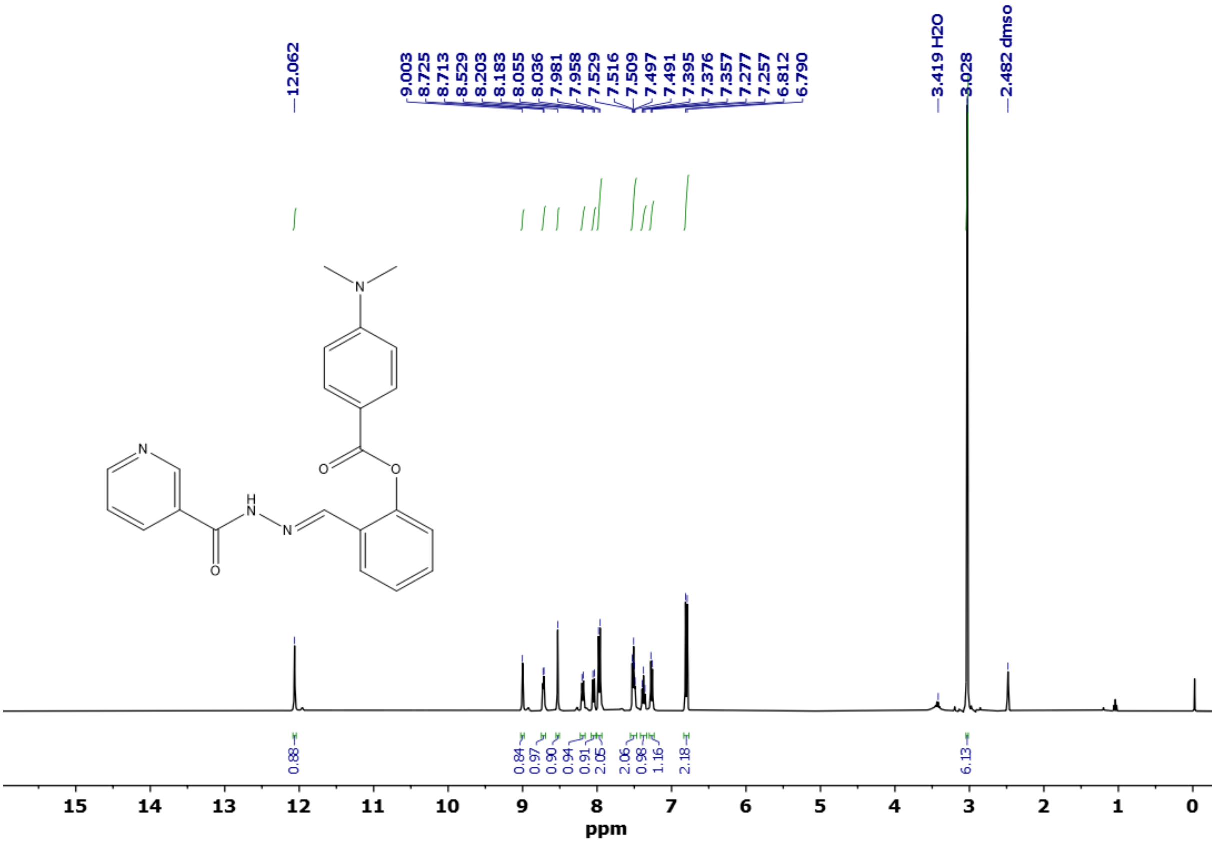

^{13}C NMR spectrum of compound **7**

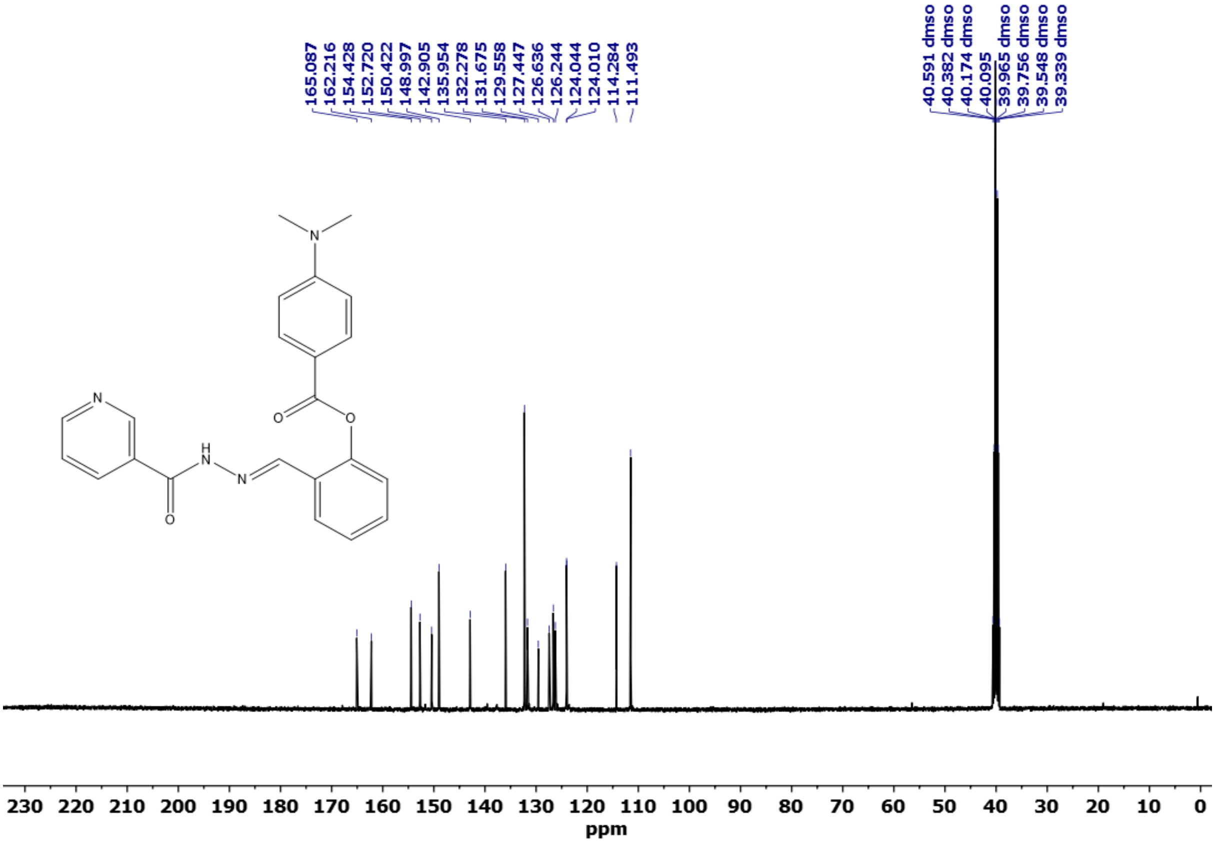

FT-IR spectrum of compound **8**

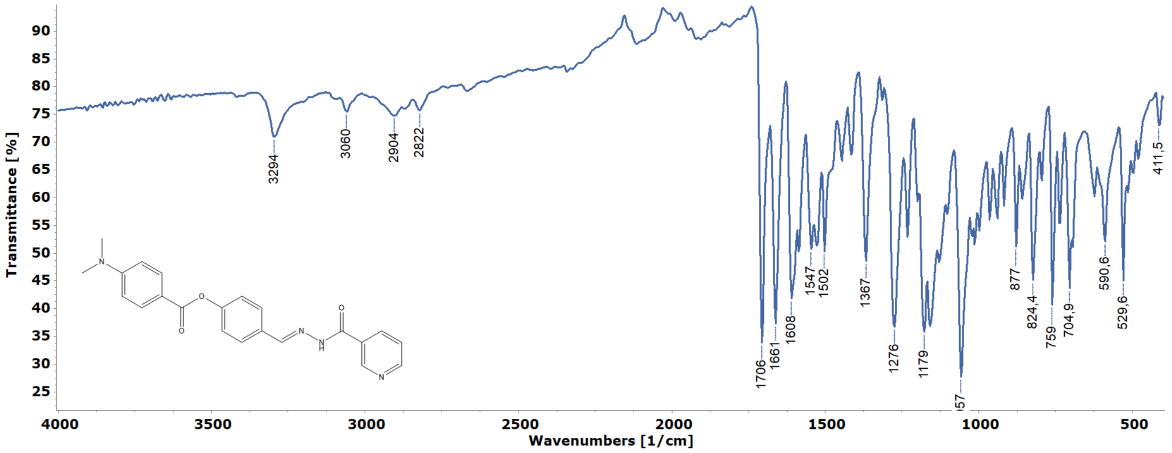

^{1}H NMR spectrum of compound **8**

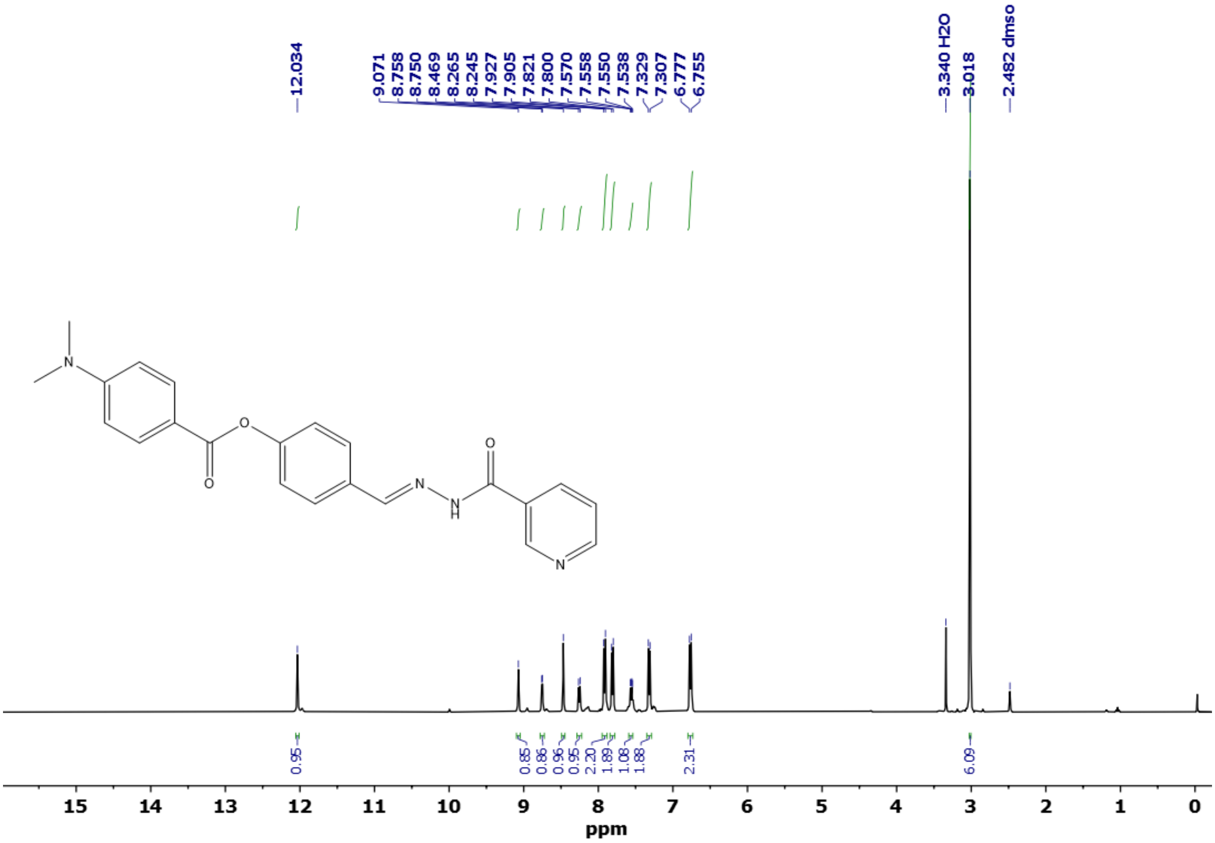

^{13}C NMR spectrum of compound **8**

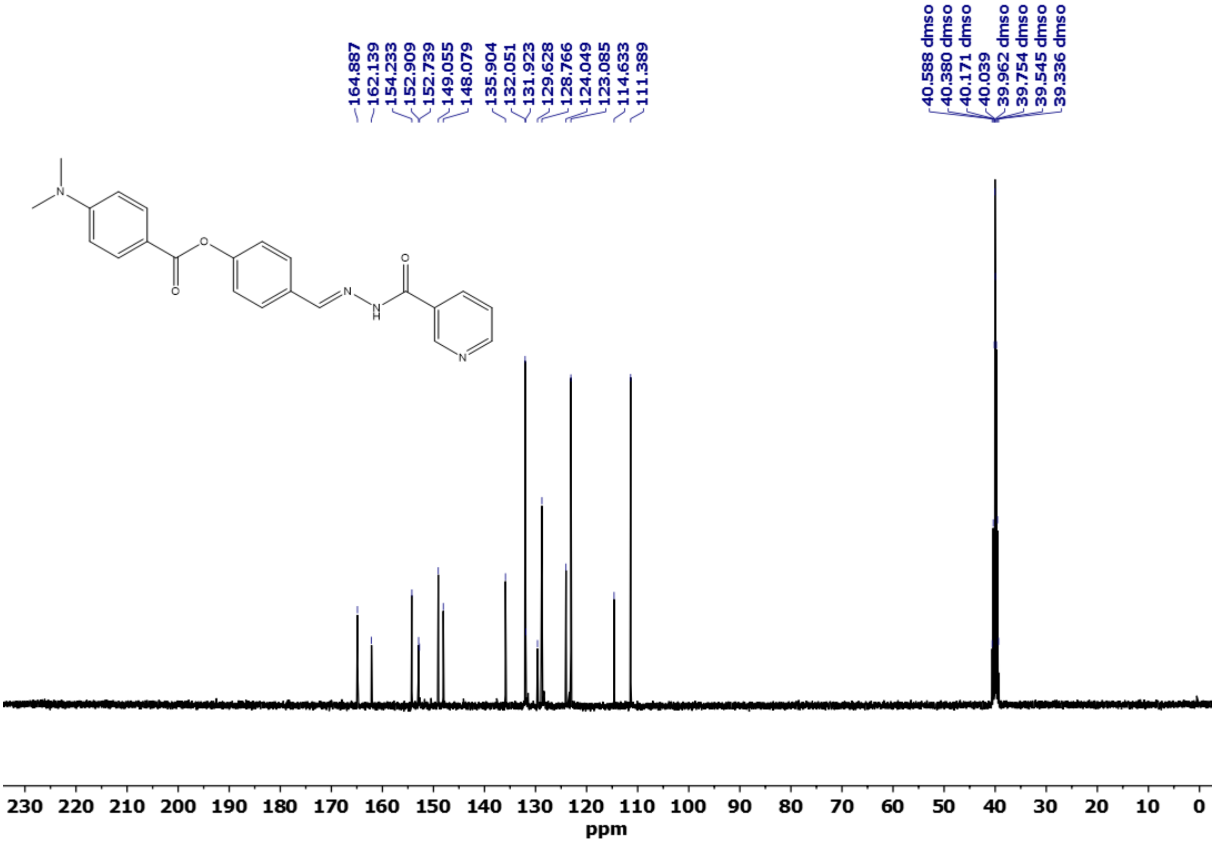

FT-IR spectrum of compound **9**

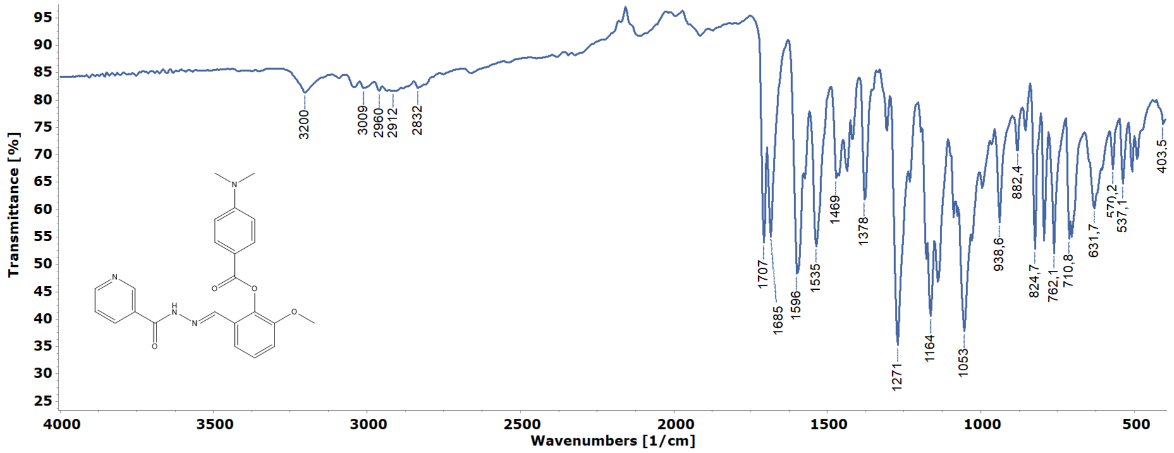

^{1}H NMR spectrum of compound **9**

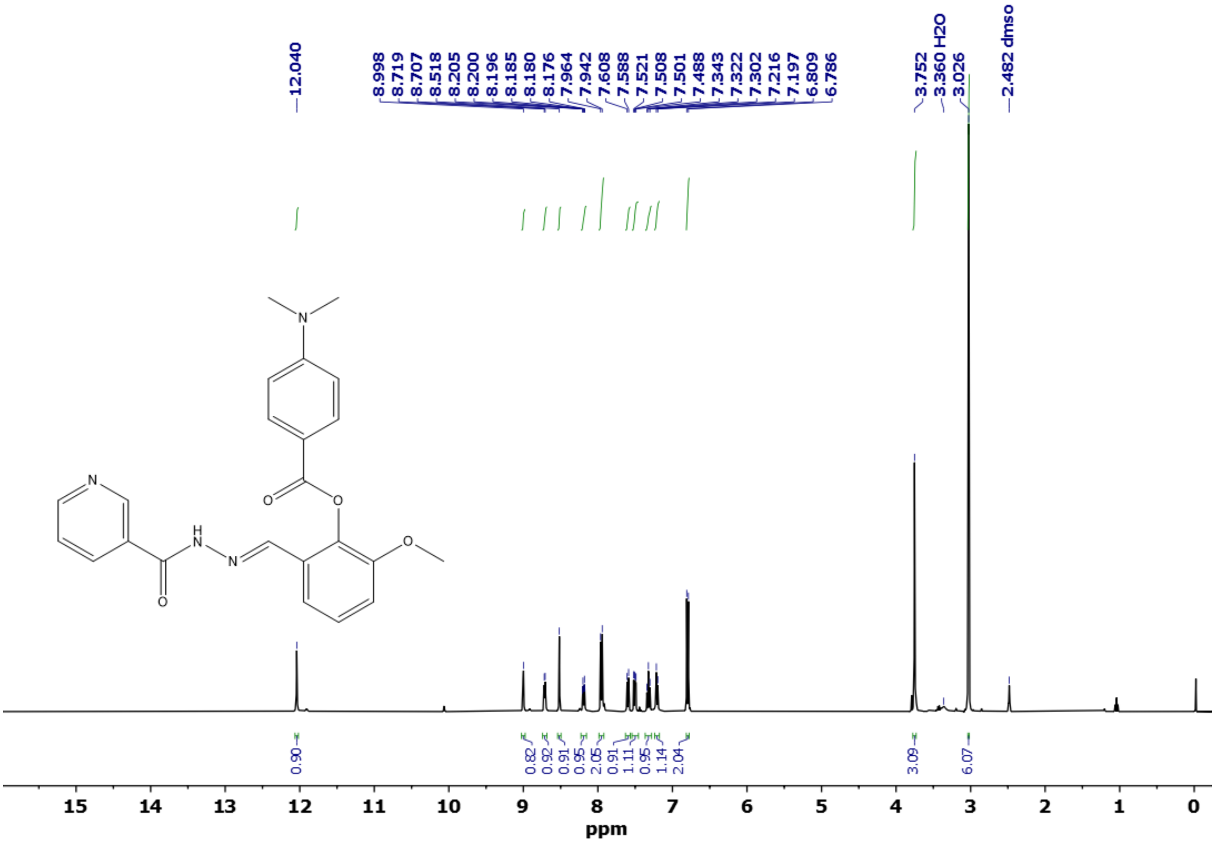

^{13}C NMR spectrum of compound **9**

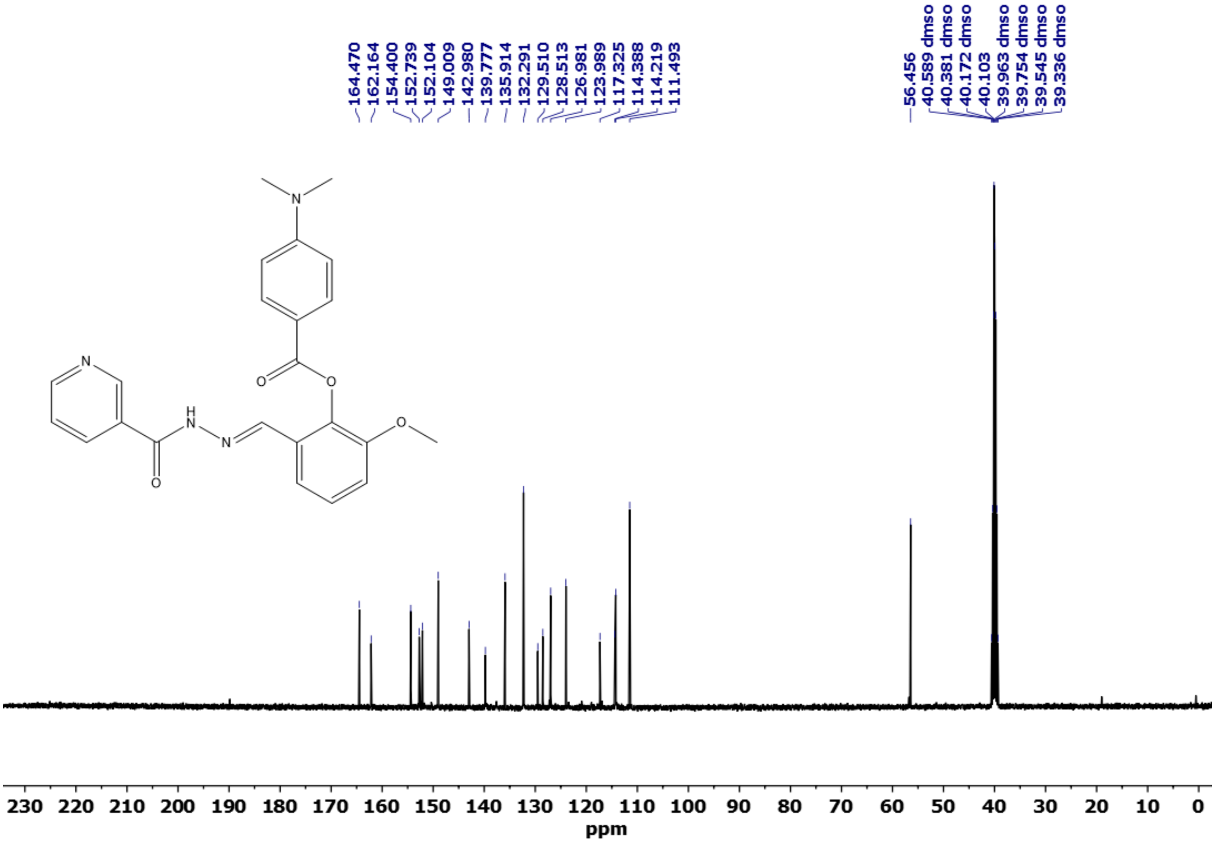

FT-IR spectrum of compound **10**

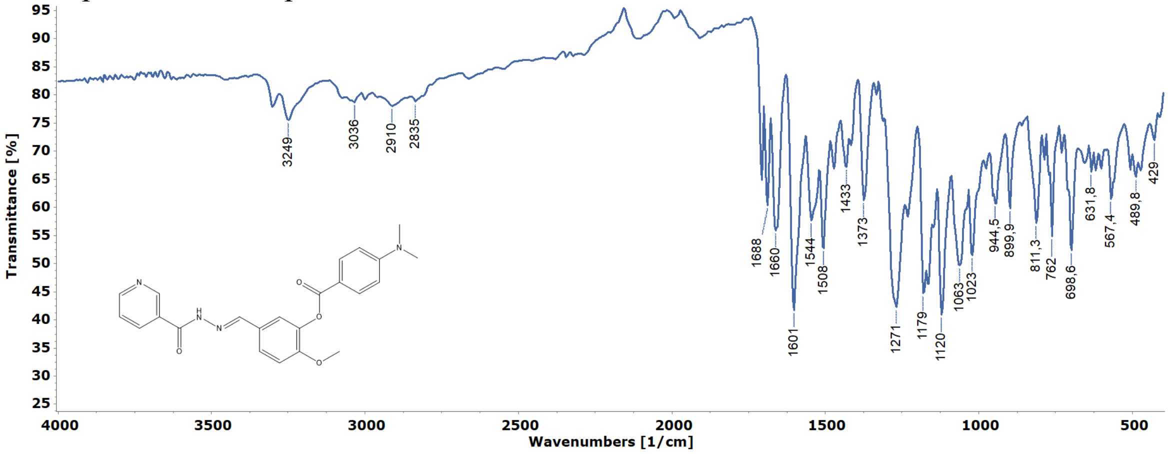

^{1}H NMR spectrum of compound **10**

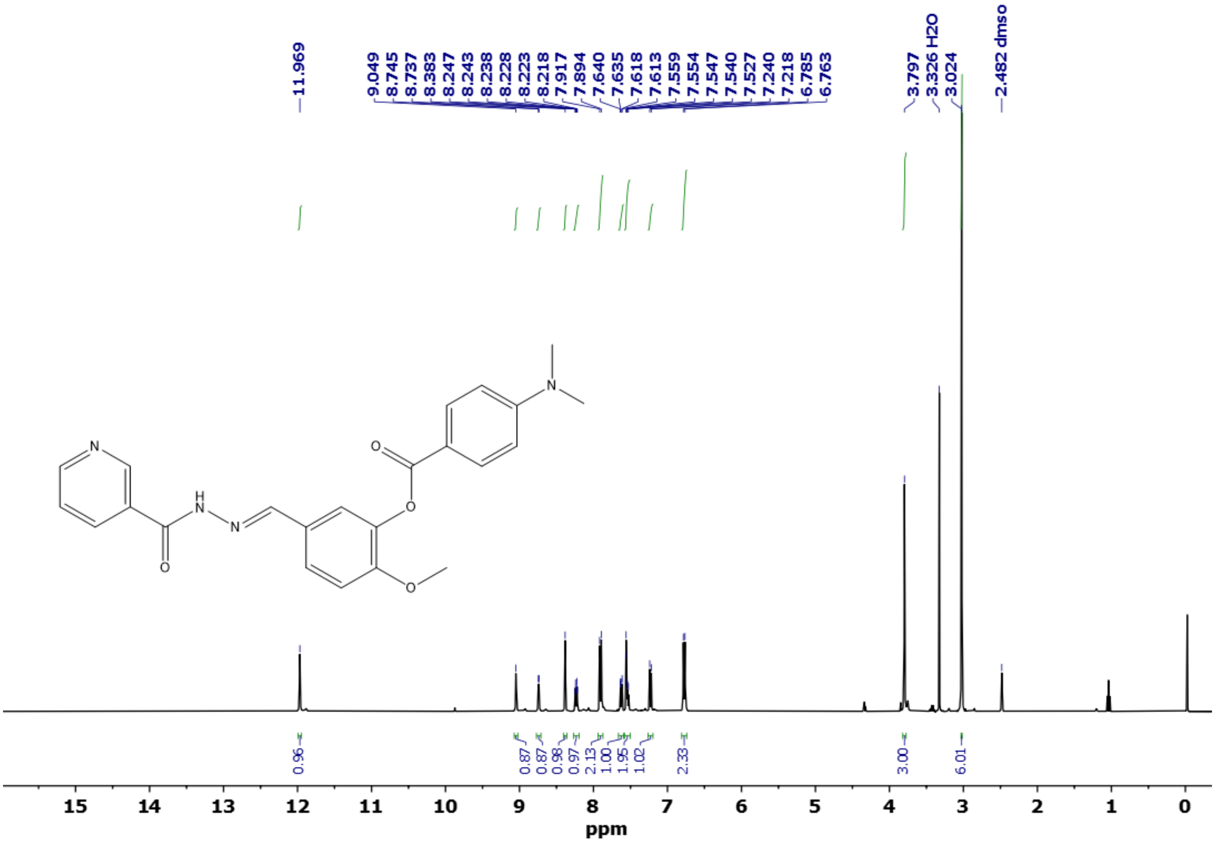

^{13}C NMR spectrum of compound **10**

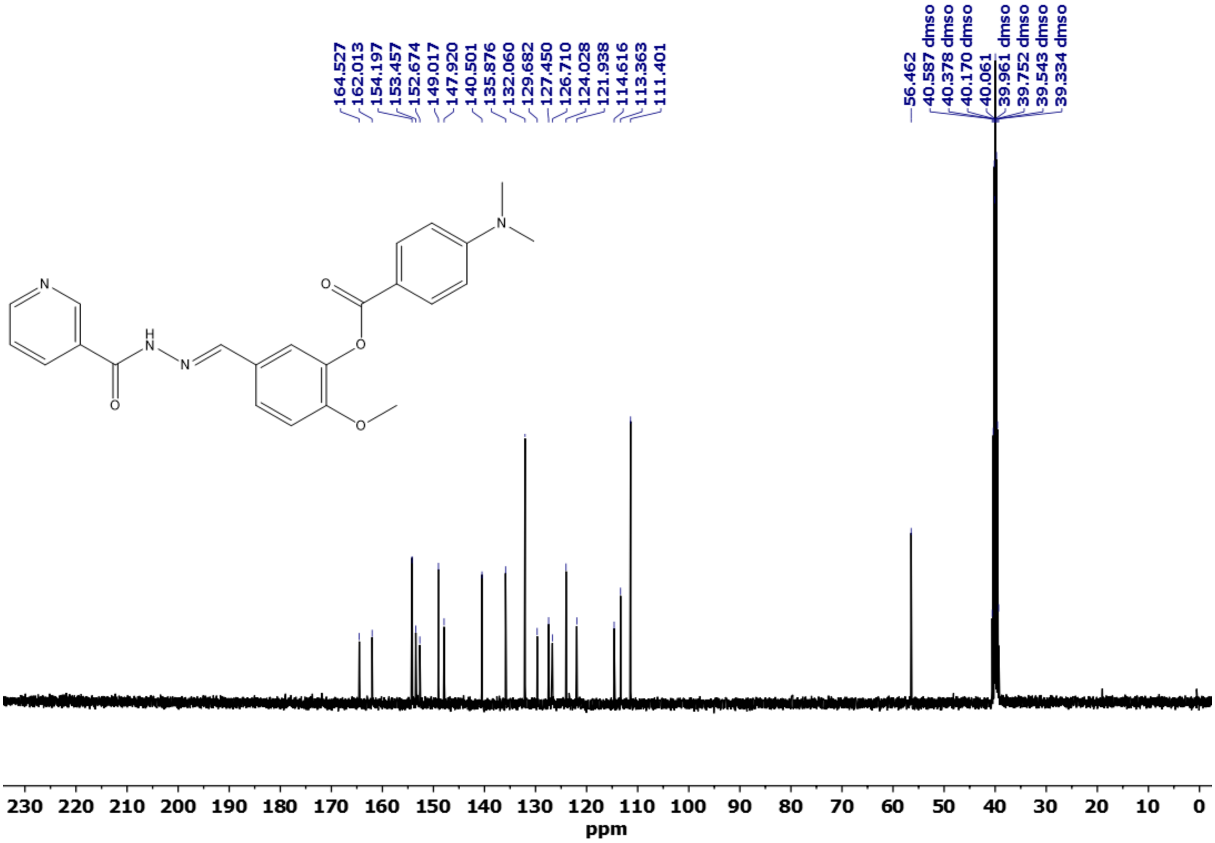

FT-IR spectrum of compound **11**

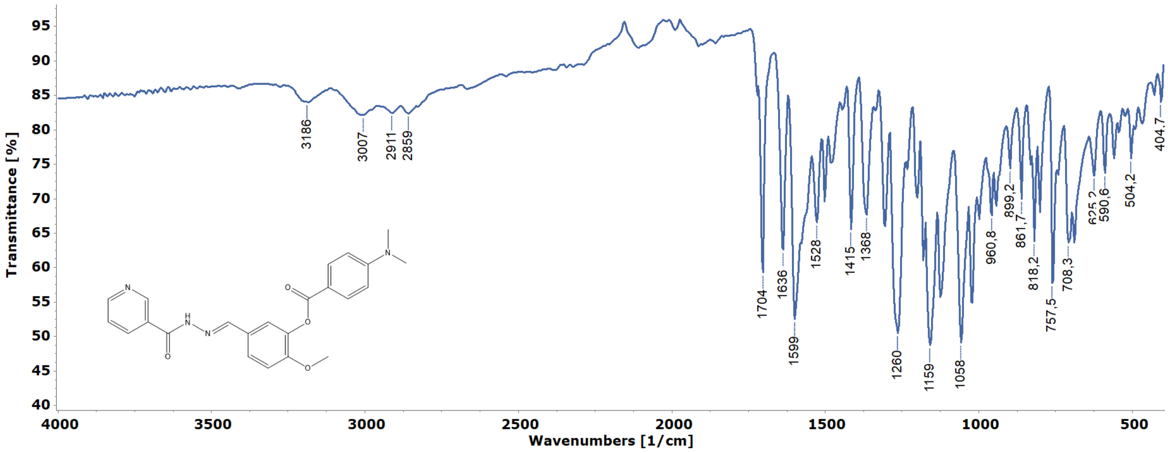

^{1}H NMR spectrum of compound **11**

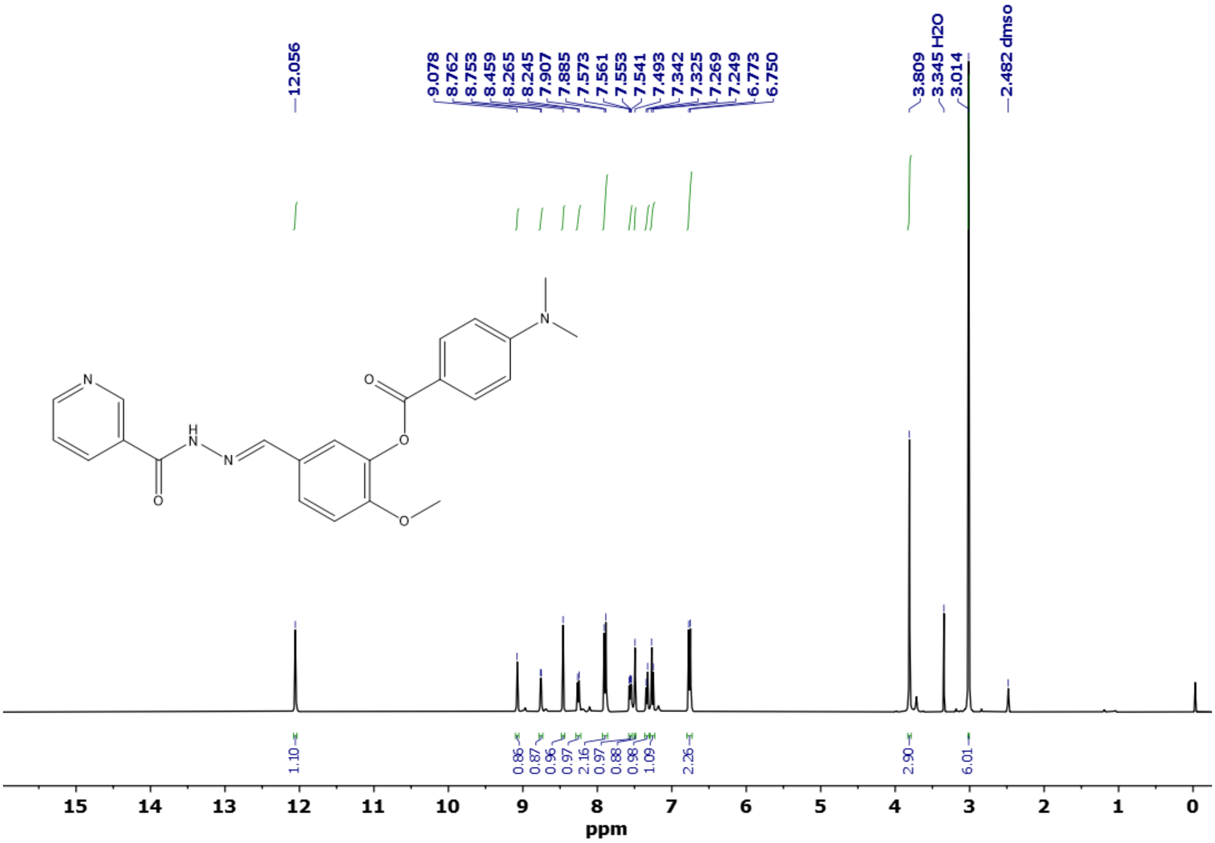

^{13}C NMR spectrum of compound **11**

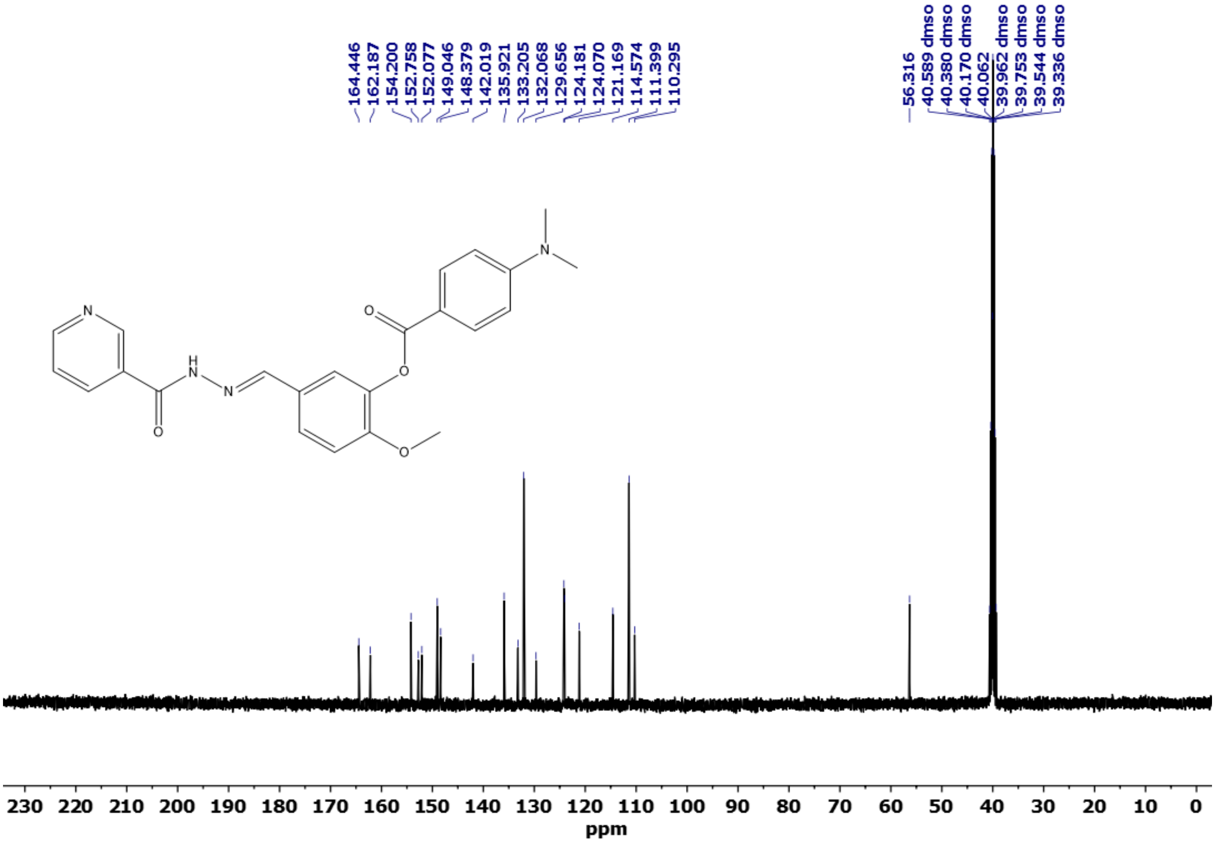

FT-IR spectrum of compound **12**

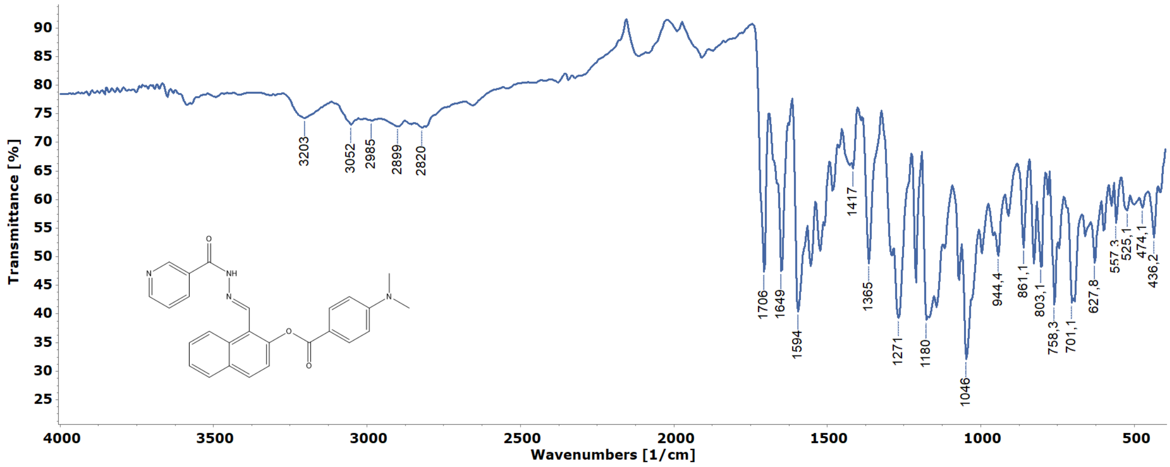

^{1}H NMR spectrum of compound **12**

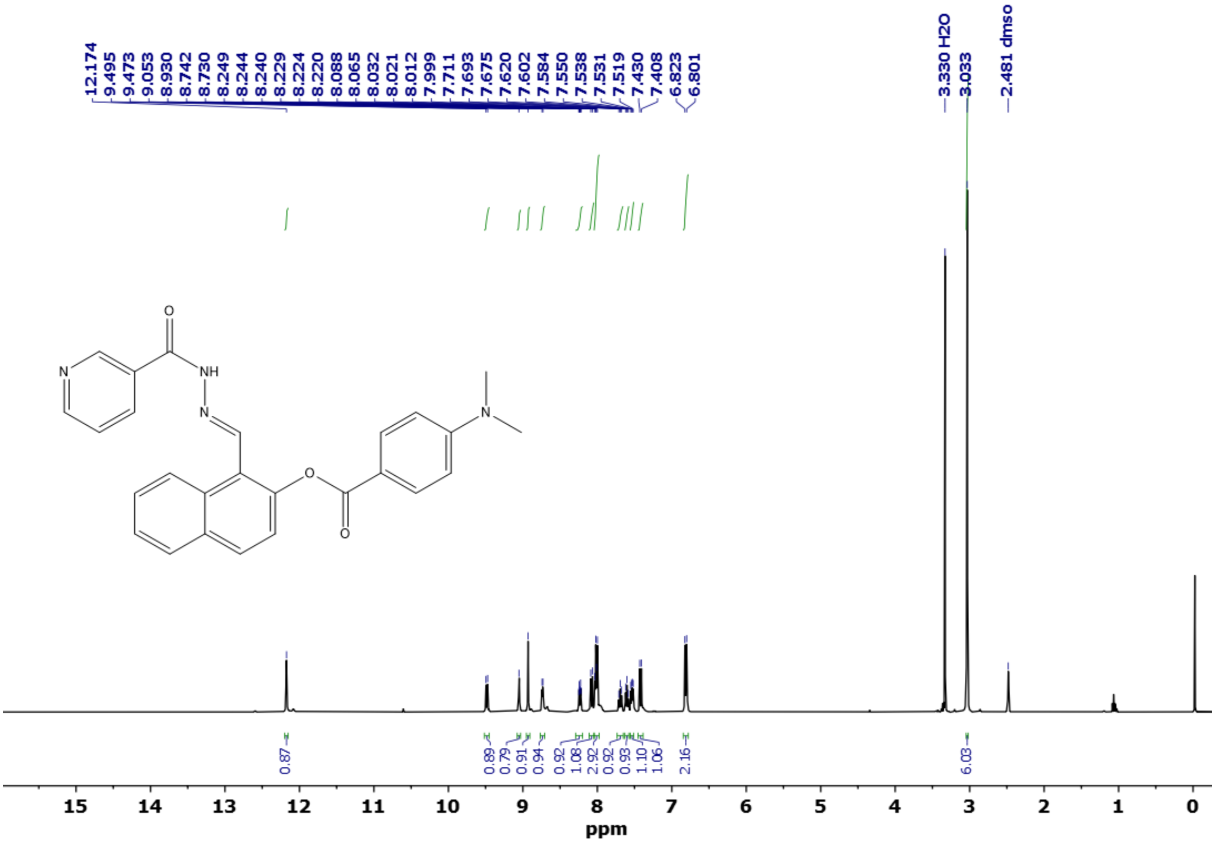

^{13}C NMR spectrum of compound **12**

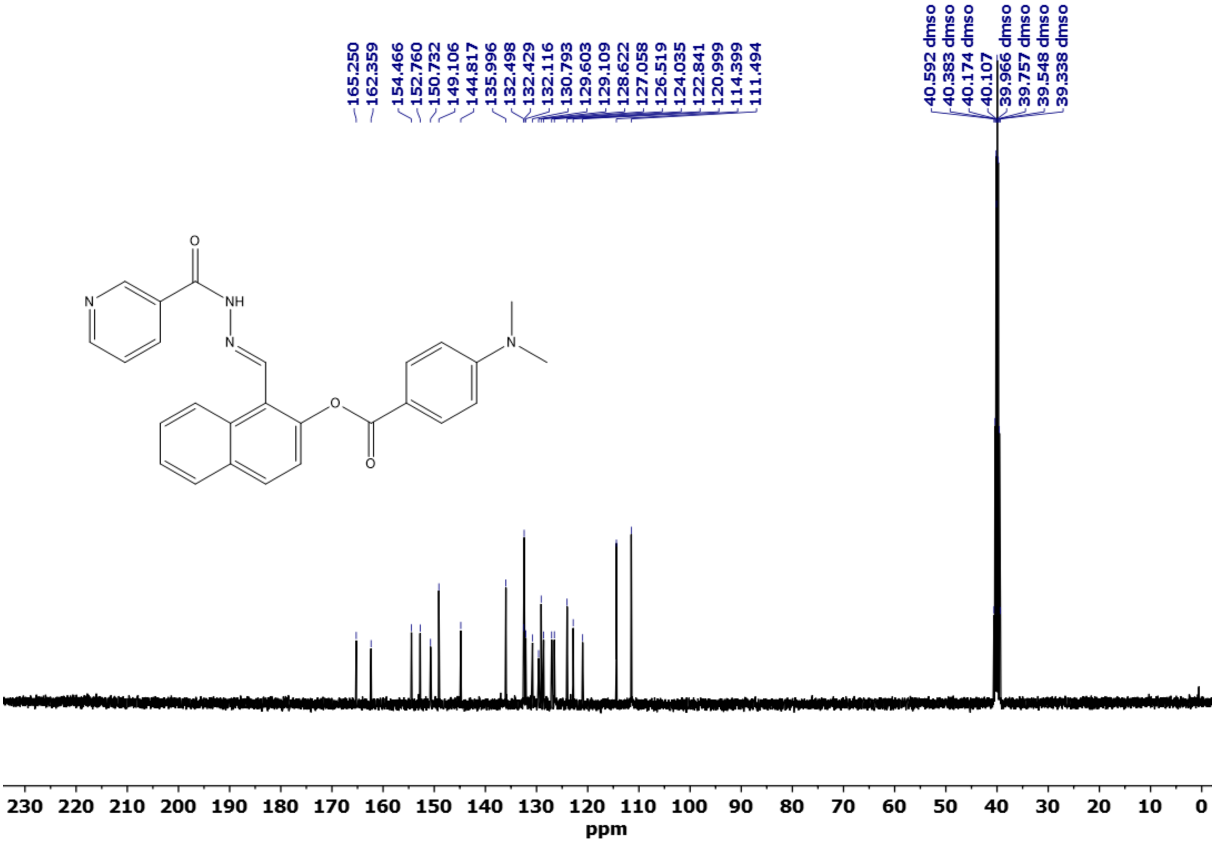

IC$_{50}$ graph of compound **1** with CA I enzyme

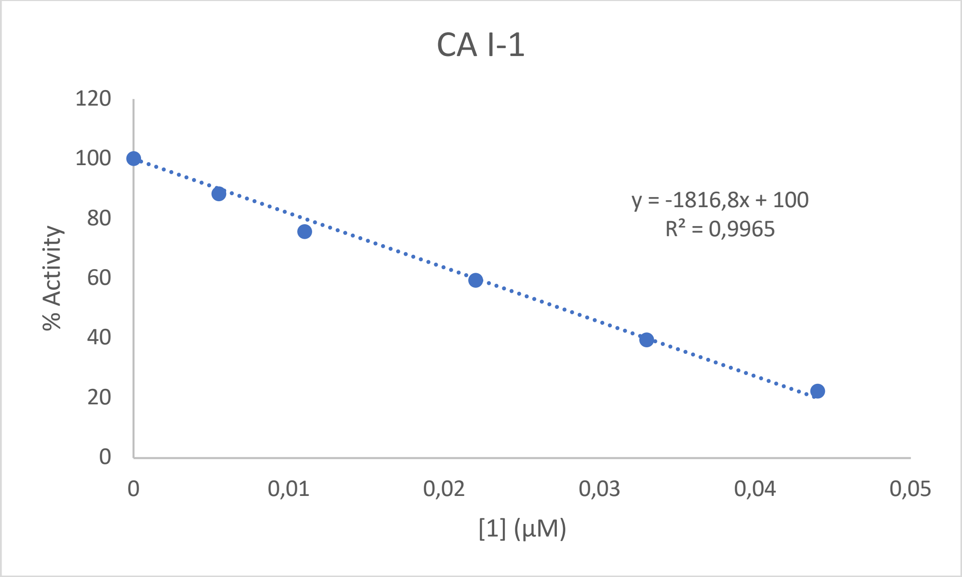

IC$_{50}$ graph of compound **2** with CA I enzyme

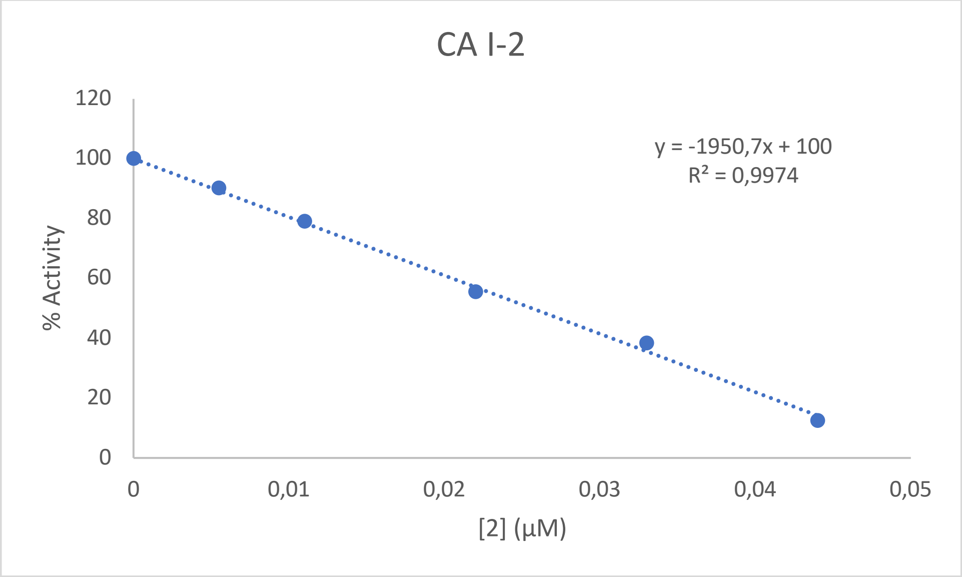

IC$_{50}$ graph of compound **3** with CA I enzyme

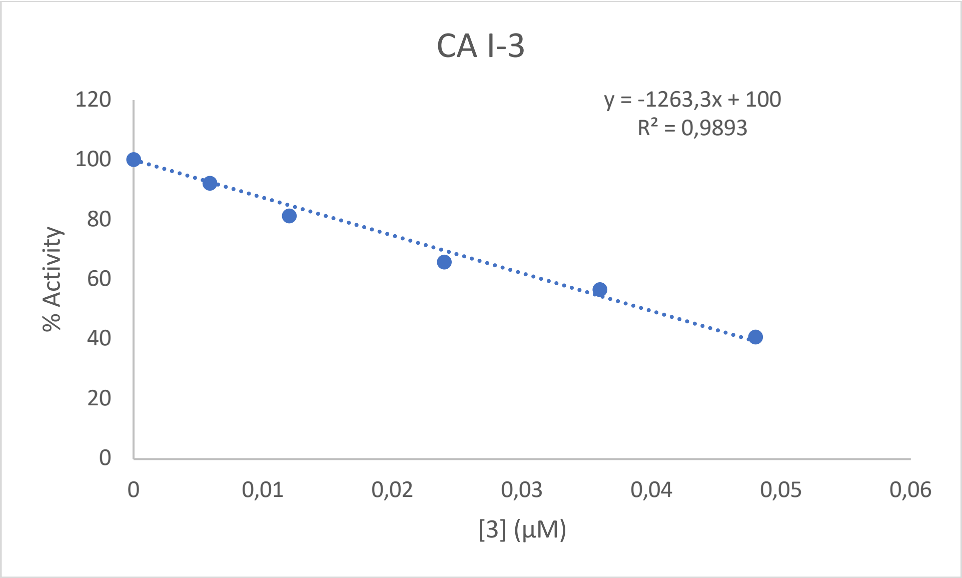

IC$_{50}$ graph of compound **4** with CA I enzyme

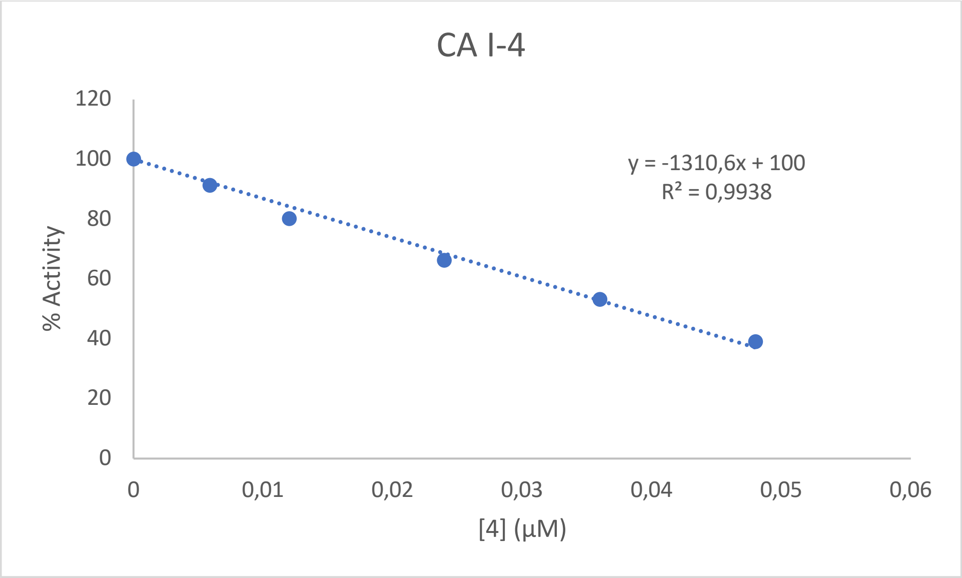

IC$_{50}$ graph of compound **5** with CA I enzyme

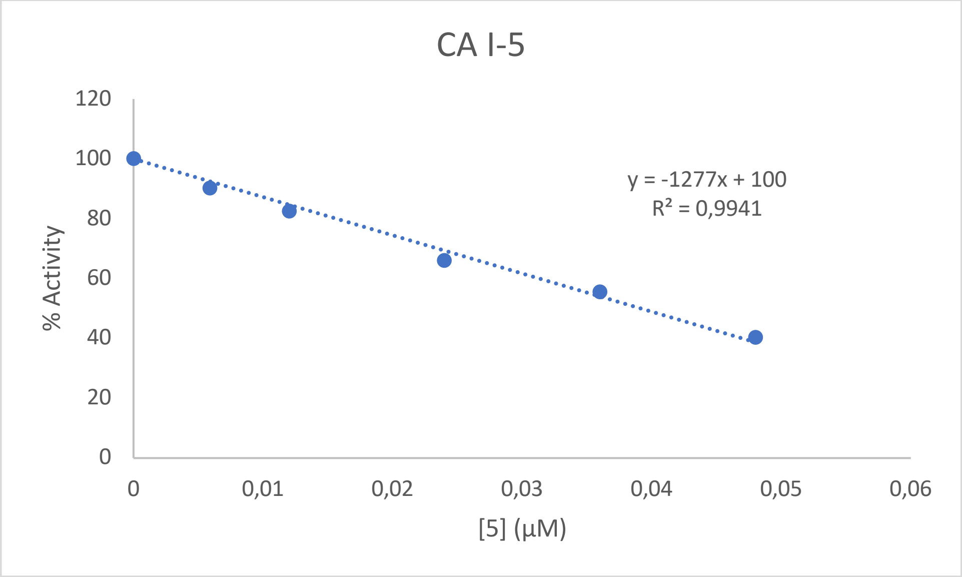

IC$_{50}$ graph of compound **6** with CA I enzyme

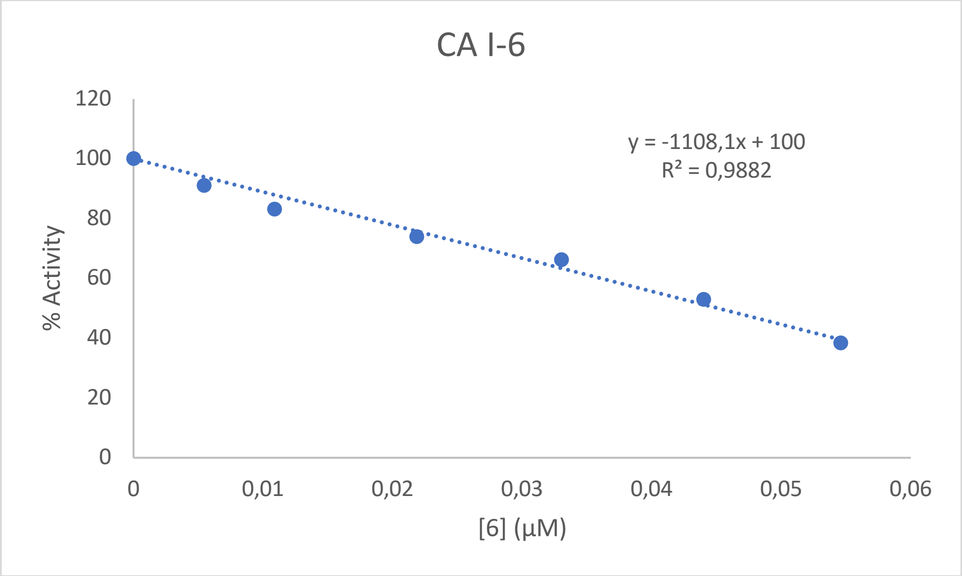

IC$_{50}$ graph of compound **7** with CA I enzyme

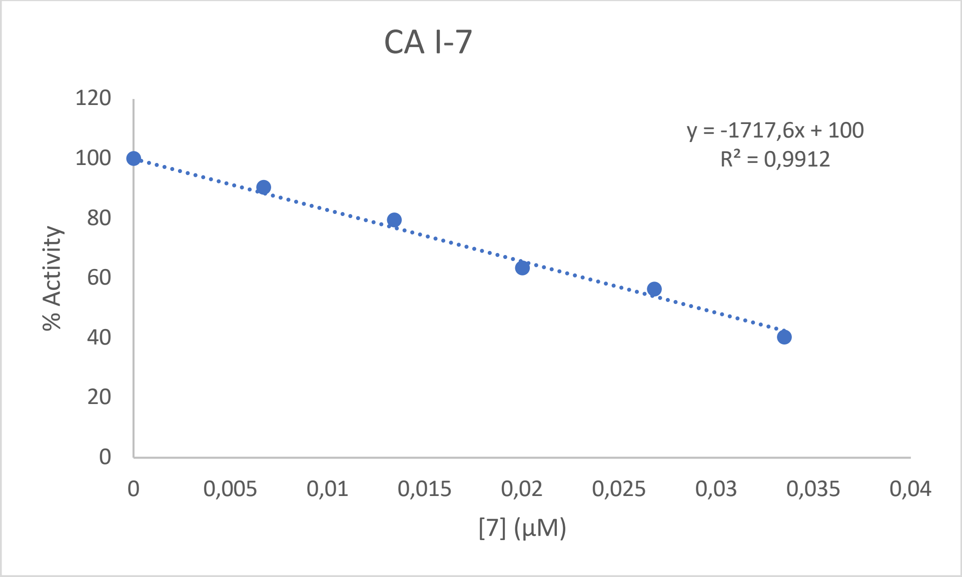

IC$_{50}$ graph of compound **8** with CA I enzyme

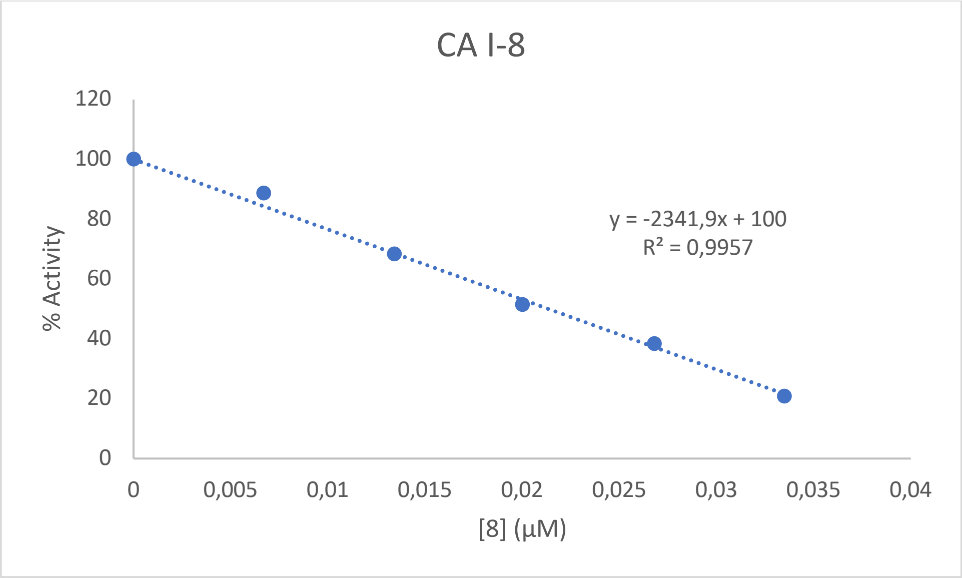

IC$_{50}$ graph of compound **9** with CA I enzyme

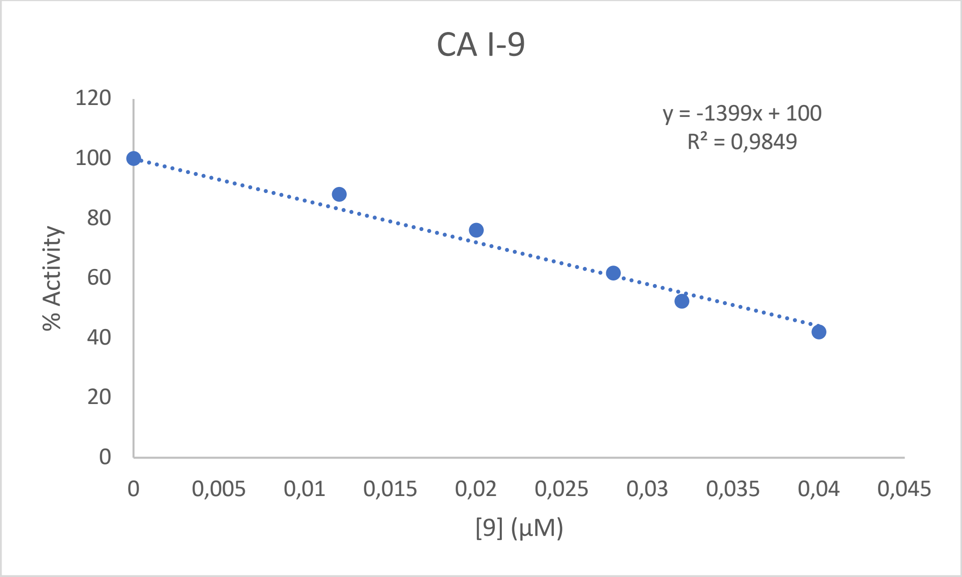

IC$_{50}$ graph of compound **10** with CA I enzyme

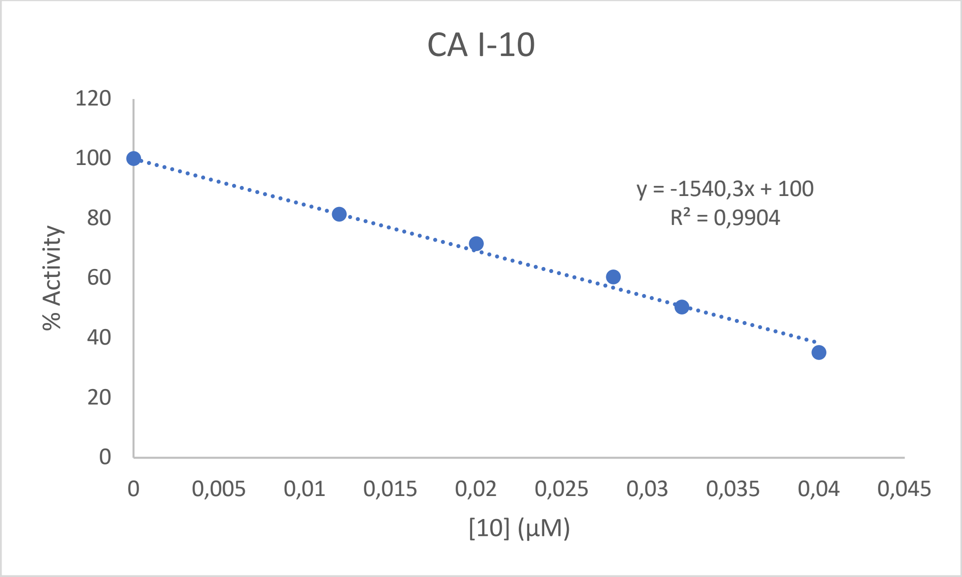

IC$_{50}$ graph of compound **11** with CA I enzyme

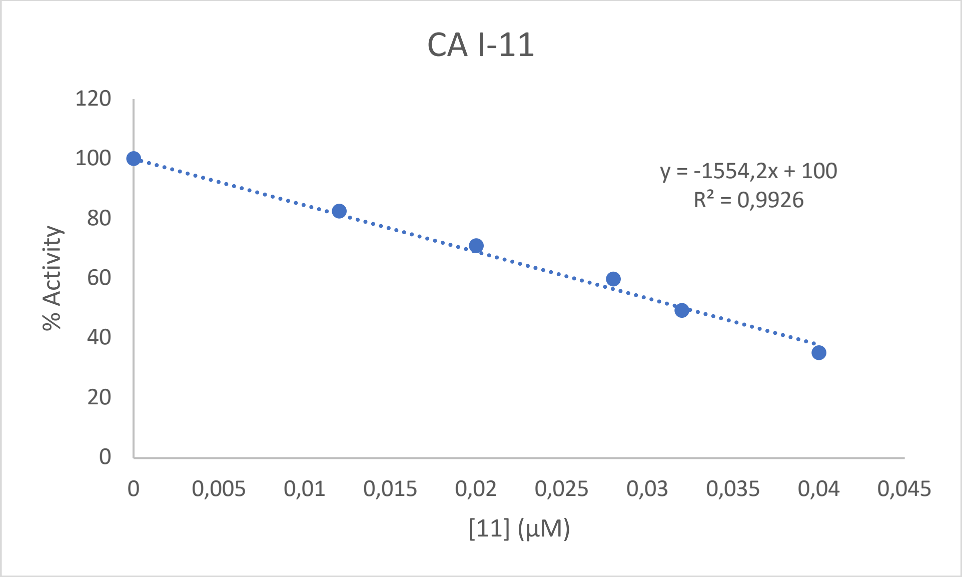

IC$_{50}$ graph of compound **12** with CA I enzyme

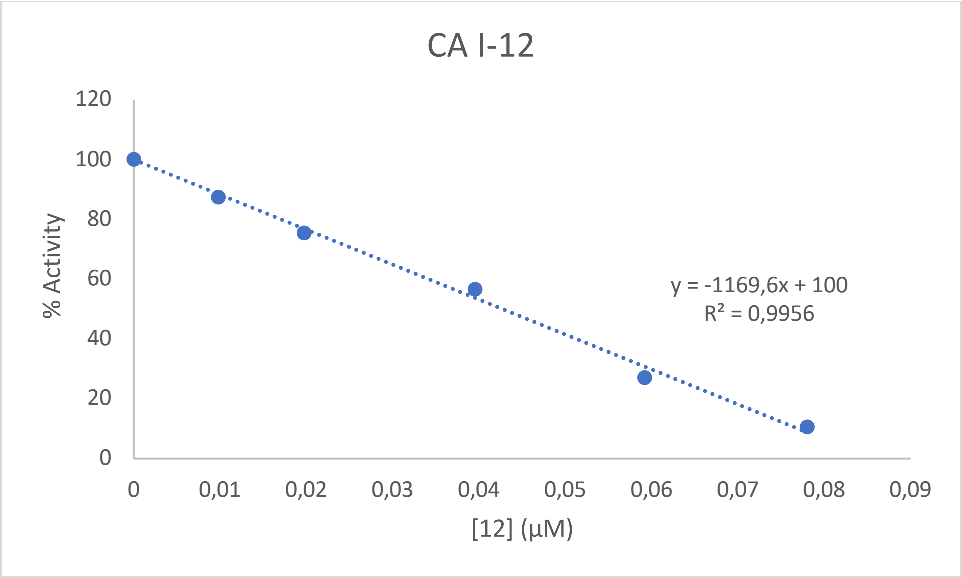

IC$_{50}$ graph of compound **AZA** with CA I enzyme

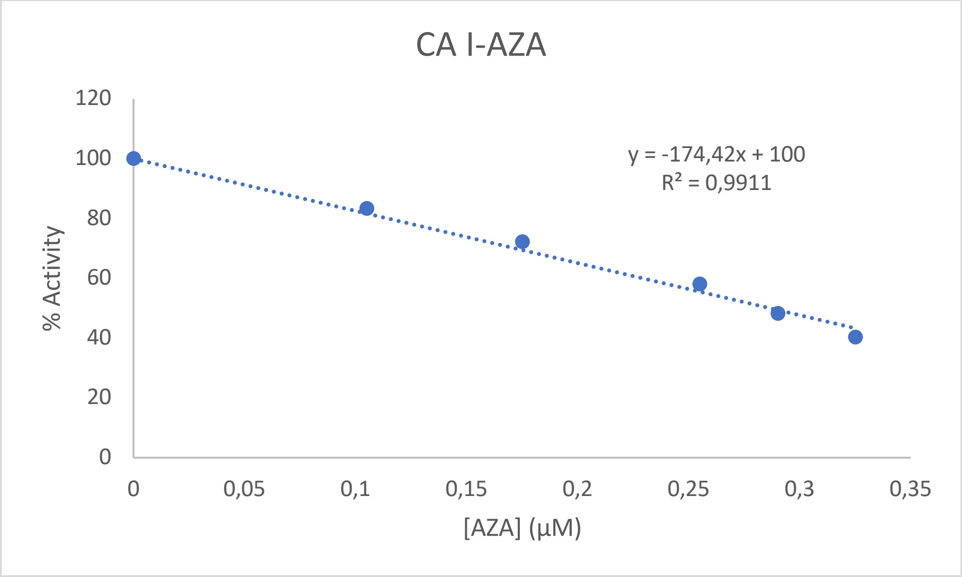

IC$_{50}$ graph of compound **1** with CA II enzyme

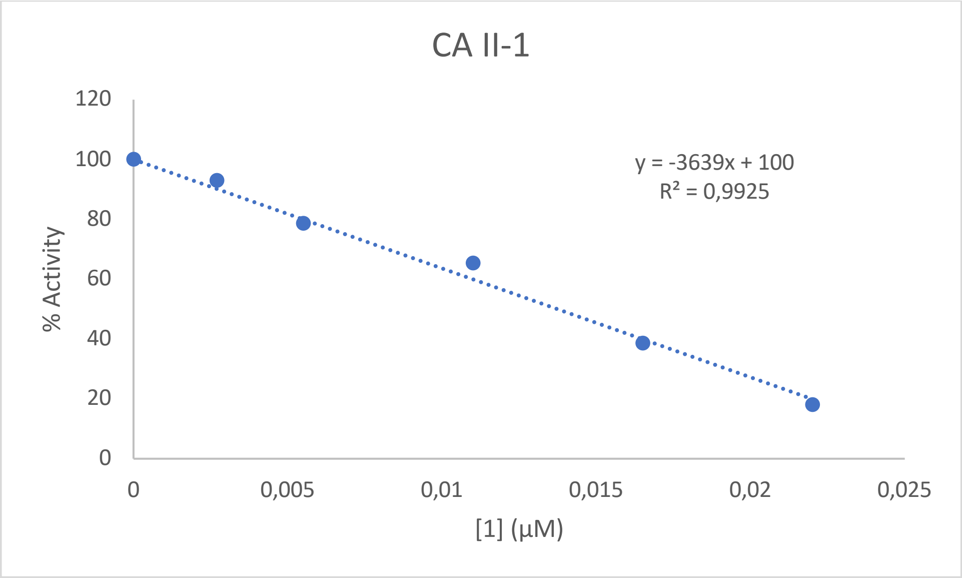

IC_{50} graph of compound **2** with CA II enzyme

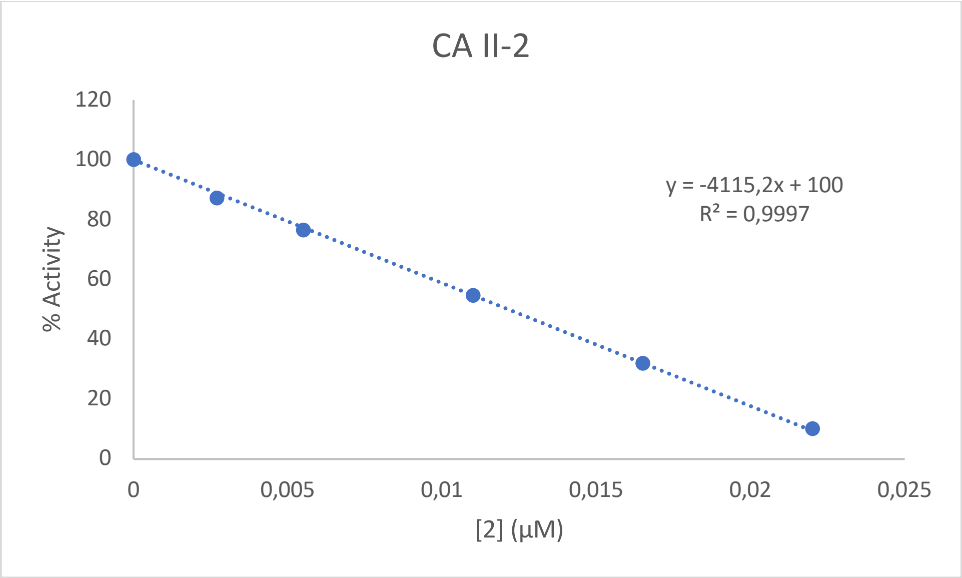

IC_{50} graph of compound **3** with CA II enzyme

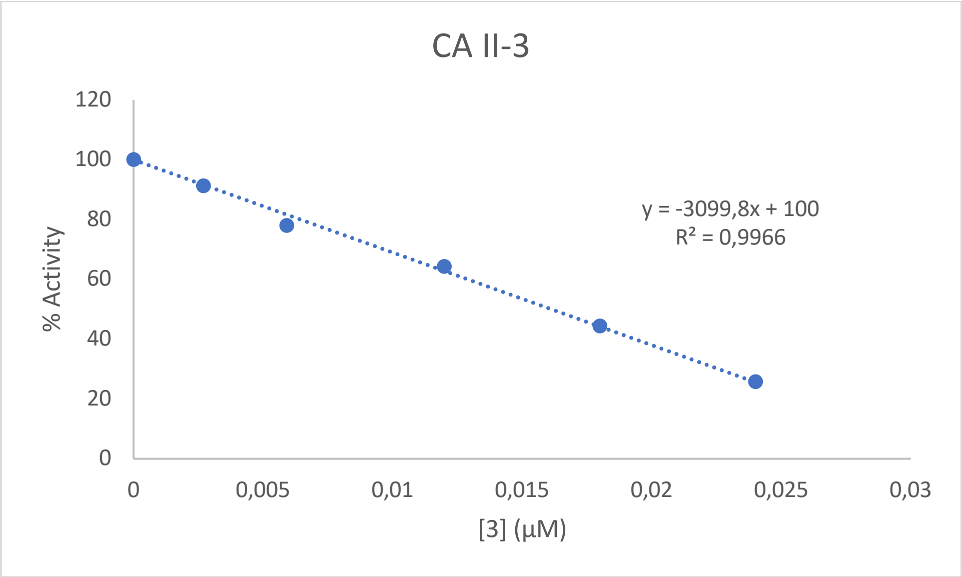

IC$_{50}$ graph of compound **4** with CA II enzyme

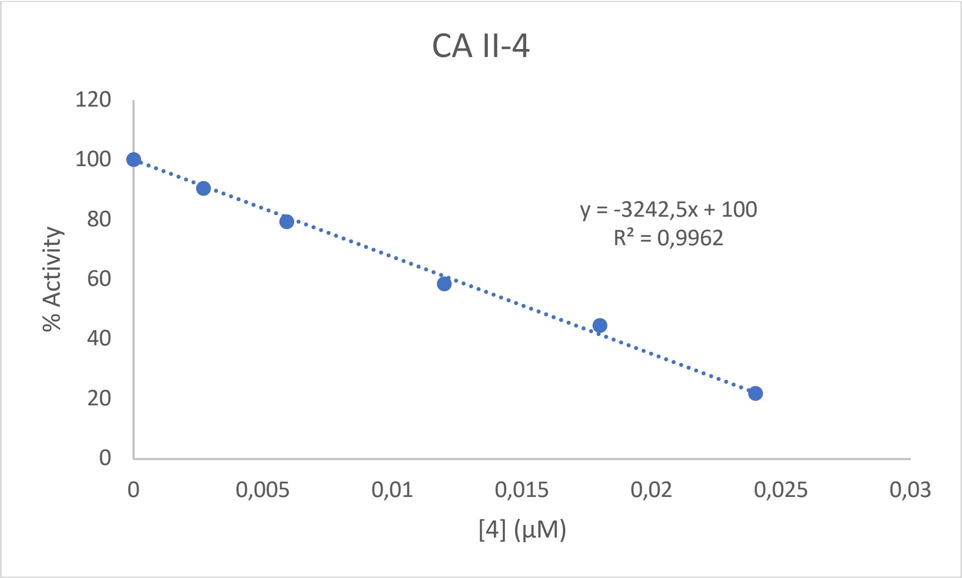

IC$_{50}$ graph of compound **5** with CA II enzyme

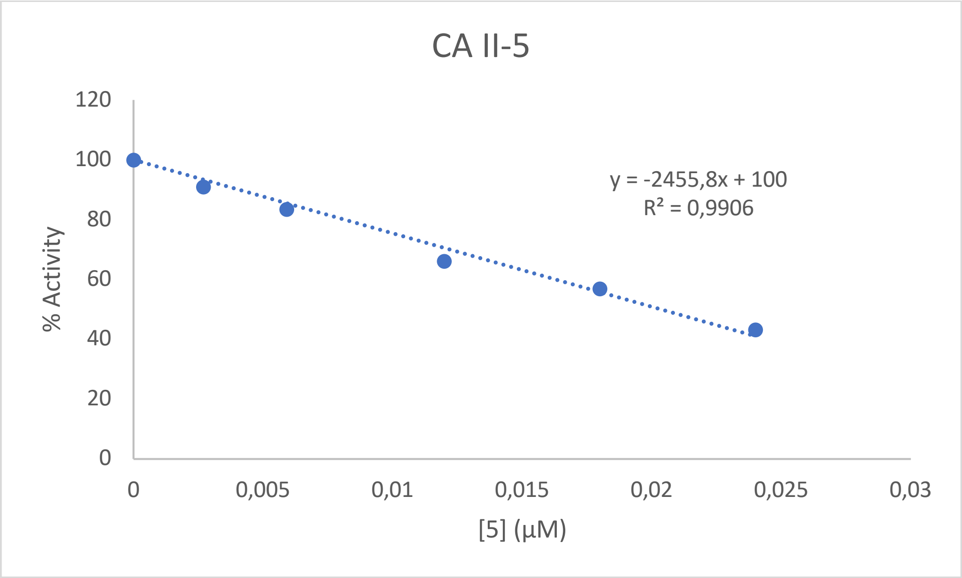

IC$_{50}$ graph of compound **6** with CA II enzyme

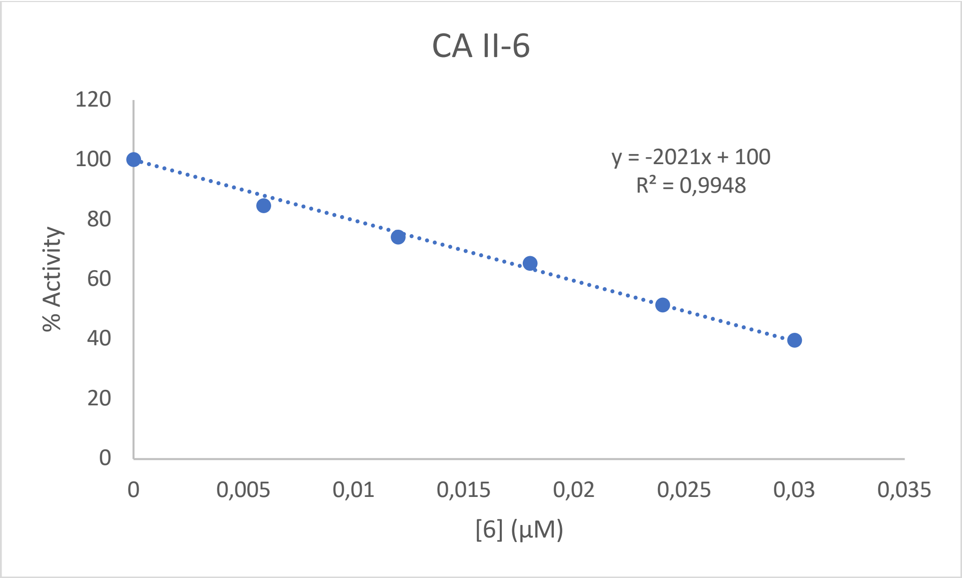

IC$_{50}$ graph of compound **7** with CA II enzyme

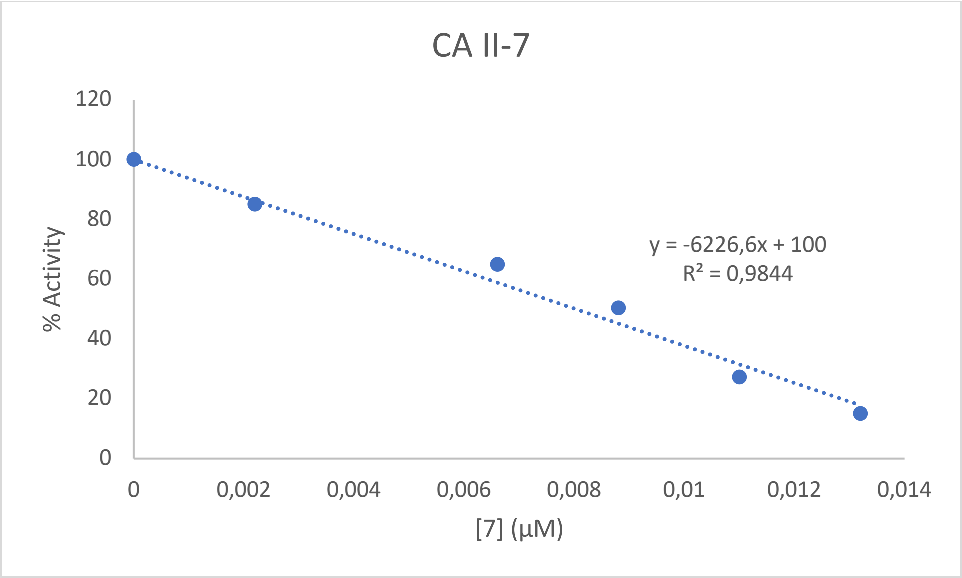

IC$_{50}$ graph of compound **8** with CA II enzyme

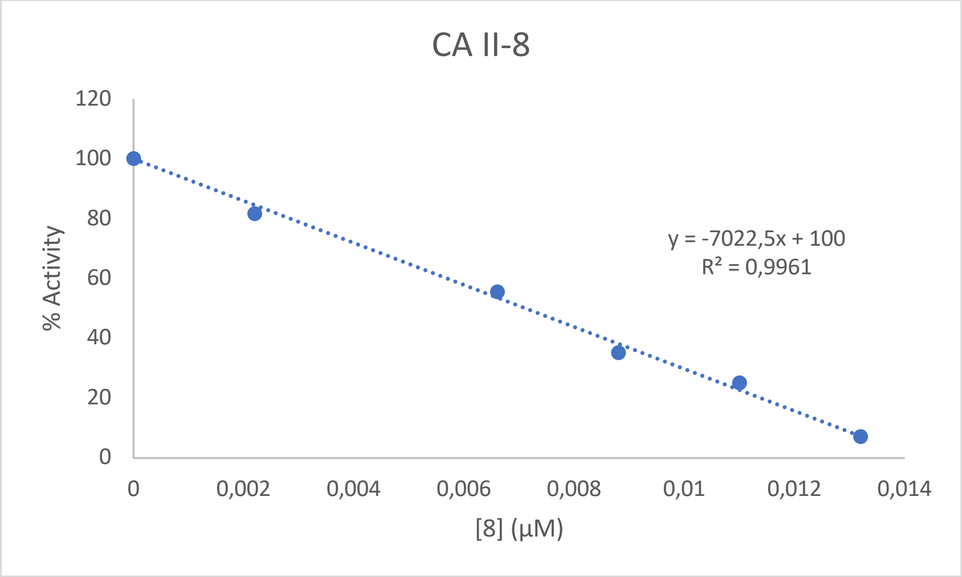

IC$_{50}$ graph of compound **9** with CA II enzyme

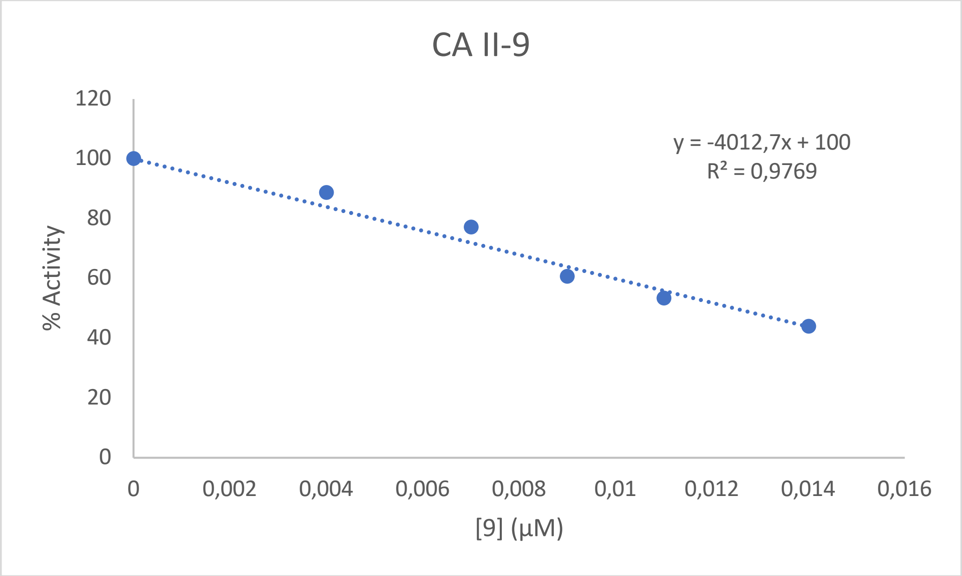

IC$_{50}$ graph of compound **10** with CA II enzyme

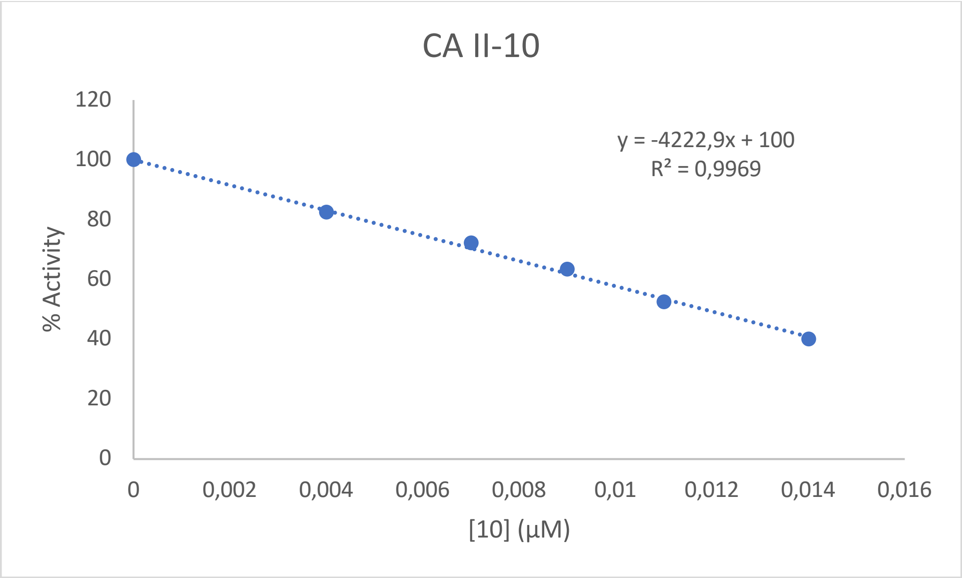

IC$_{50}$ graph of compound **11** with CA II enzyme

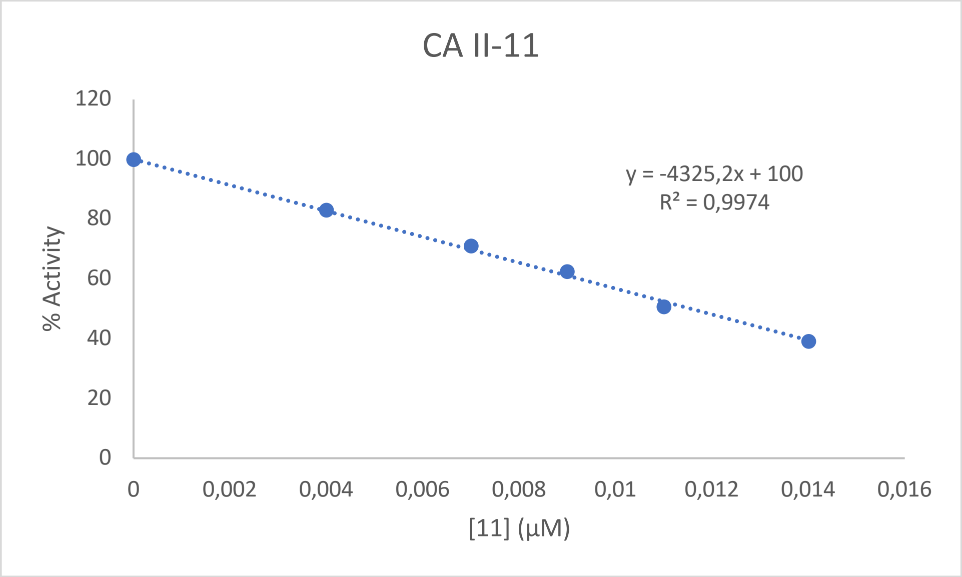

IC$_{50}$ graph of compound **12** with CA II enzyme

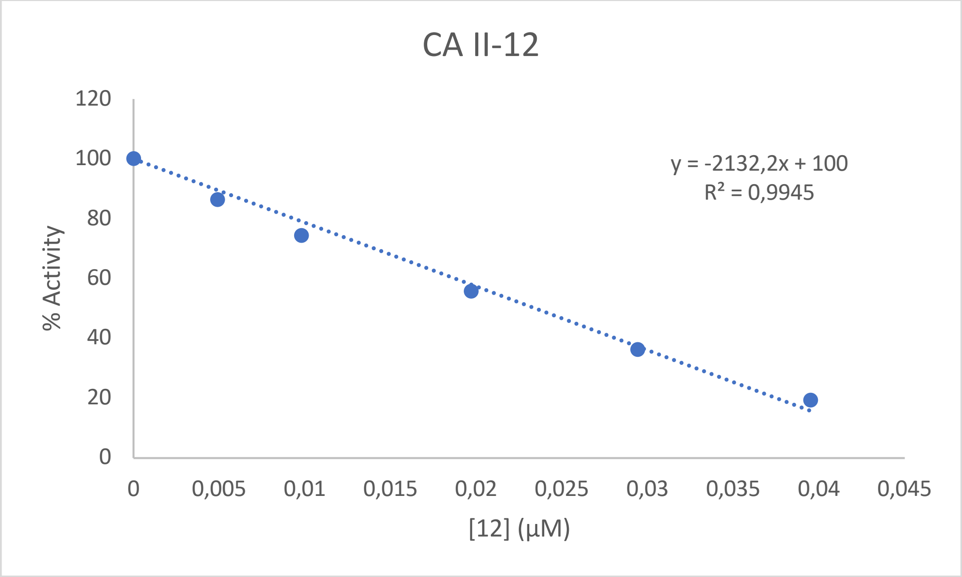

IC$_{50}$ graph of compound **AZA** with CA II enzyme

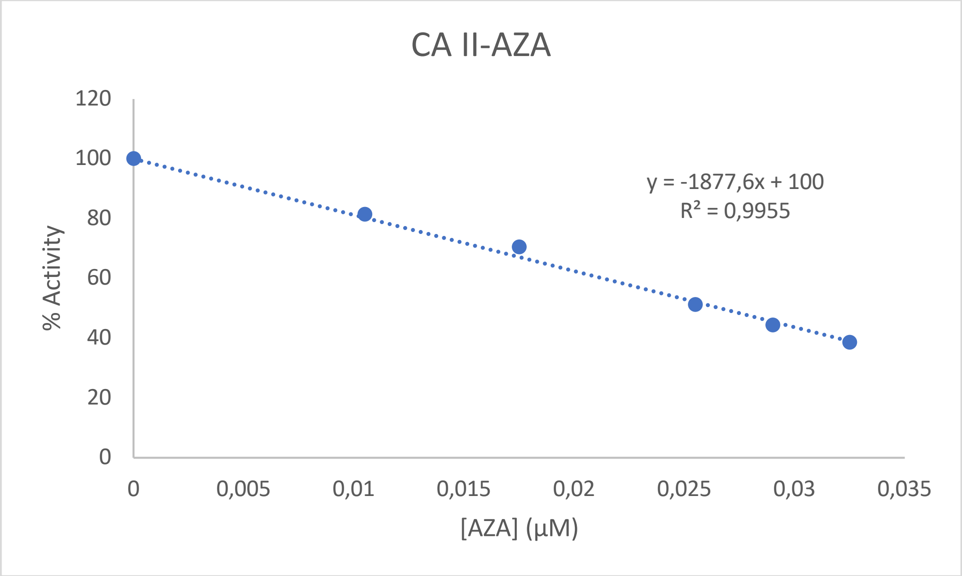

IC_{50} graph of compound **1** with AChE enzyme

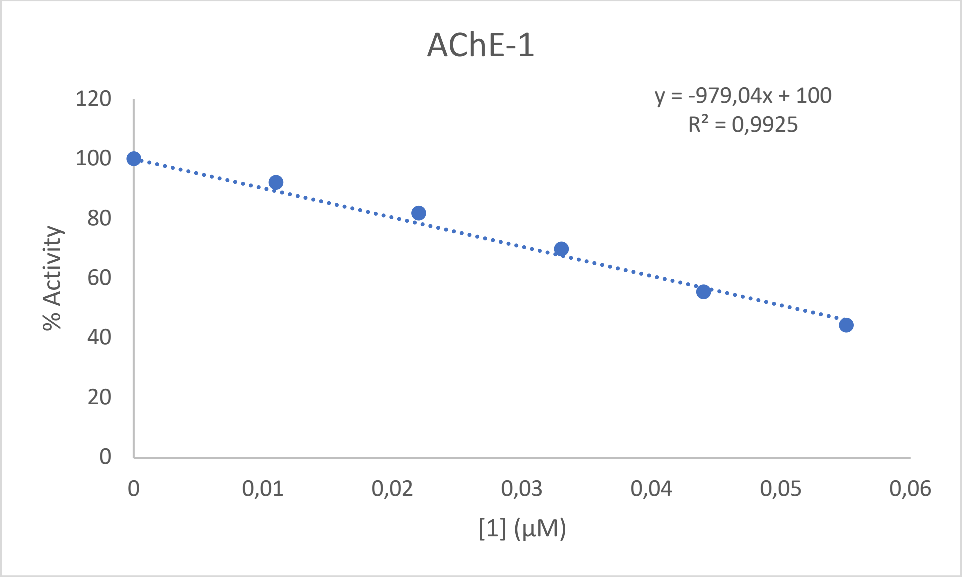

IC_{50} graph of compound **2** with AChE enzyme

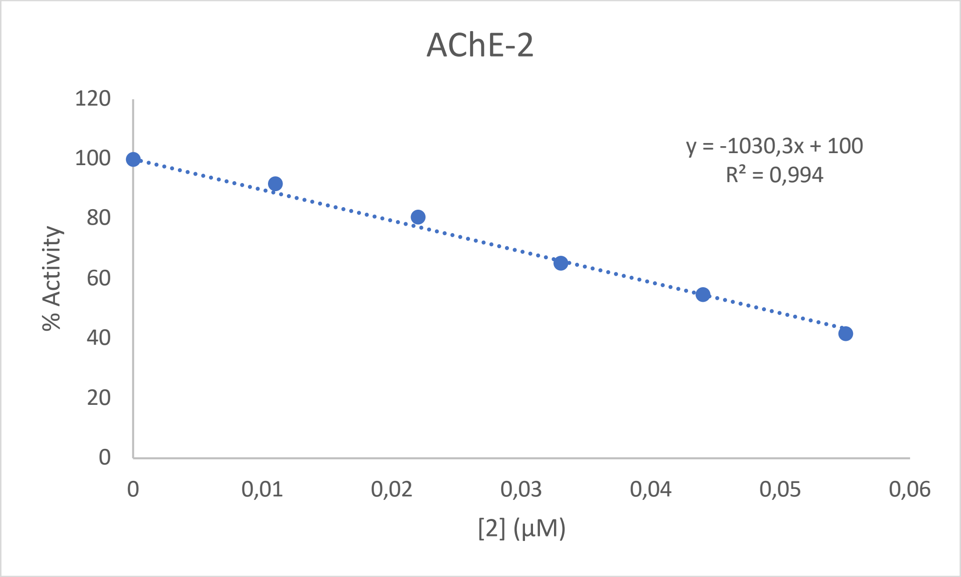

IC$_{50}$ graph of compound **3** with AChE enzyme

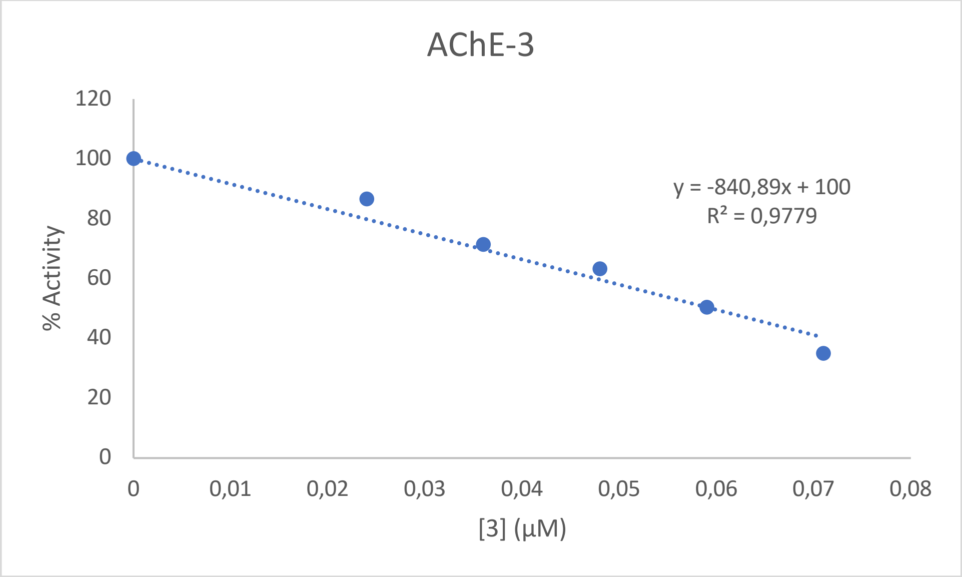

IC$_{50}$ graph of compound **4** with AChE enzyme

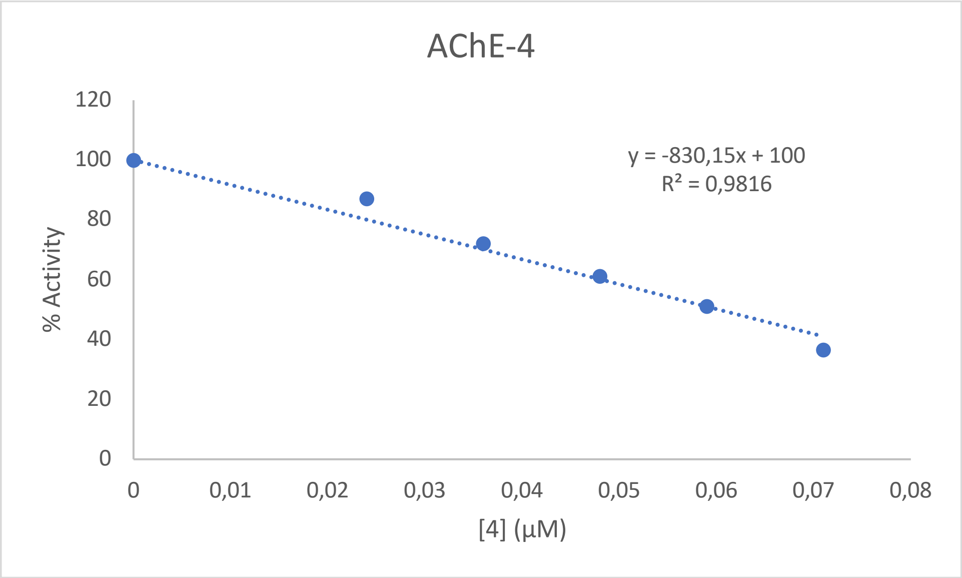

IC$_{50}$ graph of compound **5** with AChE enzyme

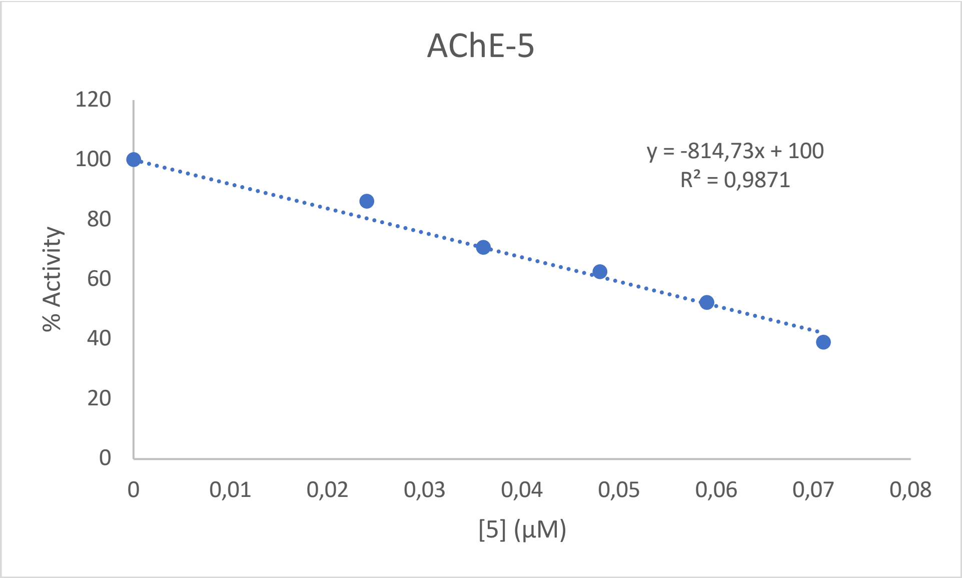

IC$_{50}$ graph of compound **6** with AChE enzyme

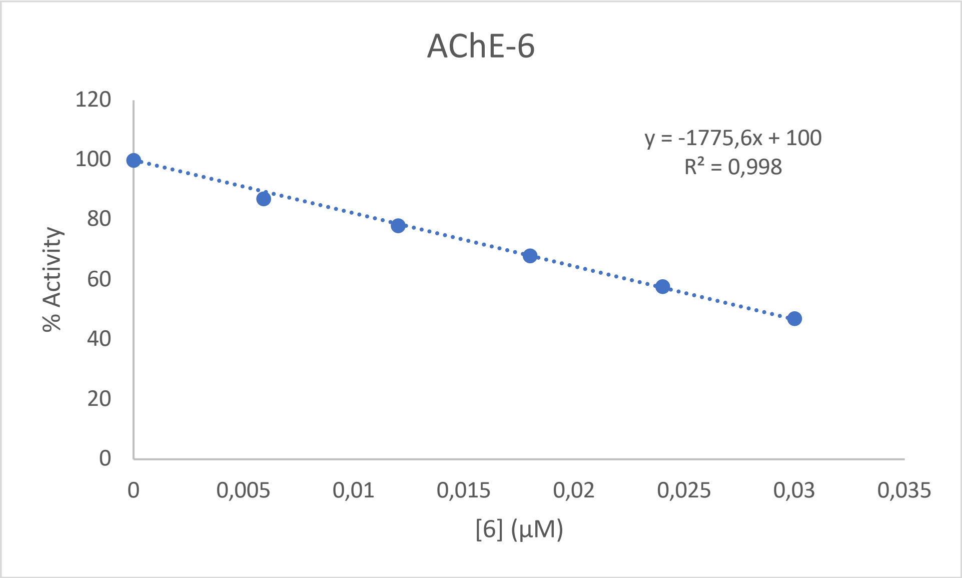

IC$_{50}$ graph of compound **7** with AChE enzyme

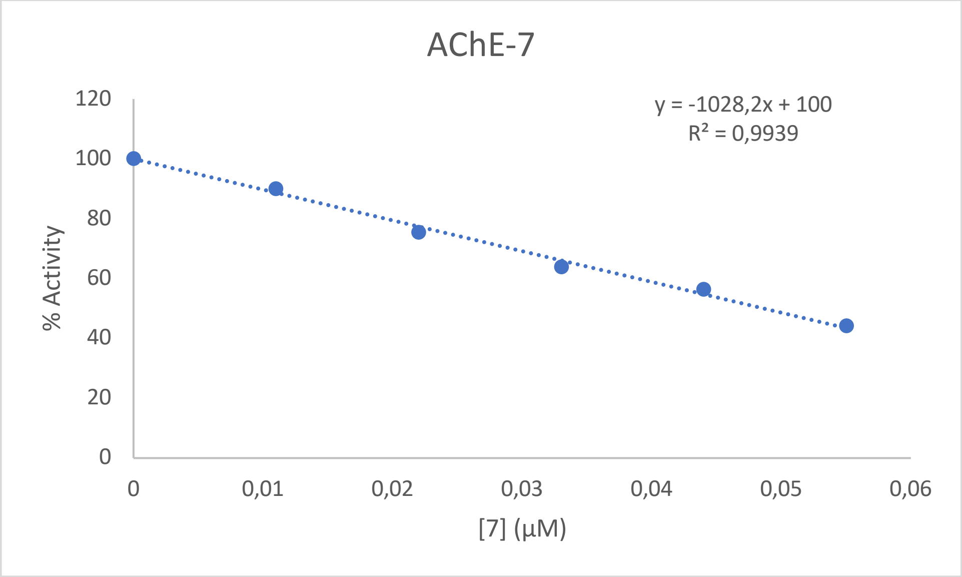

IC$_{50}$ graph of compound **8** with AChE enzyme

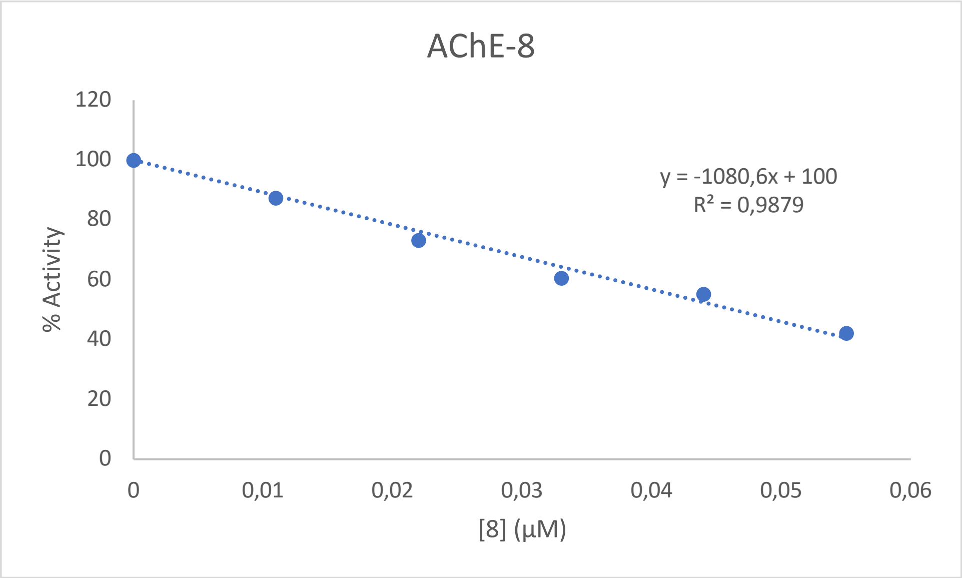

IC$_{50}$ graph of compound **9** with AChE enzyme

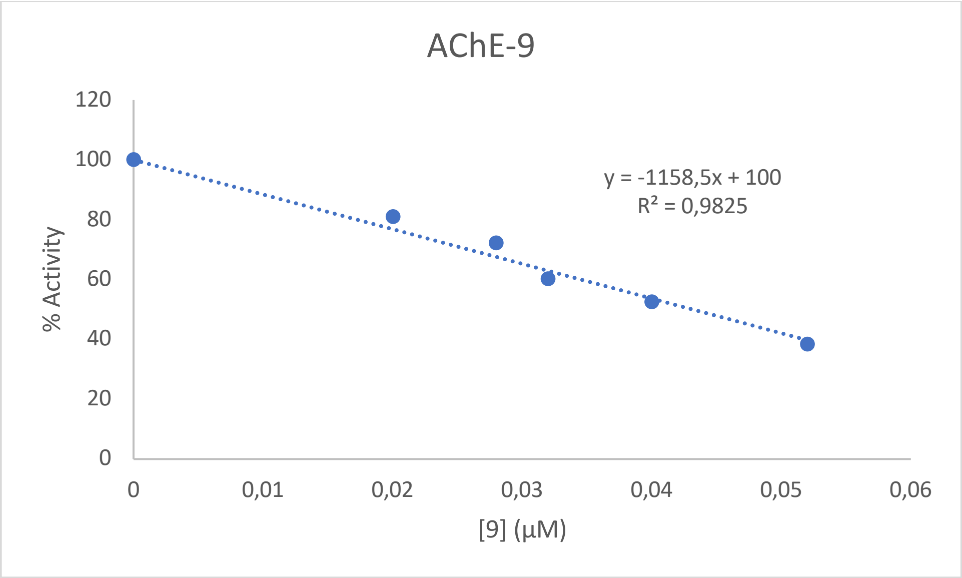

IC$_{50}$ graph of compound **10** with AChE enzyme

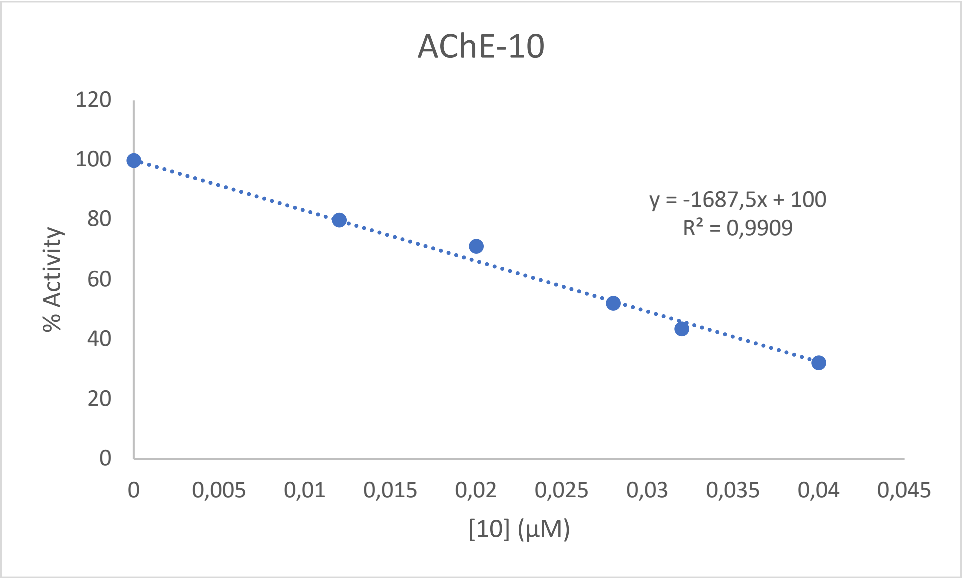

IC$_{50}$ graph of compound **11** with AChE enzyme

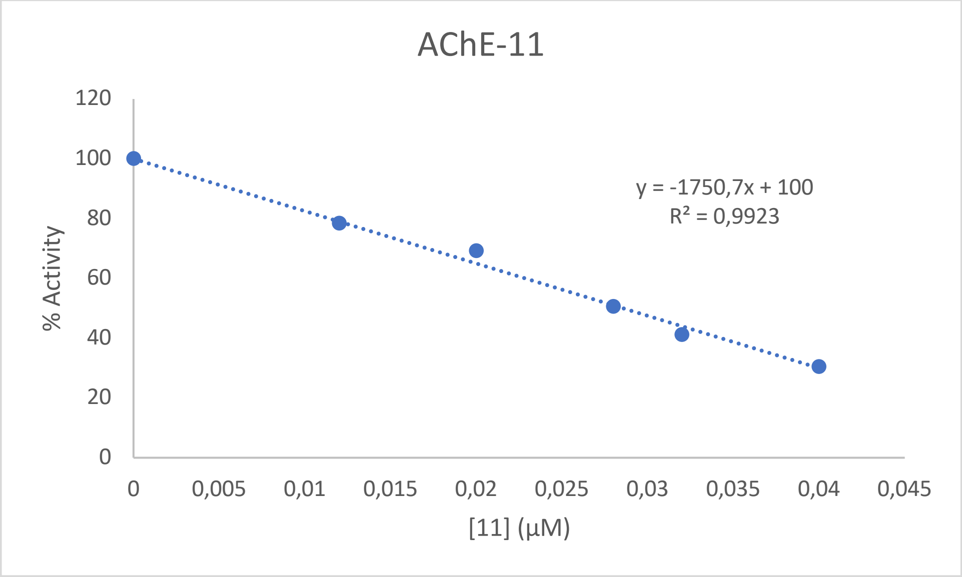

IC$_{50}$ graph of compound **12** with AChE enzyme

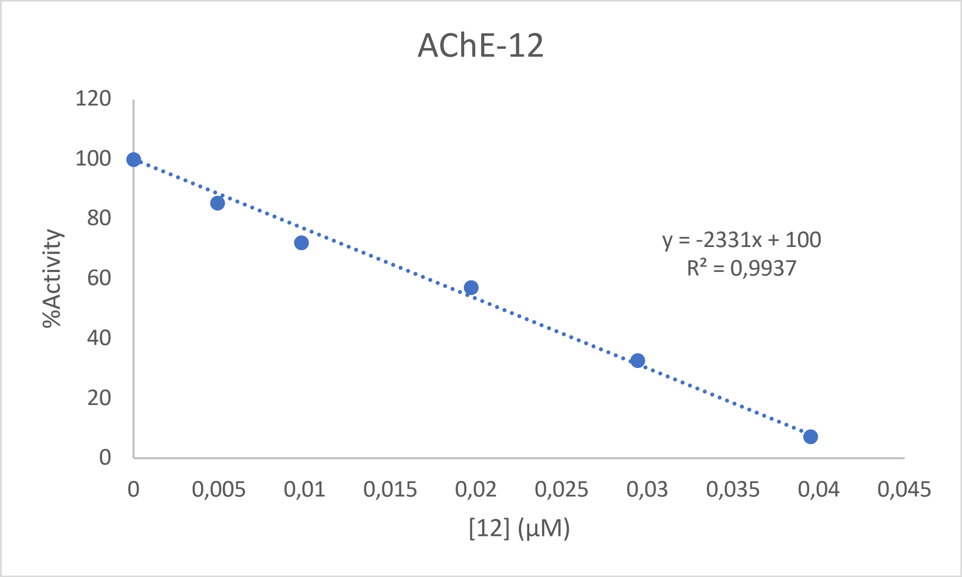

IC$_{50}$ graph of neostigmine with AChE enzyme

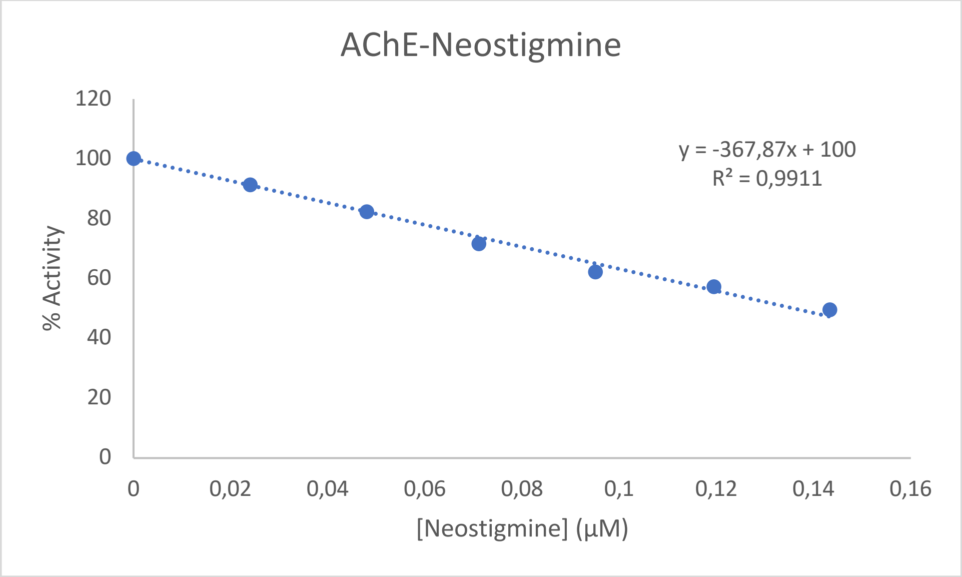

IC$_{50}$ graph of rivastigmine with AChE enzyme

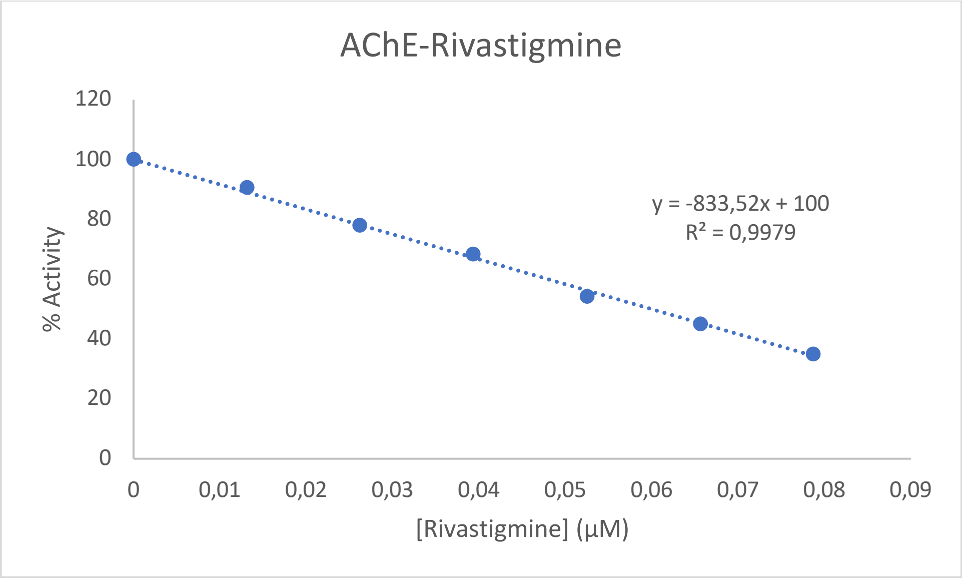

IC$_{50}$ graph of compound **1** with BChE enzyme

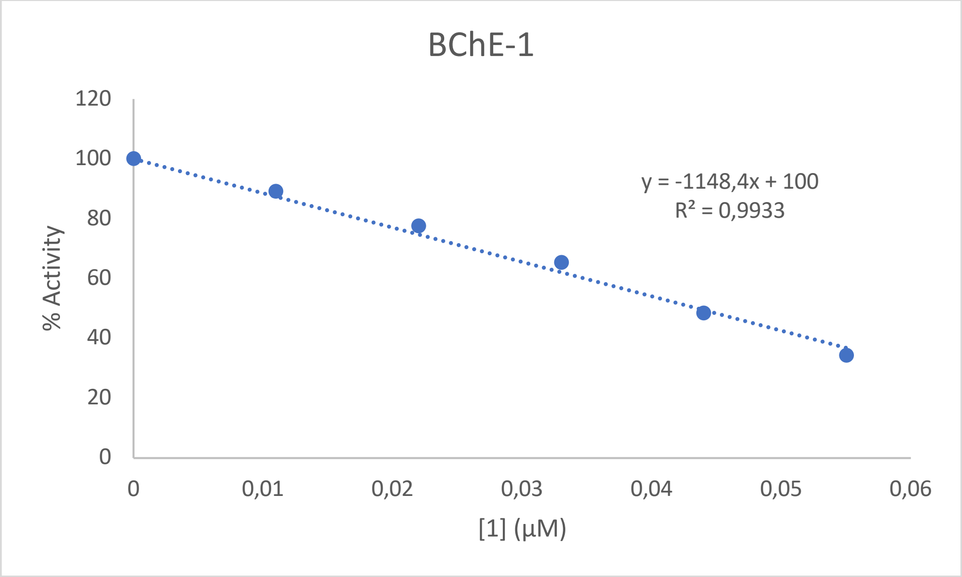

IC$_{50}$ graph of compound **2** with BChE enzyme

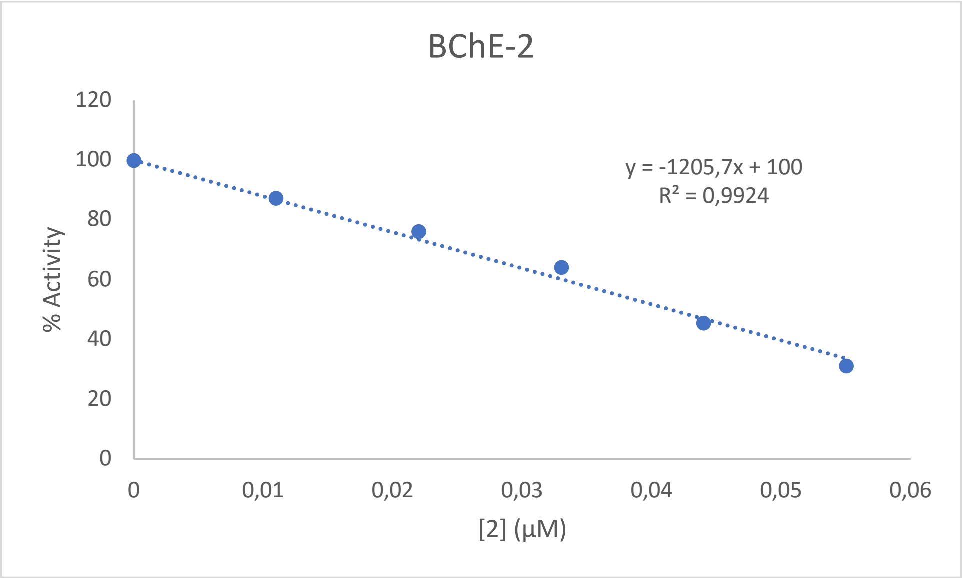

IC_{50} graph of compound **3** with BChE enzyme

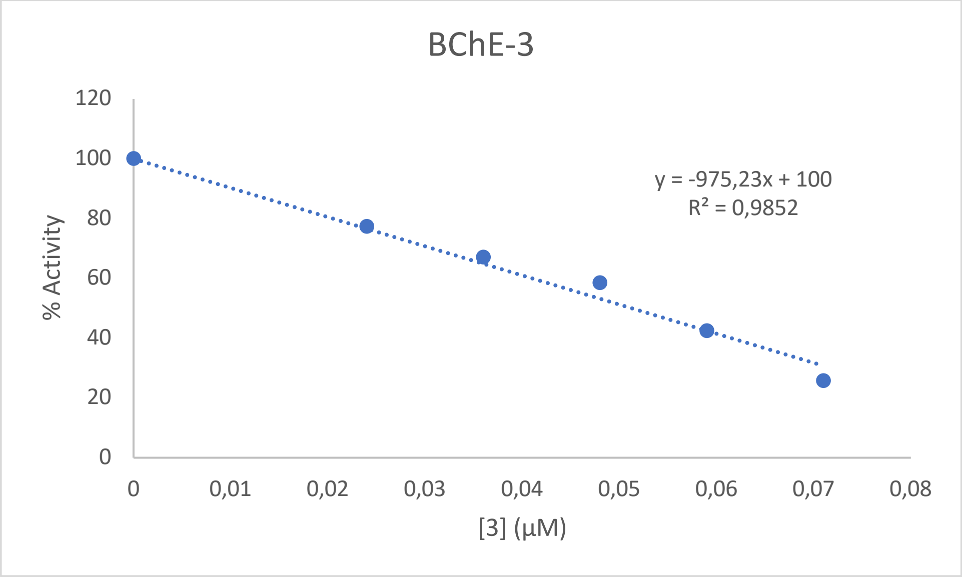

IC_{50} graph of compound **4** with BChE enzyme

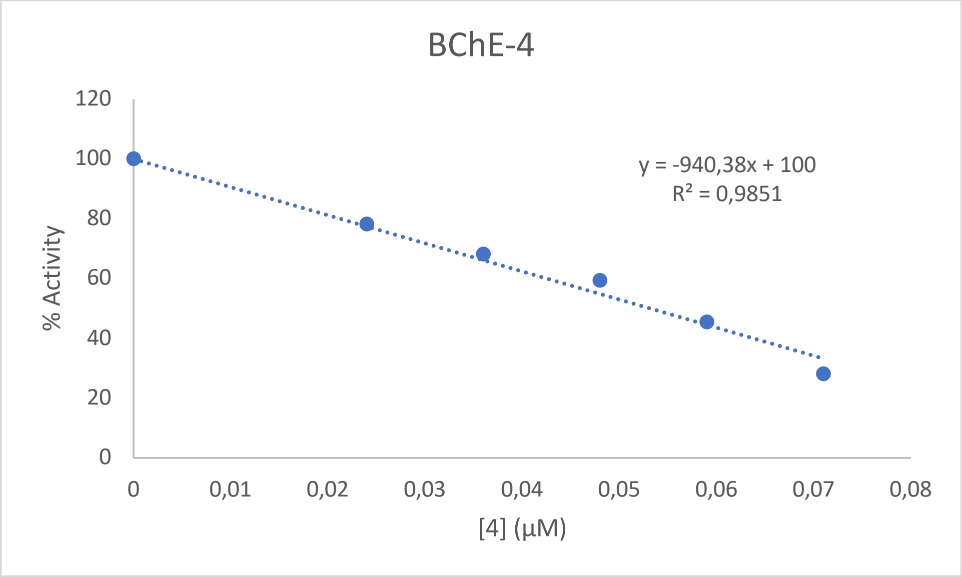

IC$_{50}$ graph of compound **5** with BChE enzyme

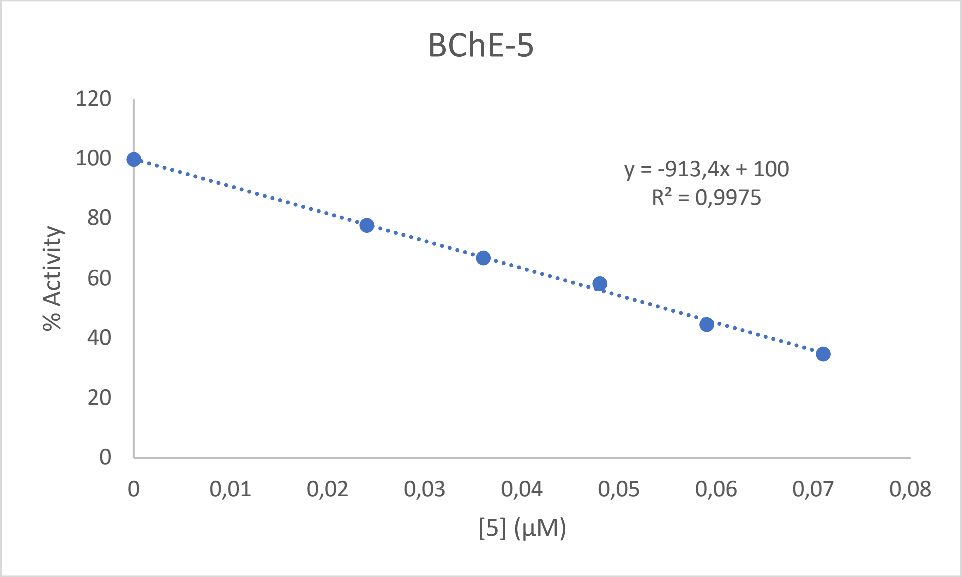

IC$_{50}$ graph of compound **6** with BChE enzyme

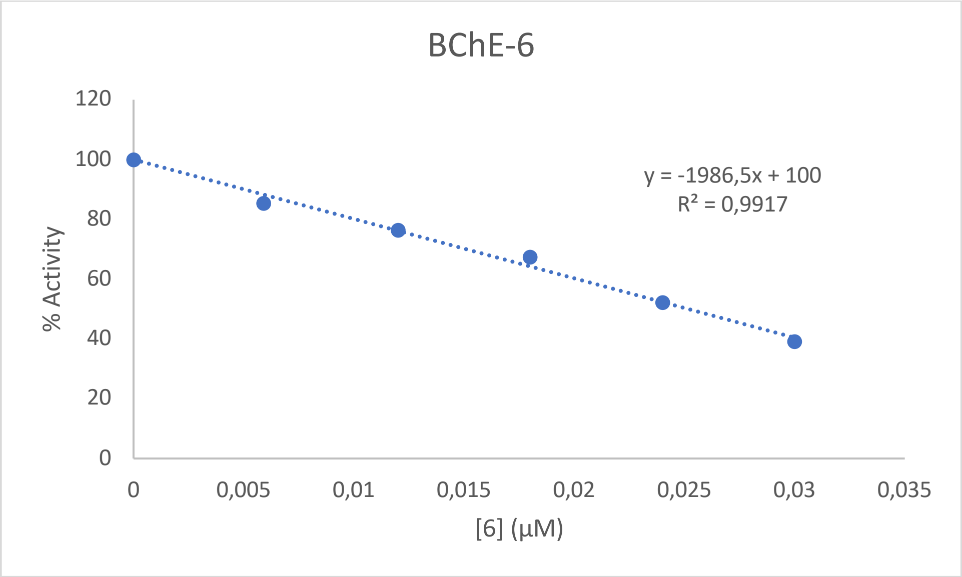

IC_{50} graph of compound **7** with BChE enzyme

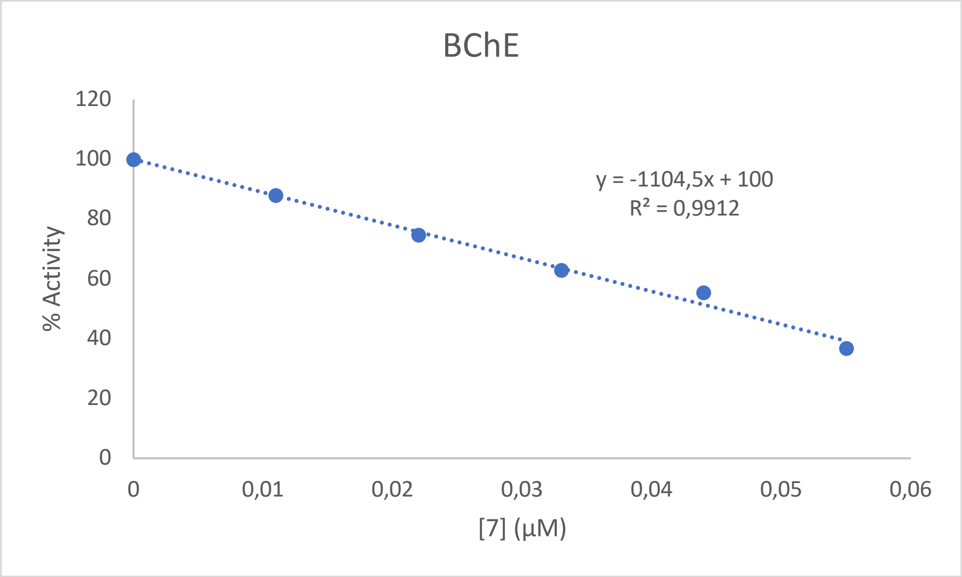

IC_{50} graph of compound **8** with BChE enzyme

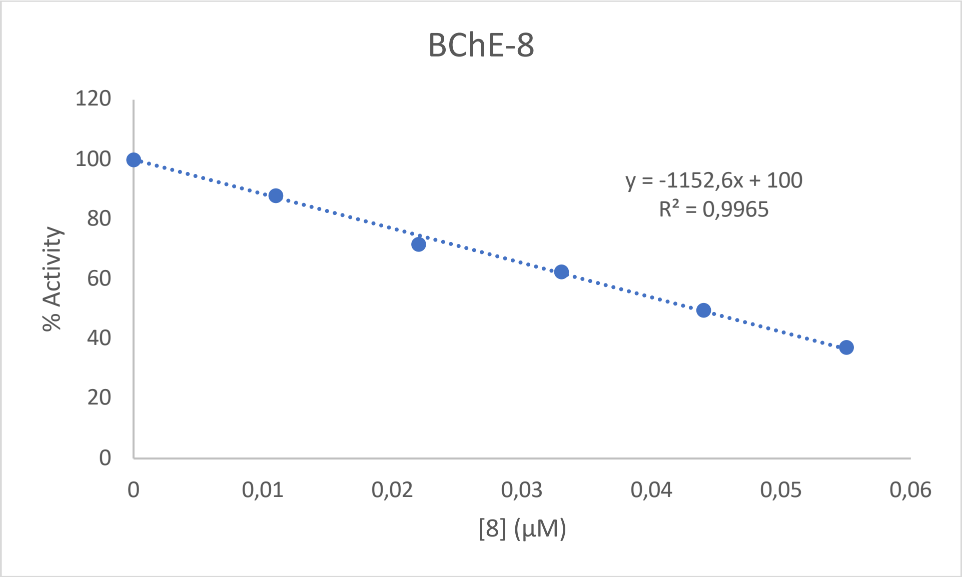

IC$_{50}$ graph of compound **9** with BChE enzyme

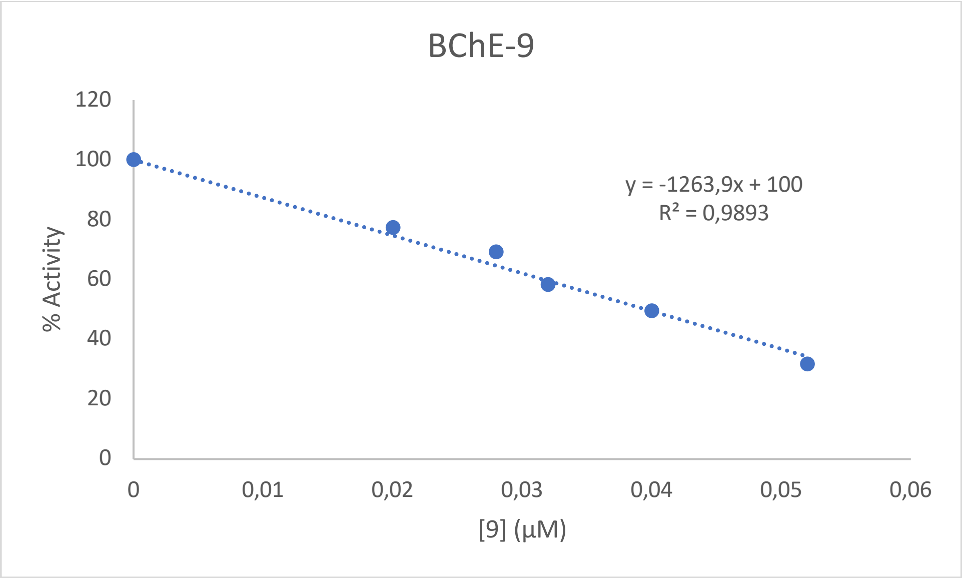

IC$_{50}$ graph of compound **10** with BChE enzyme

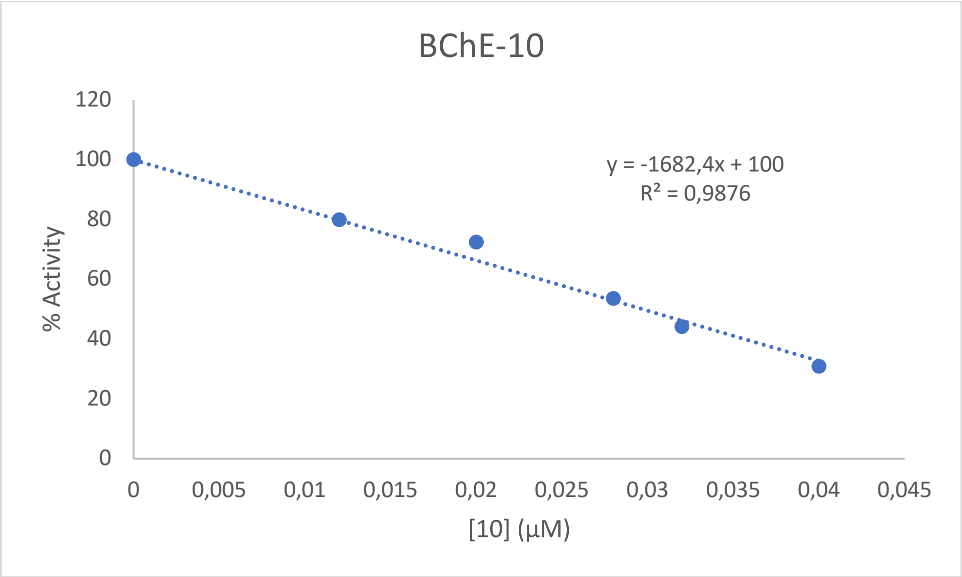

IC_{50} graph of compound **11** with BChE enzyme

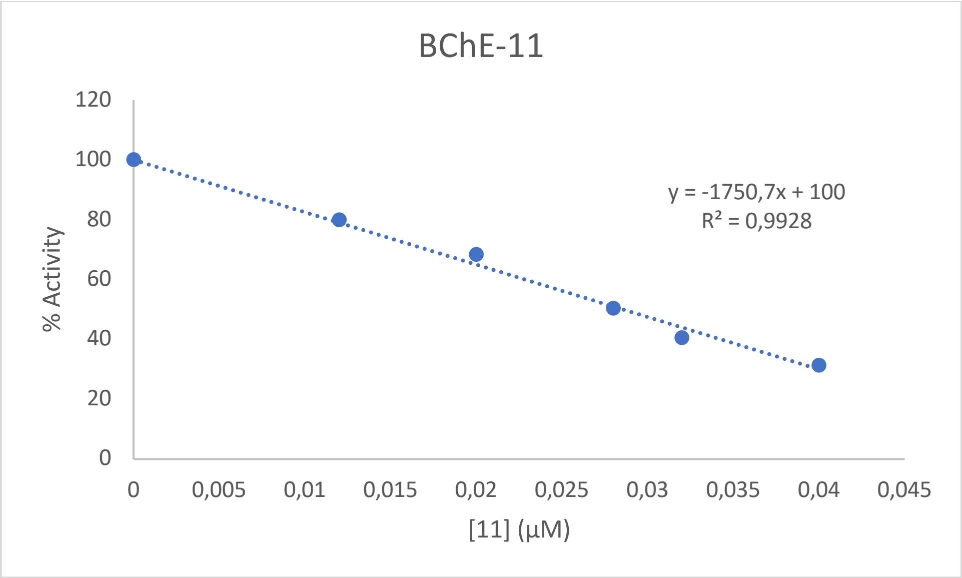

IC_{50} graph of compound **12** with BChE enzyme

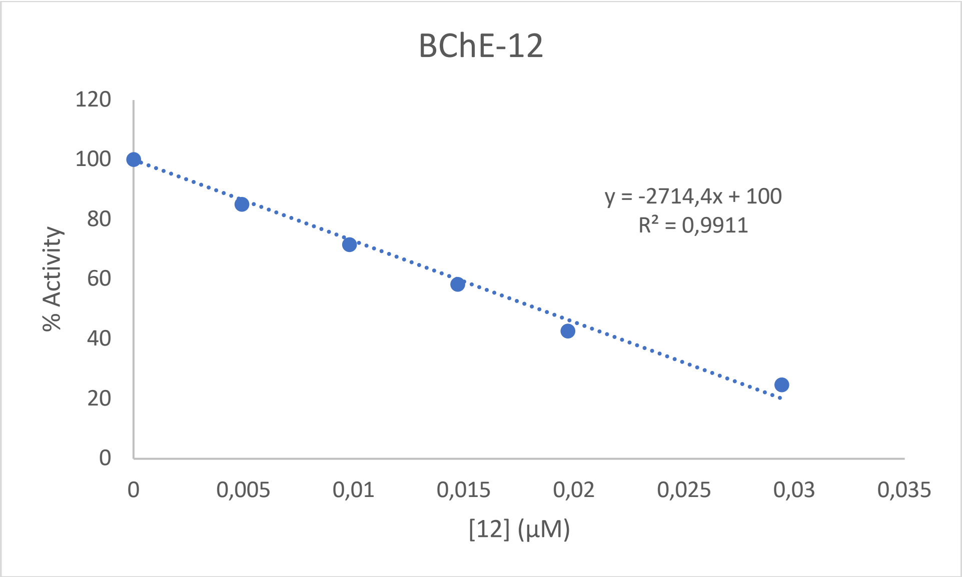

IC$_{50}$ graph of neostigmine with BChE enzyme

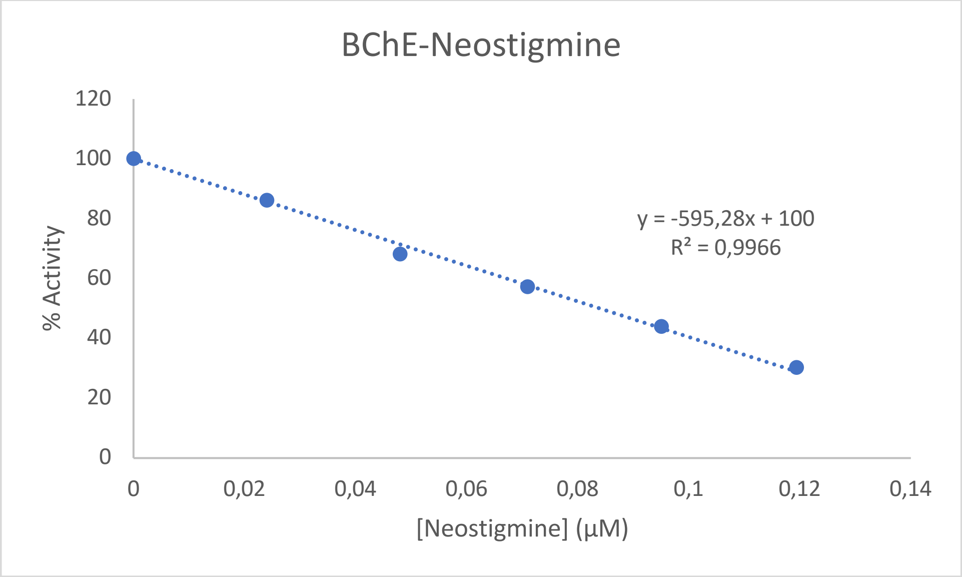

IC$_{50}$ graph of rivastigmine with BChE enzyme

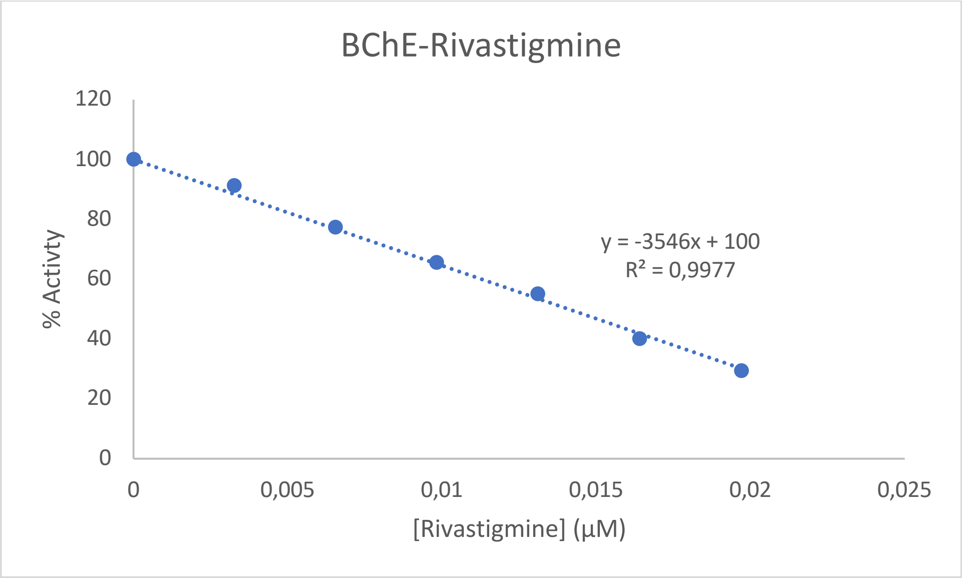

Table S1. 2D structures of top-docking scored compounds of analogs of **12** at the binding pocket of AChE. Tanimato coefficients of compounds were also listed.

**Compound**	**2D Structure**	**Tanimoto Coefficient**	**AChE IFD docking score (kcal/mol)**
**12**	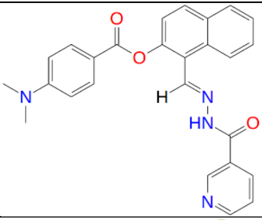		-13.75
**ZINC000021092702**	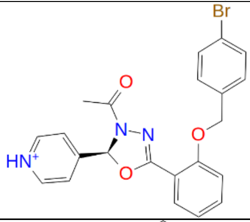	0.56	-15.80
**ZINC000009529740**	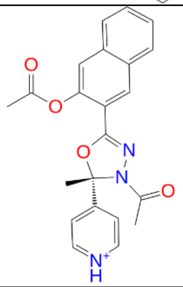	0.68	-15.54
**ZINC000021941257**	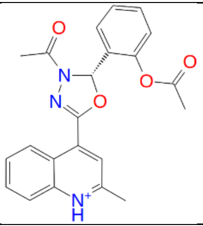	0.50	-15.07
**ZINC000021941392**	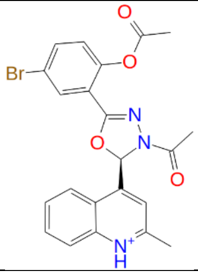	0.52	-14.37
**ZINC000006630439**	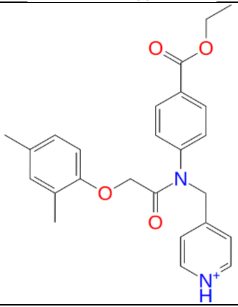	0.53	-14.37

**ZINC000006630441**	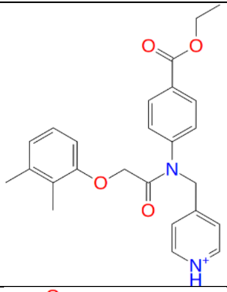	0.51	-14.31
**ZINC000006673191**	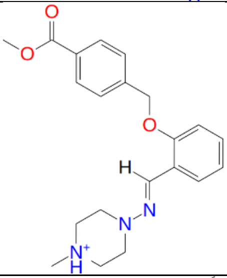	0.50	-14.05
**ZINC000006630440**	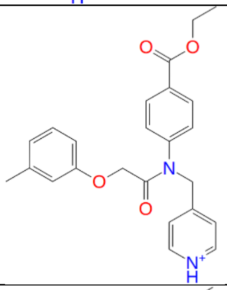	0.51	-14.02
**ZINC000017094146**	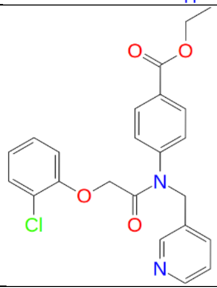	0.61	-13.98
**ZINC000032923359**	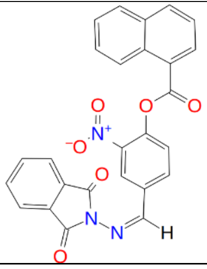	0.66	-13.96

Table S2. Average MM/GBSA score of **12** and its top-scored analog at the binding site of AChE.

**Compound**	**2D Structure**	**Average MM/GBSA score (kcal/mol) AChE**

**12**	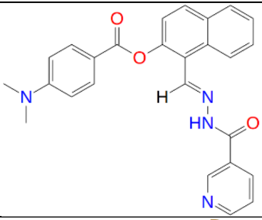	-80.56±8.46
**ZINC000021092702**	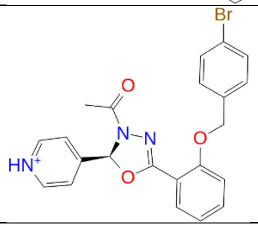	-93.32±13.81

Table S3. 2D structures of top-docking scored compounds of analogs of **12** at the binding pocket of BChE. Tanimato coefficients of compounds were also listed.

**Compound**	**2D Structure**	**Tanimoto Coefficient**	**BChE IFD docking score (kcal/mol)**
**12**	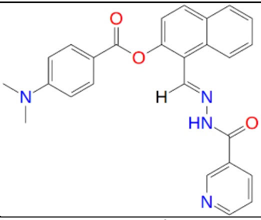		-10.39
**ZINC000254442184**	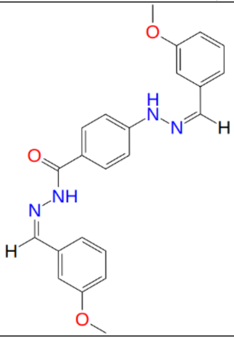	0.57	-12.15
**ZINC000010313155**	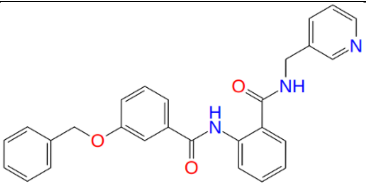	0.51	-11.75
**ZINC000009529721**	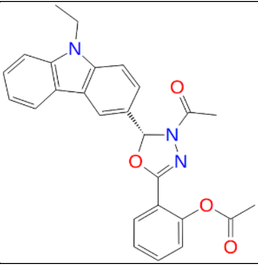	0.60	-11.63

**ZINC000409351001**	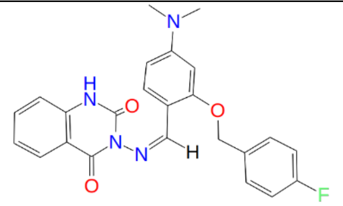	0.51	-11.62
**ZINC000059417082**	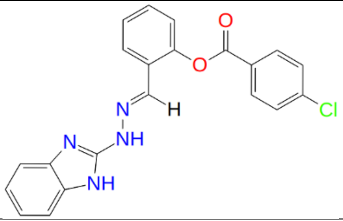	0.70	-11.17
**ZINC000033021451**	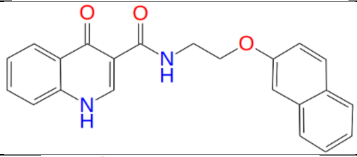	0.51	-10.98
**ZINC000059464265**	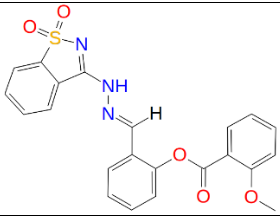	0.54	-10.91
**ZINC000006630389**	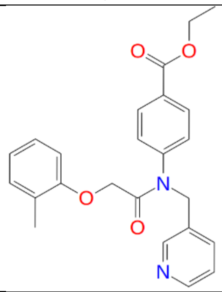	0.70	-10.91
**ZINC000064567857**	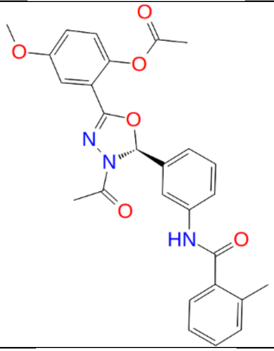	0.58	-10.76
**ZINC000064567862**	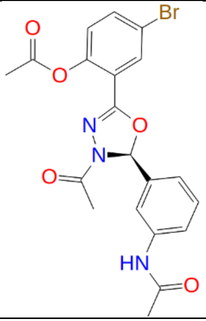	0.50	-10.58

**ZINC000002543792**	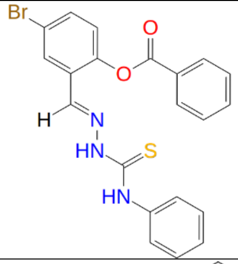	0.61	-10.56
**ZINC000020762720**	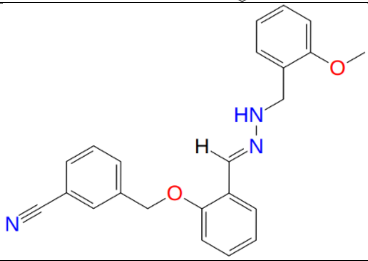	0.67	-10.41
**ZINC000001823120**	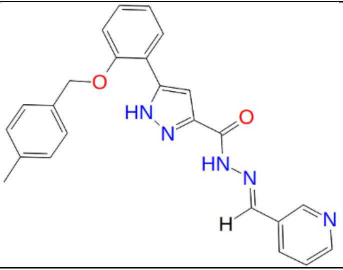	0.67	-10.41

Table S4. Average MM/GBSA score of **12** and its top-scored analog at the binding site of BChE.

**Compound**	**2D Structure**	**Average MM/GBSA (kcal/mol) BChE**
**12**	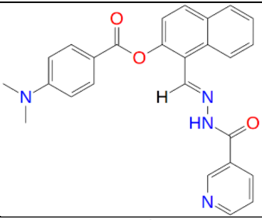	-76.89±12.79
**ZINC000254442184**	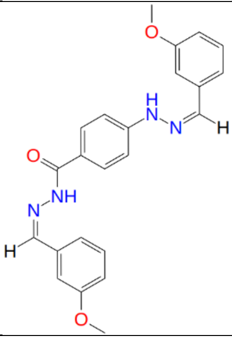	-90.18±9.82

Table S5. 2D structures of top-docking scored compounds of analogs of **8** at the binding pocket of hCA I. Tanimato coefficients of compounds were also listed.

Compound	2D Structure	Tanimoto Coefficient	IFD hCA I (kcal/mol)
**8**	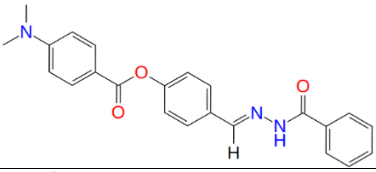		-6.75
**ZINC000020196504**	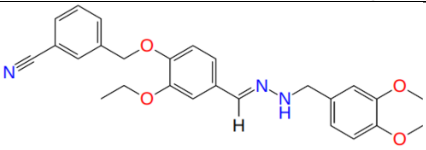	0.72	-8.66
**ZINC000020196502**	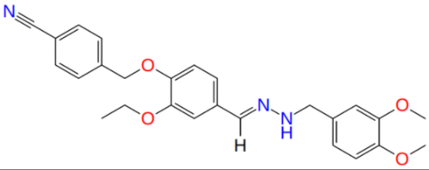	0.73	-8.65
**ZINC000020195736**	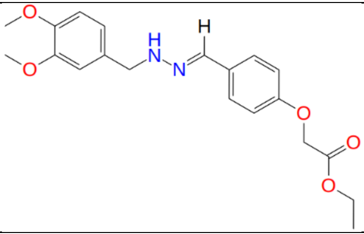	0.61	-8.49
**ZINC000025919802**	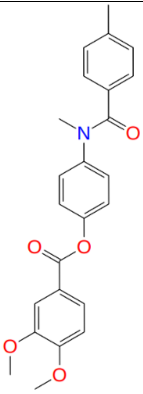	0.64	-8.40
**ZINC000095983045**	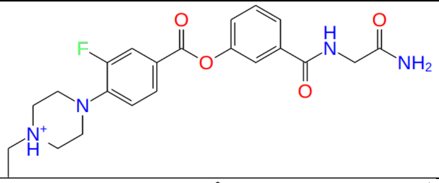	0.55	-8.38
**ZINC000020762929**	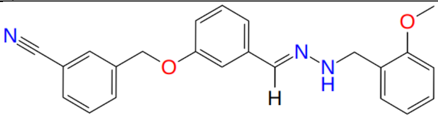	0.65	-8.29
**ZINC000000851910**	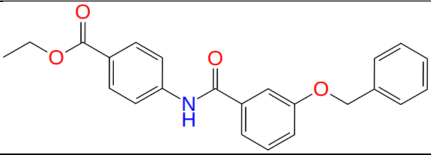	0.65	-8.18

**ZINC000007668590**	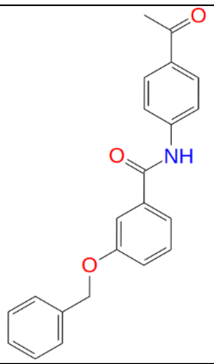	0.59	-8.17
**ZINC000000642738**	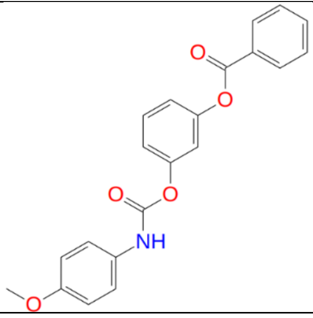	0.58	-8.14

Table S6. Average MM/GBSA score of **8** and its top-scored analog at the binding site of hCA I.

**Compound**	**2D Structure**	**Average MM/GBSA (kcal/mol) hCA I**
**8**	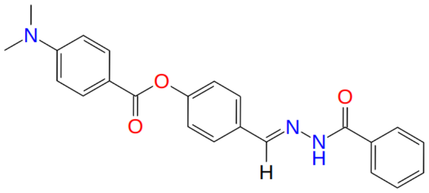	-34.55±4.71
**ZINC000020196504**	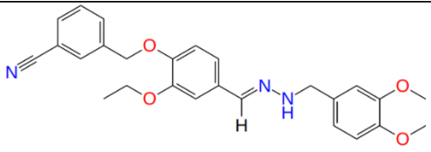	-53.32±8.48

Table S7. 2D structures of top-docking scored compounds of analogs of **8** at the binding pocket of hCA II. Tanimato coefficients of compounds were also listed.

Compound	2D Structure	Tanimoto Coefficient	IFD hCA II (kcal/mol)
**8**	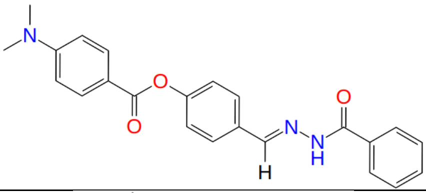		-6.03
**ZINC000003528364**	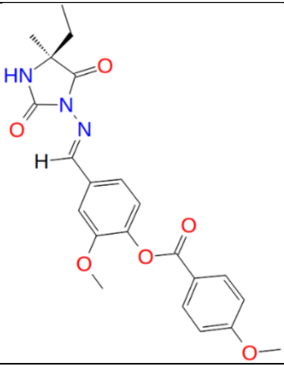	0.55	-9.51
**ZINC000003097389**	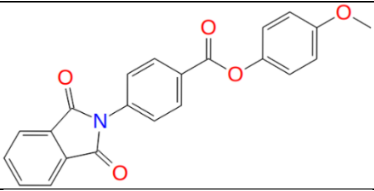	0.67	-9.42
**ZINC000020762990**	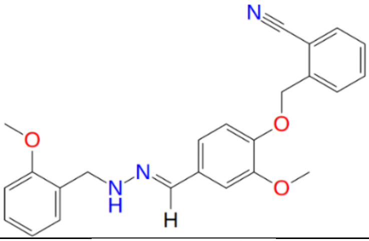	0.58	-9.22
**ZINC000008754736**	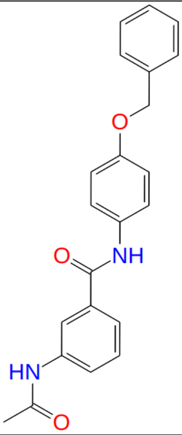	0.58	-8.99

**ZINC000004672224**	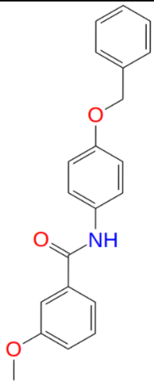	0.53	-8.92
**ZINC000010119042**	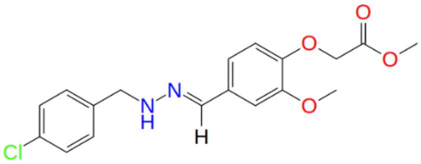	0.61	-8.91
**ZINC000001750522**	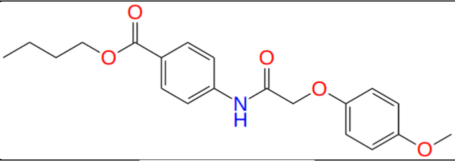	0.59	-8.81
**ZINC000004841955**	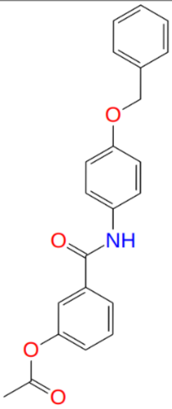	0.71	-8.79
**ZINC000000678730**	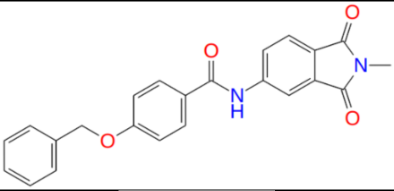	0.53	-8.77
**ZINC000000904112**	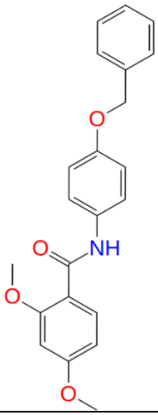	0.58	-8.73

Table S8. Average MM/GBSA score of **8** and its top-scored analog at the binding site of hCA II.

Compound	2D Structure	Average MM/GBSA (kcal/mol) hCA II
**8**	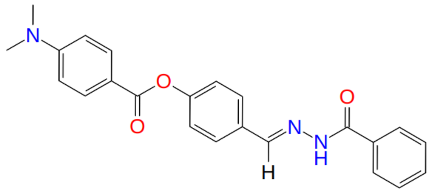	-37.65±8.73
**ZINC000003528364**	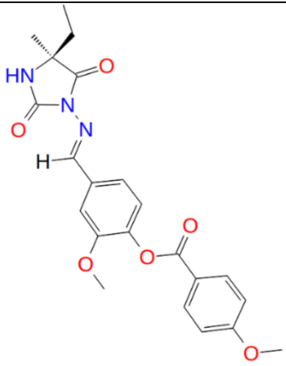	-39.81±7.99

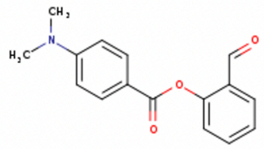

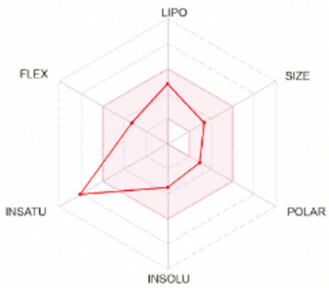

SMILES O=Cc1ccccc1OC(=O)c1ccc(cc1)N(C)C

Physicochemical Properties	
Formula	C16H15NO3
Molecular weight	269.30 g/mol
Num. heavy atoms	20
Num. arom. heavy atoms	12
Fraction Csp3	0.12
Num. rotatable bonds	5
Num. H-bond acceptors	3
Num. H-bond donors	0
Molar Refractivity	77.44
TPSA	46.61 Å²

Lipophilicity	
Log $P_{o/w}$ (iLOGP)	2.57
Log $P_{o/w}$ (XLOGP3)	2.92
Log $P_{o/w}$ (WLOGP)	2.78
Log $P_{o/w}$ (MLOGP)	2.65
Log $P_{o/w}$ (SILICOS-IT)	2.74
Consensus Log $P_{o/w}$	2.73

Water Solubility	
Log *S* (ESOL)	-3.46
Solubility	9.27e-02 mg/ml ; 3.44e-04 mol/l
Class	Soluble
Log *S* (Ali)	-3.56
Solubility	7.42e-02 mg/ml ; 2.75e-04 mol/l
Class	Soluble
Log *S* (SILICOS-IT)	-4.66
Solubility	5.84e-03 mg/ml ; 2.17e-05 mol/l
Class	Moderately soluble

Pharmacokinetics	
GI absorption	High
BBB permeant	Yes
P-gp substrate	No
CYP1A2 inhibitor	Yes
CYP2C19 inhibitor	Yes
CYP2C9 inhibitor	No
CYP2D6 inhibitor	No
CYP3A4 inhibitor	No
Log K_p (skin permeation)	-5.87 cm/s

Druglikeness	
Lipinski	Yes; 0 violation
Ghose	Yes
Veber	Yes
Egan	Yes
Muegge	Yes
Bioavailability Score	0.55

Medicinal Chemistry	
PAINS	0 alert
Brenk	2 alerts: aldehyde, phenol_ester
Leadlikeness	Yes
Synthetic accessibility	2.06

Figure S1. ADME and physicochemical properties of compound **1**.

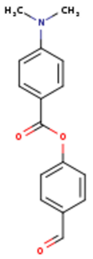

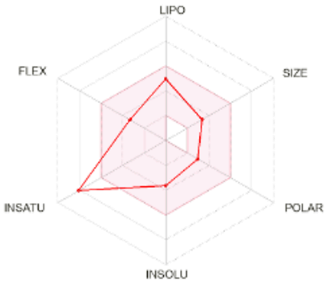

SMILES O=Cc1ccc(cc1)OC(=O)c1ccc(cc1)N(C)C

Physicochemical Properties	
Formula	C16H15NO3
Molecular weight	269.30 g/mol
Num. heavy atoms	20
Num. arom. heavy atoms	12
Fraction Csp3	0.12
Num. rotatable bonds	5
Num. H-bond acceptors	3
Num. H-bond donors	0
Molar Refractivity	77.44
TPSA	46.61 Å²

Lipophilicity	
Log $P_{o/w}$ (iLOGP)	2.30
Log $P_{o/w}$ (XLOGP3)	3.18
Log $P_{o/w}$ (WLOGP)	2.78
Log $P_{o/w}$ (MLOGP)	2.65
Log $P_{o/w}$ (SILICOS-IT)	2.74
Consensus Log $P_{o/w}$	2.73

Water Solubility	
Log *S* (ESOL)	-3.63
Solubility	6.36e-02 mg/ml ; 2.36e-04 mol/l
Class	Soluble
Log *S* (Ali)	-3.83
Solubility	3.98e-02 mg/ml ; 1.48e-04 mol/l
Class	Soluble
Log *S* (SILICOS-IT)	-4.66
Solubility	5.84e-03 mg/ml ; 2.17e-05 mol/l
Class	Moderately soluble

Pharmacokinetics	
GI absorption	High
BBB permeant	Yes
P-gp substrate	No
CYP1A2 inhibitor	Yes
CYP2C19 inhibitor	Yes
CYP2C9 inhibitor	No
CYP2D6 inhibitor	No
CYP3A4 inhibitor	No
Log K_p (skin permeation)	-5.68 cm/s

Druglikeness	
Lipinski	Yes; 0 violation
Ghose	Yes
Veber	Yes
Egan	Yes
Muegge	Yes
Bioavailability Score	0.55

Medicinal Chemistry	
PAINS	0 alert
Brenk	2 alerts: aldehyde, phenol_ester
Leadlikeness	Yes
Synthetic accessibility	1.92

Figure S2. ADME and physicochemical properties of compound **2**.

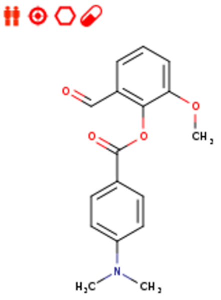

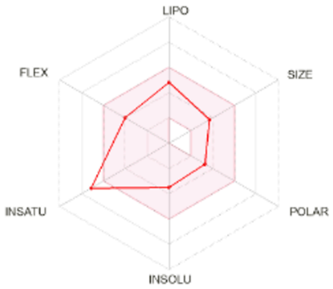

SMILES COc1cccc(c1OC(=O)c1ccc(cc1)N(C)C)C=O

Physicochemical Properties	
Formula	C17H17NO4
Molecular weight	299.32 g/mol
Num. heavy atoms	22
Num. arom. heavy atoms	12
Fraction Csp3	0.18
Num. rotatable bonds	6
Num. H-bond acceptors	4
Num. H-bond donors	0
Molar Refractivity	83.93
TPSA	55.84 Å^2

Lipophilicity	
Log $P_{o/w}$ (iLOGP)	2.67
Log $P_{o/w}$ (XLOGP3)	2.89
Log $P_{o/w}$ (WLOGP)	2.79
Log $P_{o/w}$ (MLOGP)	2.33
Log $P_{o/w}$ (SILICOS-IT)	2.80
Consensus Log $P_{o/w}$	2.70

Water Solubility	
Log *S* (ESOL)	-3.52
Solubility	8.95e-02 mg/ml ; 2.99e-04 mol/l
Class	Soluble
Log *S* (Ali)	-3.72
Solubility	5.67e-02 mg/ml ; 1.89e-04 mol/l
Class	Soluble
Log *S* (SILICOS-IT)	-4.78
Solubility	4.97e-03 mg/ml ; 1.66e-05 mol/l
Class	Moderately soluble

Pharmacokinetics	
GI absorption	High
BBB permeant	Yes
P-gp substrate	No
CYP1A2 inhibitor	Yes
CYP2C19 inhibitor	Yes
CYP2C9 inhibitor	No
CYP2D6 inhibitor	No
CYP3A4 inhibitor	Yes
Log K_p (skin permeation)	-6.07 cm/s

Druglikeness	
Lipinski	Yes; 0 violation
Ghose	Yes
Veber	Yes
Egan	Yes
Muegge	Yes
Bioavailability Score	0.55

Medicinal Chemistry	
PAINS	0 alert
Brenk	2 alerts: aldehyde, phenol_ester
Leadlikeness	Yes
Synthetic accessibility	2.36

Figure S3. ADME and physicochemical properties of compound **3**.

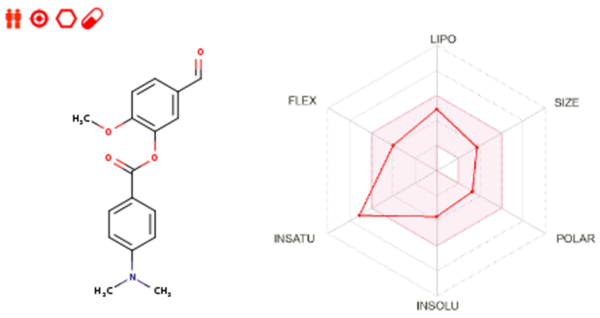

SMILES COc1ccc(cc1OC(=O)c1ccc(cc1)N(C)C)C=O

Physicochemical Properties	
Formula	C17H17NO4
Molecular weight	299.32 g/mol
Num. heavy atoms	22
Num. arom. heavy atoms	12
Fraction Csp3	0.18
Num. rotatable bonds	6
Num. H-bond acceptors	4
Num. H-bond donors	0
Molar Refractivity	83.93
TPSA	55.84 Å²

Lipophilicity	
Log $P_{o/w}$ (iLOGP)	2.75
Log $P_{o/w}$ (XLOGP3)	3.10
Log $P_{o/w}$ (WLOGP)	2.79
Log $P_{o/w}$ (MLOGP)	2.33
Log $P_{o/w}$ (SILICOS-IT)	2.80
Consensus Log $P_{o/w}$	2.75

Water Solubility	
Log *S* (ESOL)	-3.66
Solubility	6.60e-02 mg/ml ; 2.21e-04 mol/l
Class	Soluble
Log *S* (Ali)	-3.94
Solubility	3.43e-02 mg/ml ; 1.15e-04 mol/l
Class	Soluble
Log *S* (SILICOS-IT)	-4.78
Solubility	4.97e-03 mg/ml ; 1.66e-05 mol/l
Class	Moderately soluble

Pharmacokinetics	
GI absorption	High
BBB permeant	Yes
P-gp substrate	No
CYP1A2 inhibitor	Yes
CYP2C19 inhibitor	Yes
CYP2C9 inhibitor	No
CYP2D6 inhibitor	No
CYP3A4 inhibitor	Yes
Log K_p (skin permeation)	-5.92 cm/s

Druglikeness	
Lipinski	Yes; 0 violation
Ghose	Yes
Veber	Yes
Egan	Yes
Muegge	Yes
Bioavailability Score	0.55

Medicinal Chemistry	
PAINS	0 alert
Brenk	2 alerts: aldehyde, phenol_ester
Leadlikeness	Yes
Synthetic accessibility	2.32

Figure S4. ADME and physicochemical properties of compound **4**.

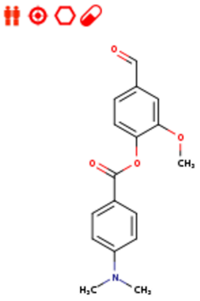

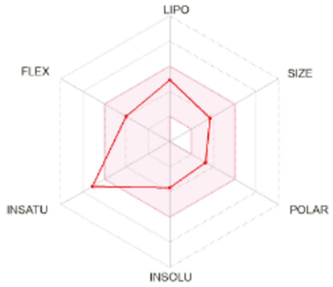

SMILES O=Cc1ccc(c(c1)OC)OC(=O)c1ccc(cc1)N(C)C

Physicochemical Properties	
Formula	C17H17NO4
Molecular weight	299.32 g/mol
Num. heavy atoms	22
Num. arom. heavy atoms	12
Fraction Csp3	0.18
Num. rotatable bonds	6
Num. H-bond acceptors	4
Num. H-bond donors	0
Molar Refractivity	83.93
TPSA	55.84 Å^2

Lipophilicity	
Log $P_{o/w}$ (iLOGP)	2.67
Log $P_{o/w}$ (XLOGP3)	3.10
Log $P_{o/w}$ (WLOGP)	2.79
Log $P_{o/w}$ (MLOGP)	2.33
Log $P_{o/w}$ (SILICOS-IT)	2.80
Consensus Log $P_{o/w}$	2.74

Water Solubility	
Log *S* (ESOL)	-3.66
Solubility	6.60e-02 mg/ml ; 2.21e-04 mol/l
Class	Soluble
Log *S* (Ali)	-3.94
Solubility	3.43e-02 mg/ml ; 1.15e-04 mol/l
Class	Soluble
Log *S* (SILICOS-IT)	-4.78
Solubility	4.97e-03 mg/ml ; 1.66e-05 mol/l
Class	Moderately soluble

Pharmacokinetics	
GI absorption	High
BBB permeant	Yes
P-gp substrate	No
CYP1A2 inhibitor	Yes
CYP2C19 inhibitor	Yes
CYP2C9 inhibitor	No
CYP2D6 inhibitor	No
CYP3A4 inhibitor	No
Log K_p (skin permeation)	-5.92 cm/s

Druglikeness	
Lipinski	Yes; 0 violation
Ghose	Yes
Veber	Yes
Egan	Yes
Muegge	Yes
Bioavailability Score	0.55

Medicinal Chemistry	
PAINS	0 alert
Brenk	2 alerts: aldehyde, phenol_ester
Leadlikeness	Yes
Synthetic accessibility	2.25

Figure S5. ADME and physicochemical properties of compound **5**.

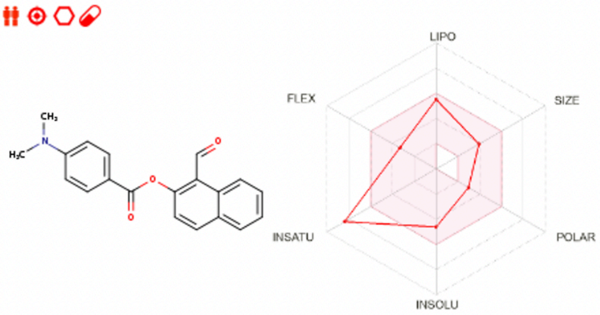

SMILES O=Cc1c(ccc2c1cccc2)OC(=O)c1ccc(cc1)N(C)C

Physicochemical Properties	
Formula	C20H17NO3
Molecular weight	319.35 g/mol
Num. heavy atoms	24
Num. arom. heavy atoms	16
Fraction Csp3	0.10
Num. rotatable bonds	5
Num. H-bond acceptors	3
Num. H-bond donors	0
Molar Refractivity	94.94
TPSA	46.61 Å²

Lipophilicity	
Log $P_{o/w}$ (iLOGP)	2.86
Log $P_{o/w}$ (XLOGP3)	4.17
Log $P_{o/w}$ (WLOGP)	3.94
Log $P_{o/w}$ (MLOGP)	3.40
Log $P_{o/w}$ (SILICOS-IT)	3.82
Consensus Log $P_{o/w}$	3.64

Water Solubility	
Log *S* (ESOL)	-4.61
Solubility	7.83e-03 mg/ml ; 2.45e-05 mol/l
Class	Moderately soluble
Log *S* (Ali)	-4.86
Solubility	4.44e-03 mg/ml ; 1.39e-05 mol/l
Class	Moderately soluble
Log *S* (SILICOS-IT)	-6.32
Solubility	1.54e-04 mg/ml ; 4.81e-07 mol/l
Class	Poorly soluble

Pharmacokinetics	
GI absorption	High
BBB permeant	Yes
P-gp substrate	No
CYP1A2 inhibitor	Yes
CYP2C19 inhibitor	Yes
CYP2C9 inhibitor	Yes
CYP2D6 inhibitor	No
CYP3A4 inhibitor	No
Log K_p (skin permeation)	-5.29 cm/s

Druglikeness	
Lipinski	Yes; 0 violation
Ghose	Yes
Veber	Yes
Egan	Yes
Muegge	Yes
Bioavailability Score	0.55

Medicinal Chemistry	
PAINS	0 alert
Brenk	2 alerts: aldehyde, phenol_ester
Leadlikeness	No; 1 violation: XLOGP3>3.5
Synthetic accessibility	2.29

Figure S6. ADME and physicochemical properties of compound **6**.

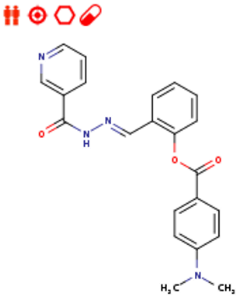

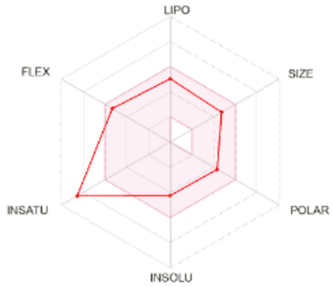

SMILES O=C(c1ccc(cc1)N(C)C)Oc1ccccc1/C=N/NC(=O)c1cccnc1

Physicochemical Properties	
Formula	C22H20N4O3
Molecular weight	388.42 g/mol
Num. heavy atoms	29
Num. arom. heavy atoms	18
Fraction Csp3	0.09
Num. rotatable bonds	8
Num. H-bond acceptors	5
Num. H-bond donors	1
Molar Refractivity	111.01
TPSA	83.89 Å^2

Lipophilicity	
Log $P_{o/w}$ (iLOGP)	2.45
Log $P_{o/w}$ (XLOGP3)	3.30
Log $P_{o/w}$ (WLOGP)	3.13
Log $P_{o/w}$ (MLOGP)	2.39
Log $P_{o/w}$ (SILICOS-IT)	3.16
Consensus Log $P_{o/w}$	2.88

Water Solubility	
Log *S* (ESOL)	-4.26
Solubility	2.14e-02 mg/ml ; 5.51e-05 mol/l
Class	Moderately soluble
Log *S* (Ali)	-4.74
Solubility	7.11e-03 mg/ml ; 1.83e-05 mol/l
Class	Moderately soluble
Log *S* (SILICOS-IT)	-6.88
Solubility	5.07e-05 mg/ml ; 1.31e-07 mol/l
Class	Poorly soluble

Pharmacokinetics	
GI absorption	High
BBB permeant	No
P-gp substrate	No
CYP1A2 inhibitor	No
CYP2C19 inhibitor	Yes
CYP2C9 inhibitor	Yes
CYP2D6 inhibitor	No
CYP3A4 inhibitor	Yes
Log K_p (skin permeation)	-6.33 cm/s

Druglikeness	
Lipinski	Yes; 0 violation
Ghose	Yes
Veber	Yes
Egan	Yes
Muegge	Yes
Bioavailability Score	0.55

Medicinal Chemistry	
PAINS	0 alert
Brenk	2 alerts: imine_1, phenol_ester
Leadlikeness	No; 2 violations: MW>350, Rotors>7
Synthetic accessibility	3.12

Figure S7. ADME and physicochemical properties of compound **7**.

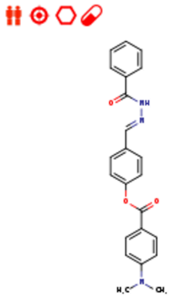

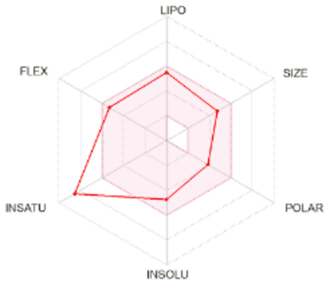

SMILES CN(c1ccc(cc1)C(=O)Oc1ccc(cc1)/C=N/NC(=O)c1ccccc1)C

Physicochemical Properties	
Formula	C23H21N3O3
Molecular weight	387.43 g/mol
Num. heavy atoms	29
Num. arom. heavy atoms	18
Fraction Csp3	0.09
Num. rotatable bonds	8
Num. H-bond acceptors	4
Num. H-bond donors	1
Molar Refractivity	113.22
TPSA	71.00 Å²

Lipophilicity	
Log $P_{o/w}$ (iLOGP)	3.36
Log $P_{o/w}$ (XLOGP3)	4.09
Log $P_{o/w}$ (WLOGP)	3.74
Log $P_{o/w}$ (MLOGP)	3.79
Log $P_{o/w}$ (SILICOS-IT)	3.73
Consensus Log $P_{o/w}$	3.74

Water Solubility	
Log *S* (ESOL)	-4.75
Solubility	6.89e-03 mg/ml ; 1.78e-05 mol/l
Class	Moderately soluble
Log *S* (Ali)	-5.29
Solubility	2.00e-03 mg/ml ; 5.17e-06 mol/l
Class	Moderately soluble
Log *S* (SILICOS-IT)	-7.26
Solubility	2.14e-05 mg/ml ; 5.53e-08 mol/l
Class	Poorly soluble

Pharmacokinetics	
GI absorption	High
BBB permeant	Yes
P-gp substrate	No
CYP1A2 inhibitor	No
CYP2C19 inhibitor	Yes
CYP2C9 inhibitor	Yes
CYP2D6 inhibitor	No
CYP3A4 inhibitor	No
Log K_p (skin permeation)	-5.76 cm/s

Druglikeness	
Lipinski	Yes; 0 violation
Ghose	Yes
Veber	Yes
Egan	Yes
Muegge	Yes
Bioavailability Score	0.55

Medicinal Chemistry	
PAINS	0 alert
Brenk	2 alerts: imine_1, phenol_ester
Leadlikeness	No; 3 violations: MW>350, Rotors>7, XLOGP3>3.5
Synthetic accessibility	2.97

Figure S8. ADME and physicochemical properties of compound **8**.

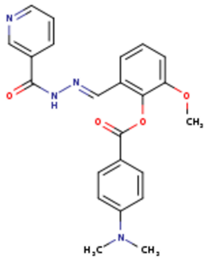

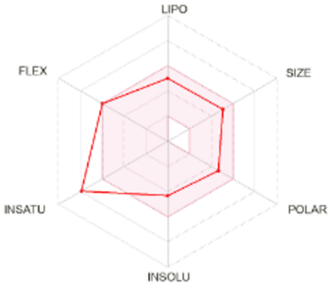

SMILES COc1cccc(c1OC(=O)c1ccc(cc1)N(C)C)/C=N/NC(=O)c1cccnc1

Physicochemical Properties	
Formula	C23H22N4O4
Molecular weight	418.45 g/mol
Num. heavy atoms	31
Num. arom. heavy atoms	18
Fraction Csp3	0.13
Num. rotatable bonds	9
Num. H-bond acceptors	6
Num. H-bond donors	1
Molar Refractivity	117.50
TPSA	93.12 Å²

Lipophilicity	
Log $P_{o/w}$ (iLOGP)	2.71
Log $P_{o/w}$ (XLOGP3)	3.27
Log $P_{o/w}$ (WLOGP)	3.14
Log $P_{o/w}$ (MLOGP)	1.81
Log $P_{o/w}$ (SILICOS-IT)	3.24
Consensus Log $P_{o/w}$	2.83

Water Solubility	
Log *S* (ESOL)	-4.33
Solubility	1.96e-02 mg/ml ; 4.68e-05 mol/l
Class	Moderately soluble
Log *S* (Ali)	-4.90
Solubility	5.27e-03 mg/ml ; 1.26e-05 mol/l
Class	Moderately soluble
Log *S* (SILICOS-IT)	-6.99
Solubility	4.32e-05 mg/ml ; 1.03e-07 mol/l
Class	Poorly soluble

Pharmacokinetics	
GI absorption	High
BBB permeant	No
P-gp substrate	No
CYP1A2 inhibitor	No
CYP2C19 inhibitor	Yes
CYP2C9 inhibitor	Yes
CYP2D6 inhibitor	No
CYP3A4 inhibitor	Yes
Log K_p (skin permeation)	-6.53 cm/s

Druglikeness	
Lipinski	Yes; 0 violation
Ghose	Yes
Veber	Yes
Egan	Yes
Muegge	Yes
Bioavailability Score	0.55

Medicinal Chemistry	
PAINS	0 alert
Brenk	2 alerts: imine_1, phenol_ester
Leadlikeness	No; 2 violations: MW>350, Rotors>7
Synthetic accessibility	3.31

Figure S9. ADME and physicochemical properties of compound **9**.

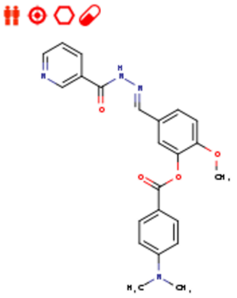

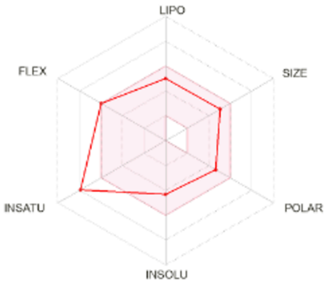

SMILES COc1ccc(cc1OC(=O)c1ccc(cc1)N(C)C)/C=N/NC(=O)c1cccnc1

Physicochemical Properties	
Formula	C23H22N4O4
Molecular weight	418.45 g/mol
Num. heavy atoms	31
Num. arom. heavy atoms	18
Fraction Csp3	0.13
Num. rotatable bonds	9
Num. H-bond acceptors	6
Num. H-bond donors	1
Molar Refractivity	117.50
TPSA	93.12 Å²

Lipophilicity	
Log $P_{o/w}$ (iLOGP)	3.24
Log $P_{o/w}$ (XLOGP3)	3.27
Log $P_{o/w}$ (WLOGP)	3.14
Log $P_{o/w}$ (MLOGP)	1.81
Log $P_{o/w}$ (SILICOS-IT)	3.24
Consensus Log $P_{o/w}$	2.94

Water Solubility	
Log *S* (ESOL)	-4.33
Solubility	1.96e-02 mg/ml ; 4.68e-05 mol/l
Class	Moderately soluble
Log *S* (Ali)	-4.90
Solubility	5.27e-03 mg/ml ; 1.26e-05 mol/l
Class	Moderately soluble
Log *S* (SILICOS-IT)	-6.99
Solubility	4.32e-05 mg/ml ; 1.03e-07 mol/l
Class	Poorly soluble

Pharmacokinetics	
GI absorption	High
BBB permeant	No
P-gp substrate	No
CYP1A2 inhibitor	No
CYP2C19 inhibitor	Yes
CYP2C9 inhibitor	Yes
CYP2D6 inhibitor	No
CYP3A4 inhibitor	Yes
Log K_p (skin permeation)	-6.53 cm/s

Druglikeness	
Lipinski	Yes; 0 violation
Ghose	Yes
Veber	Yes
Egan	Yes
Muegge	Yes
Bioavailability Score	0.55

Medicinal Chemistry	
PAINS	0 alert
Brenk	2 alerts: imine_1, phenol_ester
Leadlikeness	No; 2 violations: MW>350, Rotors>7
Synthetic accessibility	3.21

Figure S10. ADME and physicochemical properties of compound **10**.

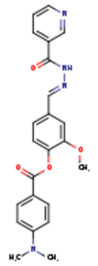

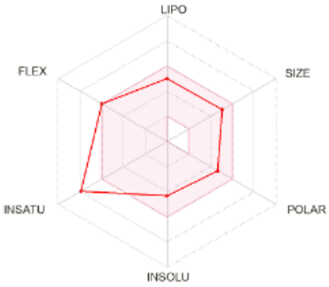

SMILES COc1cc(/C=N/NC(=O)c2cccnc2)ccc1OC(=O)c1ccc(cc1)N(C)C

Physicochemical Properties	
Formula	C23H22N4O4
Molecular weight	418.45 g/mol
Num. heavy atoms	31
Num. arom. heavy atoms	18
Fraction Csp3	0.13
Num. rotatable bonds	9
Num. H-bond acceptors	6
Num. H-bond donors	1
Molar Refractivity	117.50
TPSA	93.12 Å²

Lipophilicity	
Log $P_{o/w}$ (iLOGP)	3.07
Log $P_{o/w}$ (XLOGP3)	3.27
Log $P_{o/w}$ (WLOGP)	3.14
Log $P_{o/w}$ (MLOGP)	1.81
Log $P_{o/w}$ (SILICOS-IT)	3.24
Consensus Log $P_{o/w}$	2.91

Water Solubility	
Log *S* (ESOL)	-4.33
Solubility	1.96e-02 mg/ml ; 4.68e-05 mol/l
Class	Moderately soluble
Log *S* (Ali)	-4.90
Solubility	5.27e-03 mg/ml ; 1.26e-05 mol/l
Class	Moderately soluble
Log *S* (SILICOS-IT)	-6.99
Solubility	4.32e-05 mg/ml ; 1.03e-07 mol/l
Class	Poorly soluble

Pharmacokinetics	
GI absorption	High
BBB permeant	No
P-gp substrate	No
CYP1A2 inhibitor	No
CYP2C19 inhibitor	Yes
CYP2C9 inhibitor	Yes
CYP2D6 inhibitor	No
CYP3A4 inhibitor	Yes
Log K_p (skin permeation)	-6.53 cm/s

Druglikeness	
Lipinski	Yes; 0 violation
Ghose	Yes
Veber	Yes
Egan	Yes
Muegge	Yes
Bioavailability Score	0.55

Medicinal Chemistry	
PAINS	0 alert
Brenk	2 alerts: imine_1, phenol_ester
Leadlikeness	No; 2 violations: MW>350, Rotors>7
Synthetic accessibility	3.18

Figure S11. ADME and physicochemical properties of compound **11**.

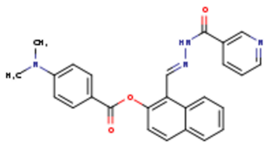

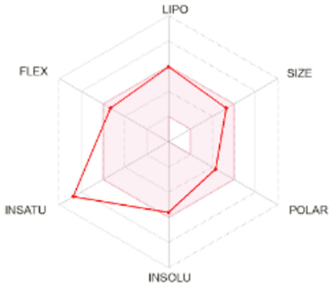

SMILES O=C(c1ccc(cc1)N(C)C)Oc1ccc2c(c1/C=N/NC(=O)c1cccnc1)cccc2

Physicochemical Properties	
Formula	C26H22N4O3
Molecular weight	438.48 g/mol
Num. heavy atoms	33
Num. arom. heavy atoms	22
Fraction Csp3	0.08
Num. rotatable bonds	8
Num. H-bond acceptors	5
Num. H-bond donors	1
Molar Refractivity	128.52
TPSA	83.89 Å^2

Lipophilicity	
Log $P_{o/w}$ (iLOGP)	3.13
Log $P_{o/w}$ (XLOGP3)	4.91
Log $P_{o/w}$ (WLOGP)	4.28
Log $P_{o/w}$ (MLOGP)	3.05
Log $P_{o/w}$ (SILICOS-IT)	4.20
Consensus Log $P_{o/w}$	3.92

Water Solubility	
Log *S* (ESOL)	-5.62
Solubility	1.06e-03 mg/ml ; 2.41e-06 mol/l
Class	Moderately soluble
Log *S* (Ali)	-6.41
Solubility	1.71e-04 mg/ml ; 3.91e-07 mol/l
Class	Poorly soluble
Log *S* (SILICOS-IT)	-8.52
Solubility	1.34e-06 mg/ml ; 3.05e-09 mol/l
Class	Poorly soluble

Pharmacokinetics	
GI absorption	High
BBB permeant	No
P-gp substrate	No
CYP1A2 inhibitor	No
CYP2C19 inhibitor	Yes
CYP2C9 inhibitor	Yes
CYP2D6 inhibitor	No
CYP3A4 inhibitor	Yes
Log K_p (skin permeation)	-5.49 cm/s

Druglikeness	
Lipinski	Yes; 0 violation
Ghose	Yes
Veber	Yes
Egan	Yes
Muegge	Yes
Bioavailability Score	0.55

Medicinal Chemistry	
PAINS	0 alert
Brenk	2 alerts: imine_1, phenol_ester
Leadlikeness	No; 3 violations: MW>350, Rotors>7, XLOGP3>3.5
Synthetic accessibility	3.36

Figure S12. ADME and physicochemical properties of compound **12**.
